# Annotated 18S and 28S rDNA reference sequences of taxa in the planktonic diatom family Chaetocerotaceae

**DOI:** 10.1371/journal.pone.0208929

**Published:** 2018-12-26

**Authors:** Chetan C. Gaonkar, Roberta Piredda, Carmen Minucci, David G. Mann, Marina Montresor, Diana Sarno, Wiebe H. C. F. Kooistra

**Affiliations:** 1 Integrative Marine Ecology Department, Stazione Zoologica Anton Dohrn, Napoli, Italy; 2 Royal Botanic Garden Edinburgh, Edinburgh, Scotland, United Kingdom, and Institut de Recerca i Tecnologia Agroalimentaries, Sant Carles de La Ràpita, Catalonia, Spain; National Cheng Kung University, TAIWAN

## Abstract

The species-rich diatom family Chaetocerotaceae is common in the coastal marine phytoplankton worldwide where it is responsible for a substantial part of the primary production. Despite its relevance for the global cycling of carbon and silica, many species are still described only morphologically, and numerous specimens do not fit any described taxa. Nowadays, studies to assess plankton biodiversity deploy high throughput sequencing metabarcoding of the 18S rDNA V4 region, but to translate the gathered metabarcodes into biologically meaningful taxa, there is a need for reference barcodes. However, 18S reference barcodes for this important family are still relatively scarce. We provide 18S rDNA and partial 28S rDNA reference sequences of 443 morphologically characterized chaetocerotacean strains. We gathered 164 of the 216 18S sequences and 244 of the 413 28S sequences of strains from the Gulf of Naples, Atlantic France, and Chile. Inferred phylogenies showed 84 terminal taxa in seven principal clades. Two of these clades included terminal taxa whose rDNA sequences contained spliceosomal and Group IC1 introns. Regarding the commonly used metabarcode markers in planktonic diversity studies, all terminal taxa can be discriminated with the 18S V4 hypervariable region; its primers fit their targets in all but two species, and the V4-tree topology is similar to that of the 18S. Hence V4-metabarcodes of unknown Chaetocerotaceae are assignable to the family. Regarding the V9 hypervariable region, most terminal taxa can be discriminated, but several contain introns in their primer targets. Moreover, poor phylogenetic resolution of the V9 region affects placement of metabarcodes of putative but unknown chaetocerotacean taxa, and hence, uncertainty in taxonomic assignment, even of higher taxa.

## Introduction

Phytoplankton diversity in environmental samples is recorded routinely through light microscopic (LM) identification and counting. Yet, this approach is cost- and labor-intensive, requires expert taxonomic knowledge, and does not allow the identification of all the taxa. A morphology-independent alternative is high throughput sequencing (HTS) metabarcoding of environmental samples and translation of the resulting metabarcodes into relative percentages of taxa in a sample (e.g., [1]; [2]; [3]). As the cost of HTS diminishes steadily, this approach constitutes an alternative to cell counting, or—for the sake of continuity—an approach alongside it. However, translation of metabarcode information into species presence requires comprehensive datasets of reference barcodes (e.g., DINOREF [4]; PR2 [5]; SILVA [6]; BOLD [7]). Ideally, such references constitute marker sequences from strains for which biological information such as LM, scanning and transmission electron microscopy (SEM and TEM) imagery is available as well.

The principal aim of the present study is to provide a comprehensive dataset of such reference barcodes for taxa in Chaetocerotaceae. This species-rich diatom family is common in the marine phytoplankton worldwide, in particular in coastal regions, upwelling zones, and in the Southern Ocean (e.g., [8], [9], [10]), where it is responsible for a substantial part of the primary production. Its resting spores descend on the sediment, where they can remain dormant for several years. Not surprisingly, species in Chaetocerotaceae constitute major drivers in the global cycling of carbon and silica [11].

The family includes two extant genera, *Bacteriastrum* Shadbolt and *Chaetoceros* Ehrenberg [12]. *Chaetoceros* is abundant and diverse, with well over 200 described species [13] whereas *Bacteriastrum* is less diverse, with eleven accepted species [13]. The main distinguishing character of the two genera is that the siliceous projections, called setae, which ornament the two valve-elements of each cell wall, have a radial arrangement around the valve margin in *Bacteriastrum*, while in *Chaetoceros* only two setae emerge from each valve. In both genera, cells are generally joined together via the basal portion of the setae, although predominantly single-celled species do exist as well. The shape of the colony and of the aperture between adjacent cells, the morphology of the terminal and intercalary setae, and the number of chloroplasts and their presence/absence in the setae constitute the main morphological characters for species identification in LM [14]. Ultrastructural features only visible in electron microscopy, such as shape and position of the rimoportula, ultrastructure of the valve, and ornamentation of the setae, provide additional characters for species identification. *Chaetoceros* was traditionally divided in two subgenera [15]: *Phaeoceros* (also reported as subgenus *Chaetoceros*; [16]), containing robust forms that have plastids in the setae, and *Hyalochaetae*, comprising less silicified forms that lack plastids in the setae. The formation of resting spores is reported for a single *Bacteriastrum* species, i.e., *B*. *hyalinum* [17], and several *Hyalochaetae* species [18] and their morphology and ultrastructure also are a source of distinctive species-specific diagnostic features.

Molecular data have hitherto been available for only some of the already described species in the Chaetocerotaceae. In addition, the regularity with which species new to science are described (e.g., [19]; [20]; [21]; [22]) and cryptic diversity is uncovered (e.g., [23]; [24]; [25]; [26]; [27]; [28]; [29]) suggests that a considerable part of the diversity in this family is still to be revealed.

As a reference barcode for our study, we focused on the nuclear-encoded SSU ribosomal RNA gene (from here onwards 18S) because its hypervariable V4 and V9 regions have been applied in many metabarcode studies of protistan diversity (e.g., [30]; [31]; [1]; [2]; [3]; [32]). Chaetocerotacean 18S reference barcodes are still underrepresented because most of the recent taxonomic studies in this family used, instead, a ca. 700 bp region at the 5′-end of the nuclear-encoded LSU rRNA gene (from here onwards 28S) as barcode (e.g., [17]; [24]; [25]; [33]; [27]; [28]; [19]). Here, we obtained the entire 18S to enable phylogenetic inference and to allow the universality of any potential metabarcode primer to be checked across the family. We also gathered the 28S for phylogenetic purposes and to enable comparison with already described taxa for which only that marker was sequenced. Cell morphology and frustule ultrastructure of different taxa was documented based on selected reference strains.

We focused our exploration on the Long Term Ecological Research station MareChiara (LTER MC) in the Gulf of Naples because of its high chaetocerotacean diversity, abundance and marked seasonality [34]. Yet, we also examined strains collected along the Chilean coast and at the French Atlantic coast. We included 18S and 28S sequences from other studies if these also detailed the morphology of the sequenced strains.

The gathered sequences of 443 morphologically characterized chaetocerotacean strains grouped into 84 terminal taxa and seven principal clades. Two of these clades included terminal taxa containing spliceosomal and Group IC1 introns in their rDNA sequences. Virtually all the terminal taxa can be discriminated with the V4 hypervariable region and the V4 primers fit their targets in all but two of them. Since the V4 reference barcodes of the known Chaetocerotaceae form a clade, metabarcodes of unknown Chaetocerotaceae can be expected to group within the family as well. Regarding the V9 hypervariable region, most terminal taxa can be discriminated, but several contain introns in their primer targets, affecting their detection. Moreover, poor phylogenetic resolution affects accurate placement of metabarcodes for which no close reference sequence is available.

## Materials and methods

### Strains isolation and culturing

Plankton net samples were gathered: i) in the Gulf of Naples, Tyrrhenian Sea, Italy in the frame of the ongoing research at the LTER station MareChiara; ii) a few km offshore from Las Cruces and from San Antonio, Chile within the frame of the EU FP7-funded project ASSEMBLE (Grant Agreement No 227799) and its agreements with the Pontifical University of Chile, Santiago de Chile, iii) offshore from Concepción, Chile in the framework of the ongoing research at the COPAS Oceanographic Time Series station; iv) at the Estacade, Station Biologique de Roscoff, France and v) at the LTER station of the Inter University Institute, Eilat, Gulf of Aqaba, Israel, both in the frame of a collaboration in the EU H2020 funded cluster project EMBRIC (Grant Agreement No 654008). Monoclonal strains of Chaetocerotaceae were established by isolating individual cells or chains from these samples using glass capillaries and an inverted LM. Isolated strains were incubated in f/2 marine enrichment medium [35] prepared using Guillard’s (f/2) Marine Water Enrichment Solution (Sigma-Aldrich, St. Louis, USA) in 12-well tissue culture plates (Costar 3513; Corning Incorporated, NY, USA). The strains (S1 Table) were maintained in 74 ml polystyrene cell culture flasks (Corning Inc., NY, USA) filled with 30 ml of f/2 medium adjusted to a salinity of 36, at 15 °C, with a 12:12 h light:dark cycle and a photon flux density of 50 μmol m^−2^ s^−1^ provided by cool white (40 W) fluorescent tubes.

### Molecular characterization

#### DNA extraction, PCR-amplification and sequencing

Genomic DNA was extracted with a CTAB extraction protocol (modified from [36] as described in [37]). The 28S (ca. 750 bp at 5’-end) and 18S sequences were PCR-amplified using Roche DNA Polymerase (Roche Diagnostics GmbH, Mannheim, Germany) and Sanger-sequenced as described in [26]. In case PCR-amplification of the entire 18S in a single product failed, the sequence was obtained in two or three overlapping products using various combinations of primers listed in S2 Table.

If that failed as well, PCR-amplification was carried out using high fidelity Phusion DNA polymerase (New England BioLabs Inc, Massachusetts, USA). Reaction mixture (20 μl) contained 4 μl 5X Phusion HF or GC Buffer, 0.4 μl (200 μM) of 10 mM dNTPs, 1 μl (0.5 μM) of each 10 μM primer, 0.6 μl (3%) of DMSO, 50–250 ng genomic DNA, and 0.2 μl (1 U) polymerase. Annealing temperatures for primer pairs were determined using the online New England Biolabs Tm calculator tool (https://tmcalculator.neb.com/#!/main). PCR was conducted on a preheated (98 °C) thermocycler as follows: 60 s initial denaturation at 98 °C, 35 cycles of 30 s at 98° C, 20 s at the calculated annealing temperature and 35 s at 72 °C, followed by 10 min at final extension of 72 °C. PCR products were sequenced and the resulting forward and reverse reads concatenated as described in [26]. Sequences are available in GenBank (S1 Table).

#### Sequence alignment

In the sequence analyses, we included information from strains presented in other studies only if both their 18S and the partial 28S and their morphological descriptions were available, with a few exceptions (e.g., *Chaetoceros dayaensis* in [19]), for which only 28S was available. Sequences were aligned using MAFFT v7.245 ([38]) under default settings and output-in-alignment-order and adjusted manually using SeaView v4.5.4 ([39]) or Sequence Alignment Editor v2.0a11 ([40]; (http://tree.bio.ed.ac.uk/software/seal/). As outgroups, the 18S and 28S sequences of phylogenetically related bi- and multipolar centric diatoms were used (e.g., [41]; [42]) (S1 Table). Positions showing ambiguous alignment and positions representing gaps in all but one of the sequences were excluded from phylogenetic analyses, and so were introns and the frayed 5′- and 3′-ends of the alignment.

#### Phylogenetic analysis

Maximum likelihood (ML) trees were inferred from the 18S as well as from the 28S alignment of all gathered sequences using FastTree [43] to obtain an overview of the sequence diversity and to delineate terminal taxa. A terminal taxon is defined here as a clade consisting of a group of identical and near-identical sequences, exhibiting little or no internal phylogenetic structure. Likewise, ML analysis was carried out on only the 18S V4 region -excluding its primer target regions- to assess if terminal taxa in the 18S tree can be identified in the resulting tree as well and if *Bacteriastrum* and *Chaetoceros* form clades. The same was done for the V9 region.

Subsequent analyses were carried out including only (single) representative sequences of terminal taxa. Whenever possible, representative 18S and 28S sequences from the same strain were chosen. ML trees were inferred with RAxML [44] as implemented in raxml GUI v.1.5beta ([45]), using a GTRGAMMA substitution model and bootstrap analysis with 1000 replicates. Bayesian trees were inferred using MrBayes 3.2.2 on XSEDE [46] with a GTR+r model. The analysis started with a random tree. The posterior probability of the phylogenetic model was estimated using Markov chain Monte Carlo (MCMC). Four chains were run, three heated and one cold and sampled every 100 generations. To determine the run length, convergence onto the stationary distribution was assessed using the standard deviation of split frequencies. After the ‘burn-in’, in the 18S and the 28S dataset, the initial 1500 trees were removed from the dataset and the remaining 8500 trees were used to produce the majority-rule consensus trees.

### Morphological documentation and strain identification

Cell morphology and frustule ultrastructure of strains gathered in this study were documented by means of LM, SEM and TEM imaging as described in [26]. Strains whose core sequences grouped together in a terminal clade and which shared the same morphology and ultrastructure were considered to be conspecific, whereas different terminal clades were considered distinct species regardless of whether or not their strains could be separated morphologically.

S1 Supporting Information provides an explanation of the morphological concept of each taxon (= terminal clade). It includes relevant literature references for species identification and a brief morphological characterization for: i) the taxa for which molecular information was not available; ii) the taxa that do not match to known species; and iii) the cryptic taxa detected in this study. Sequences and morphology of the terminal taxa were compared with those of species described in the literature. Terminal taxa were categorized as specified below.

Type A: terminal taxa whose morphology and 18S and/or 28S sequences matched those reported in previous publications (additional reference/s). In this case we have included also those reported in the original publication. Terminal taxa marked A* belong to formally described species for which 28S and/or 18S sequences are available in the literature, but for which material was not obtained in the present study.
Type B: terminal taxa whose morphology matched that of species for which ultrastructural studies have been published (additional reference/s) but no 18S or 28S sequences were available previous to the present study.
Type C: groups of terminal taxa in 18S and 28S, which are morphologically identical to known species. The different terminal taxa have been named “species name 1, 2, 3” because it remains to be determined which one conforms to the known species and which one/s should be described as (a) new species.
Type D: terminal taxa morphologically similar (but not identical) to a known species. These were identified as “cf. species name”.
Type E: terminal taxa whose morphology did not fit any known species. These taxa were identified with the genus name (‘*Bacteriastrum* sp.’ or ‘*Chaetoceros* sp.’) followed by the code of a representative strain for the genotype/clade and then by the code of the particular strain itself.

## Results

### Alignments

A total of 443 chaetocerotacean strains (20 for *Bacteriastrum* and 423 for *Chaetoceros*) were considered in this study. The 18S dataset consisted of 245 sequences (14 *Bacteriastrum*, 202 *Chaetoceros*, 29 outgroup taxa) of which 164 ingroup sequences were produced in this study (S1 Table). They usually exhibited a length between 1669 and 1703 bp, though several of them contained one or multiple inserts, markedly increasing their length (Table 1). Alignment of the ingroup sequences with 29 outgroup sequences resulted in an alignment matrix of 5550 positions with inserts at 19 locations (S1 Fig). Removal of inserts resulted in an alignment of 1719 positions (frayed 5′- and 3′-ends excluded). The partial 28S dataset consisted of 426 sequences (18 *Bacteriastrum*, 395 *Chaetoceros*, 13 outgroup taxa) of which 244 ingroup sequences were produced in this study (S1 Table). The sequences were typically between 680 and 765bp in length, though several contained an insert of up to 205 bp. Alignment of the 28S core regions (frayed 5′- and 3′-ends excluded) required 780 positions, whereas an additional 205 positions were needed at a single location to accommodate inserts. Alignments of the 18S and partial 28S rDNA sequences including introns has been made available in FigShare at https://figshare.com/articles/Chaetoceros_LSU_and_SSU_reference_sequences_and_metadata_table/7275923.

**Table 1:** Position of group-I introns (GI) and spliceosomal introns (SP) in 18S and 28S sequences of Chaetocerotaceae.

Location	Family	Position	*B*. *jadranum*	*B*. *furcatum* PMFBA4	*B*. *furcatum* Na8A3	*B*. *hyalinum* CCMP141	*C*. sp. Clade Na13C2	*C*. sp. Clade CDP22	*C*. *circinalis*	*C*. sp. Clade Na17B2	*C*. sp. Clade Na12A3	*C*. *diversus* 1	*C*. *diversus* 2	*C*. *seiracanthus*	*C*. *rotosporus*	*C*. *decipiens* Na & MC	*C*. sp. Clade Na28A1	*C*. sp. Clade Va7-D2	*C*. *anastomosans*	*C*. *vixvisibilis* group 1	*C*. *vixvisibilis* group 2	Primers affected	Region affected
**18S**																							
1	SP	385–386										132	106+					?					
2	SP	442–443					90+	104		110	107	96	125					?					
3	SP	548–549					?	108										?					
4	GI	549–550	400	221+	399		?			360+	453		547	467	457	425	181+	?					
5	SP	888–889					130			124	110†											Ch-690F+R	V4
6	SP	889–890										121										Ch-690F+R	V4
7	SP	891–892		115		57+																Ch-690F+R	V4
8	SP	981–982						?			51+	123						?					
9	SP	989–990					162	?	100	107			160–199					?	123				
10	SP	1011–1012		92+			115	?			96	109	161				124	?				Ch-1147F+R	
11	SP	1147–1148		?			112	?		101	94	104	133‡				141	?					
12	GI	1151–1152		?				?						462–465	505–513			?					
13	SP	1195–1196		?			152	71+	110	100	104	105	121				141	?					
14	SP	1257–1258		?					?	*392+*								?				Ch-1055F+R	
15	SP	1274–1275		?			80	106	?	102			86–96				134	?			*342+*		
16	SP	1414–1415		?			117		106+	99	108	35+	153					?					
17	SP	1612–1613	?	?							98							?				Ch-1400F+R	
18	SP	1615–1616	?	?			115	116		118		117	96–97			15§		?		154	98–154	Ch-1400F+R & V9f	V9
19	SP	1760–1761	?	?	?	?	109+	?	?	?	?	91+	91+	?	?	94+	92+	?	73+	?	?	Ch-V9r	V9
		#Inserts	**1**	**3**	**1**	**1**	**10**	**5**	**3**	**10**	**9**	**10**	**11**	**2**	**2**	**3**	**6**		**2**	**1**	**2**		
		added Σ bp	400+	428+	399	57+	1073+	505	316+	1613+	1221+	1033+	1829+	932	970	534+	813+		196+	154	496+		
**28S**																							
1	SP	758–759					112			108	112–116		115				205+	94–105					

“Location” refers to order of appearance in the 18S and 28S sequence alignment. Family: SP, spliceosomal intron; GI, Group IC1 intron. “Position” refers to the positions of the 18S core nucleotides flanking the 5′- and 3′- ends of the insert in the 18S rRNA secondary structure model of *C*. *tenuissimus* strain CHMS01 (from http://www.rna.icmb.utexas.edu, there listed as *Chaetoceros* sp.; S1 Fig). Figures in the body of the table under the terminal taxa indicate the maximum length of the inserts of the strains belonging to that terminal taxon in that location (123, exact length; 123–134, length range; 123+ length of sequenced part, actual insert is longer; ‘?’ Presence of insert unknown because sequencing of region failed; *392+*, long SP, possibly SP inside another SP). Primers and marker regions (V4 and V9) affected by the insert are indicated to the right. ‘#inserts’ signifies the number of inserts in the 18S of the terminal taxon; ‘added Σ bp’ indicates the extra length, in bp, added by the inserts to the 18S core sequence.

^†^ Only *Chaetoceros* sp. Clade Na12A3 strain Na43B1

^‡^ Only *C*. *diversus* 2 strain Na56B3

^§^ Only *C*. *decipiens* strain Na12B4

### Phylogenies

#### Phylogenies inferred from the 18S core sequences

**Fig 1:**
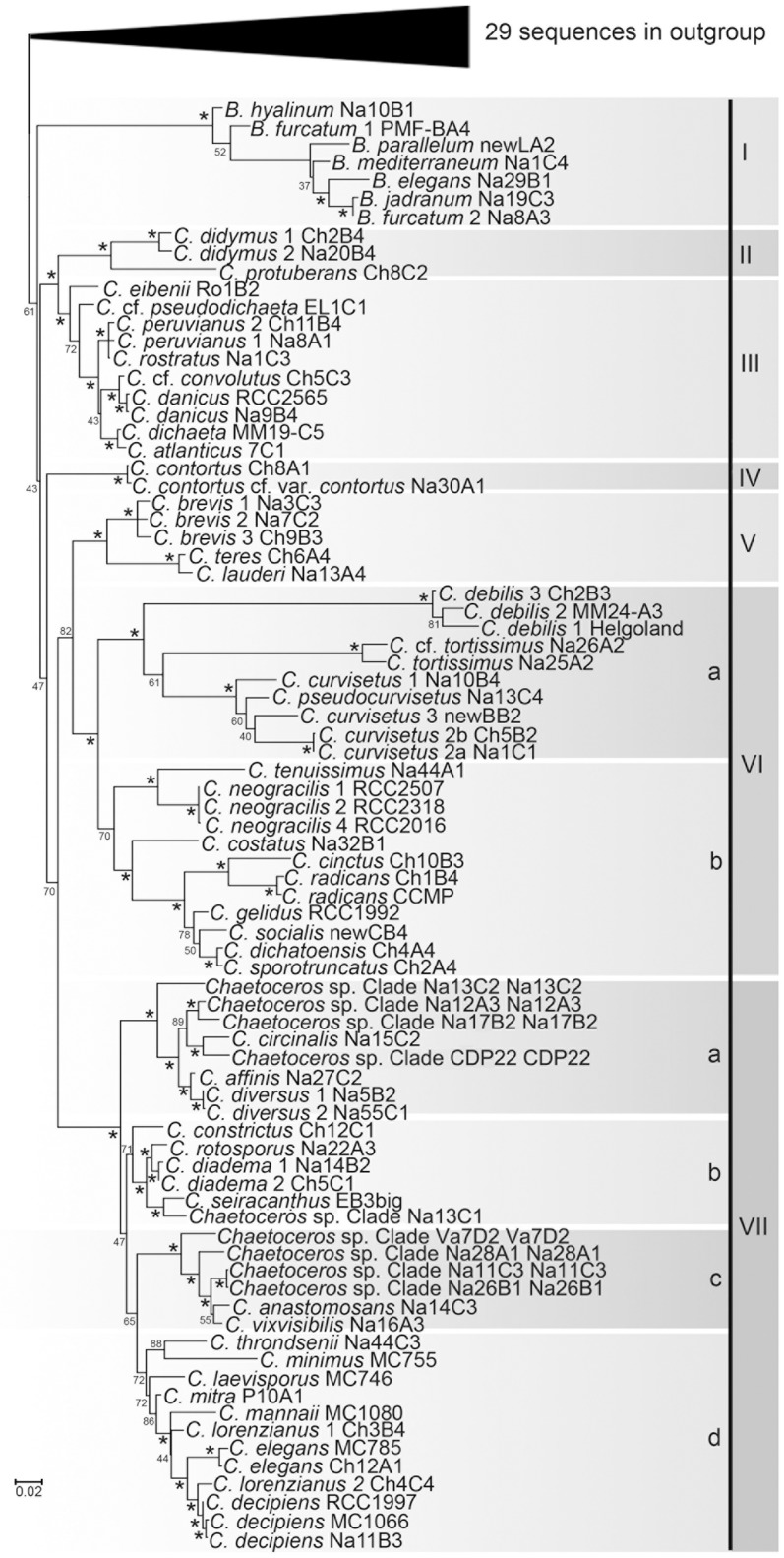
Maximum likelihood tree inferred with RAxML from 18S sequences of representative strains in terminal taxa in S2 Fig. Figures on the left side of clades are bootstrap values (1000 replicates); values ≥90% have been marked “*”. Major clades are indicated with Roman numerals and subclades with “a-d.” Strain codes: *Chaetoceros* spp represent species requiring taxonomic description; the first code refers to the representative strain of the Clade as a proxy for the species name, the second code refers to the actual strain.

In the 18S tree resulting from FastTree (S2 Fig) *Bacteriastrum* formed a clade, though it was not resolved as sister to a clade of *Chaetoceros*. The tree topology was generally well resolved with well-supported internal ramifications and terminal taxa, the latter showing little or no internal sequence variation. Virtually all of the morphologically defined species for which sequences of multiple strains were available were found to be monophyletic, although several of these morpho-species revealed two or more markedly distinct terminal taxa (i.e., cryptic species). There was a single exception to the monophyly; *B*. *furcatum* was polyphyletic (Fig 1; S2 Fig). Following selection of single representative sequences of terminal taxa, 95 chaetocerotacean ingroup sequences and the 29 outgroup sequences were retained for phylogenetic analysis. The resulting Maximum Likelihood tree (ML; Fig 1) and the Bayesian Inference tree (BI; S3 Fig) revealed a similar topology. The ingroup sequences formed a clade with weak bootstrap support (61%) but a high posterior probability (1.00) and were resolved into seven well-supported principal clades (numbered I-VII in Fig 1). In the following, a brief illustration of these clades is provided together with the main characteristics of the terminal taxa in each of them. Descriptions are reported in S1 Supporting Information and the photographic illustrations (Figs) reported therein are provided in S1 File.

Clade I included all *Bacteriastrum* sequences. Species in this genus share radial symmetry, multiple setae per valve, several plastids per cell and a rimoportula only in terminal valves.
Clade II included *C*. *protuberans* and *C*. *didymus*, which share valves with poroids and a pronounced central protuberance.
Clade III, which resolved as sister to Clade II, consisted of terminal taxa belonging to the subgenus *Phaeoceros*. The taxa in this clade, uniquely among Chaetocerotaceae, share the presence of plastids in the setae and a rimoportula on both terminal and intercalary valves; the only exception is *Chaetoceros* cf. *pseudodichaeta*, exhibiting a rimoportula only in the terminal valve.
Clade IV comprised the *C*. *contortus* complex. All species possess several chloroplasts per cell and most of the valves in a cell colony exhibit delicate setae. The defining feature of the taxa in this clade constitutes the occasional formation of more robust, intercalary setae on adjacent valves. Such specialized setae were only seen in field material; they did not develop in culture.
Clade V included *C*. *lauderi*, *C*. *teres* and the strains belonging to the *C*. *brevis* complex. The first two species share a similar gross morphology and the presence of multiple chloroplasts per cell. We did not identify any distinctive character for the cryptic species of *C*. *brevis*: all share a similar chain morphology, a single chloroplast per cell, globules inside the setae and a dark, more silicified area in the center of the valve.
Clade VI, comprising species with a single chloroplast per cell, was resolved as sister to Clade V and included in its turn two subclades. The first one (VIa) included three morphotypes characterized by curved or twisted chains: i) *C*. *debilis*, with three cryptic species all sharing curved and spiraling colonies; ii) *C*. *tortissimus* and *C*. cf. *tortissimus*, with chains twisted around the colony’s central axis; the two species share the presence of a large elongate hole at the base of the setae; iii) the *C*. *curvisetus* complex, including *C*. *pseudocurvisetus* and three species of *C*. *curvisetus*, all with curved and spiraling colonies. The second subclade (VIb) constituted a morphologically heterogeneous collection of taxa that do not share any unifying character: *C*. *tenuissimus*, the *C*. *neogracilis* complex, *C*. *costatus*, *C*. *radicans*. *C*. *cinctus* and the *C*. *socialis* complex.
Clade VII resolved into four sub-clades. The first one (VIIa) included taxa sharing a single chloroplast per cell, usually a narrow aperture, and a gross morphology resembling *C*. *affinis* (with terminal setae that are often more robust than intercalary ones). *Chaetoceros diversus* can be distinguished by the presence of pairs of specialized, more robust intercalary setae. However, these specialized setae gradually disappear in culture condition. The second subclade (VIIb) included i) *Chaetoceros constrictus* with two chloroplasts per cell and a marked constriction at the base of the valve mantle; ii) *C*. *seiracanthus*, *C*. *rotosporus*, the *C*. *diadema* complex and *Chaetoceros* sp. clade Na13C1, which share a similar chain morphology with wide apertures and have a single chloroplast. The third (VIIc) comprised *C*. cf. *vixvisibilis*, *C*. *anastomosans* and several undescribed species with narrow linear to oblong apertures between them, one or two chloroplasts per cell. The fourth subclade (VIId) included i) the diminutive, single-celled species *C*. *minimus* and *C*. *throndsenii*, which share the distinctive character of having only two setae per cell, as well as ii) the members of the *C*. *lorenzianus* complex exhibiting multiple chloroplasts per cell and setae with large pores.

#### Phylogenies inferred from the 28S core sequences

The 28S tree resulting from FastTree (S4 Fig) was topologically similar to the 18S tree, except that *Bacteriastrum* formed a clade inside a paraphyletic *Chaetoceros*, with *Hemiaulus* and *Dactyliosolen* as sister clade to the *C*. *contortus* complex.

**Fig 2:**
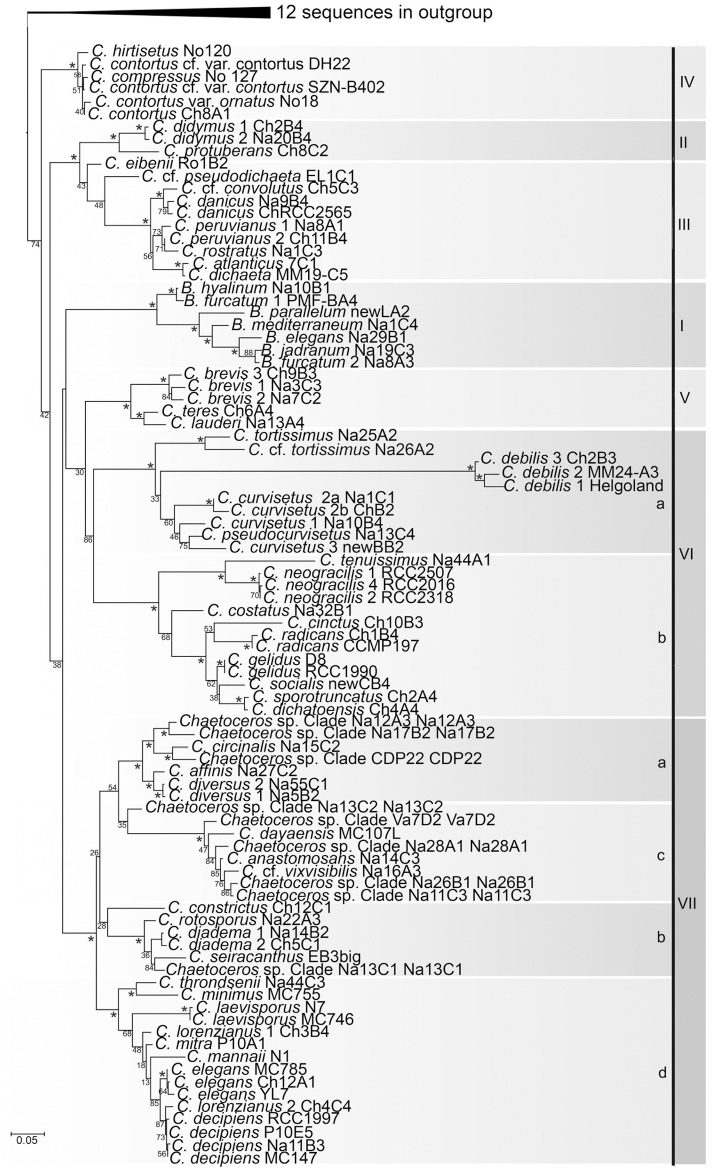
Maximum likelihood tree inferred with RAxML from partial 28S sequences of representative strains in terminal taxa in S4 Fig. Figures on the left side of clades are bootstrap values (1000 replicates); values ≥90% have been marked “*”. Major clades are indicated with Roman numerals and subclades with “a-d.” For explanations, see text. Strain codes: *Chaetoceros* spp represent species requiring taxonomic description; the first code refers to the representative strain of the Clade, the second code to the actual strain.

Following selection of representative sequences of terminal taxa, 104 chaetocerotacean ingroup sequences and 11 outgroup sequences were retained for phylogenetic analysis. The number of 28S ingroup sequences was slightly higher than in the 18S tree because of micro-variation and the inclusion of species for which 18S sequences were unavailable. The resulting ML tree (Fig 2) and BI tree (S5 Fig) had a highly similar topology. Chaetocerotacean sequences formed a clade though with insufficient support (48%, 0.92). In spite of that, the seven principal clades in the 18S trees were recovered in the 28S trees (Fig 2) as well and obtained high support. Ramifications inside these clades were basically the same as in the 18S trees and so were the sister relationships between Clades II and III and between Clades V and VI. *Bacteriastrum* was recovered inside *Chaetoceros*, as in the analysis of all sequences; in the ML tree (Fig 2) it was sister to the clade containing Clades V and VI whereas in the BI tree it was resolved as sister to Clade VII, though none of these sister relationships obtained sufficient support.

### Inserts

Inserts were detected at 19 locations in the 18S alignment and at one in the 28S alignment (Table 1, S1 Fig). In both markers they were found only in sequences grouping in Clades I and VII (Table 1). Some of the locations in the 18S were situated one or a few positions apart (S1 Fig). Ten locations were situated inside primer target regions. Presence of an insert in such a region resulted in PCR-failure when using the corresponding primer. Identical core sequences shared identical or near-identical inserts, though with two exceptions: sequences of *C*. cf. *vixvisibilis* and *C*. *diversus* each formed two groups with distinct insert profiles (Table 1). The 18S of strains Na12B4 (*C*. *decipiens*), Na43B1 (*Chaetoceros* sp. Clade Na12A3) and Na56B3 (*C*. *diversus* 2) exhibited intra-individual variation; PCR produced two bands, one with an (extra) insert and one without it. The inserts grouped into two length classes; long ones and short ones. The inserts added substantially to the overall length of the affected core sequences, e.g., the summed length of the eleven inserts in the 18S of *C*. *diversus* 2 (at least 1829 bp) exceeded the length of the 18S itself (Table 1)

#### Long inserts

Long inserts (399–547 bp) found at locations #4 and #12 in the 18S alignment (Table 1), revealed regions of similarity interspersed with unalignable regions. BLAST searches using the conserved parts returned group I introns in the rDNA of green and red algae, euglenozoans, pelagophytes, fungi, and the diatom *Hyalosira* sp. (AY485501). Alignment of conserved regions with their homologues in group IC1 and group IE introns from http://www.rna.icmb.utexas.edu/SIM/4E/Red_Brown/ and from GenBank and subsequent Neighbor-Joining analysis of pairwise dissimilarities resulted in a phylogram (not shown) in which chaetocerotacean inserts formed two separate groups within the group IC1 introns: one group with those at location #4 and the other one with those at location #12. The *Bacteriastrum* #4 inserts grouped with those of *C*. *decipiens*, *Chaetoceros* sp. Na12A3 and *C*. *rotosporus* in a sister group and *C*. *seiracanthus* as next nearest neighbor. The #12 inserts of *C*. *seiracanthus* and *C*. *rotosporus* formed a group with the one of *Hyalosira* sp. (AY485501) as nearest sister.

#### Short inserts

Short inserts (typically 96–199 bp) were recovered at 17 locations in the 18S and one in the 28S (Table 1). All exhibited a consensus GTDHNN (usually GTAAGT) at their 5′-end, followed by a highly variable region, a consensus YTRAC (commonly YTAAC), a highly variable CT-rich region, and a consensus YNHAG (usually YAYAG) at their 3′-end; all features typical for spliceosomal introns. Two longer inserts, one at locations #14 in *Chaetoceros* sp. Clade Na17B2 and one at location #15 in *C*. cf. *vixvisibilis* exhibited the typical 5′-end (GTAATA and GTACGT, respectively). Short inserts at the same location in different species often shared highly similar 5′-ends and 3′-ends. All inserts in the 28S shared consensus YTAMAG (mostly TTAAAG) at the 3′-end, which was not found among any of the 18S inserts.

### The barcode regions

#### The V4 region

The V4 region exhibited spliceosomal introns at location #5 in *Chaetoceros* spp Clade Na12A3, Clade Na13C2 and Clade 17B2, at location #6 in *C*. *diversus* 1, and at location #7 in *B*. *furcatum* 1 as well as in *B*. *hyalinum* strain CCMP141 but not in the Neapolitan strains belonging to this species (Table 1). None of these introns exceeded 124 bp in length. The V4-primers were found to fit their intended target regions in the chaetocerotacean 18S sequences, with just a few exceptions (S3 Table); critical mismatches occurred between the reverse primer (TAR-EukR; S2 Table), near its 3′-end, and its target sites in *C*. *cinctus* and *C*. *radicans*. With the primer positions and the inserts excluded, the alignment of the V4 regions of the 216 sequences in the 18S dataset included 390 positions. All terminal taxa in the 18S FastTree (S2 Fig) were observed also in the V4 tree (S6 Fig), and so were all of the Clades I-VII and their internal topology, with high bootstrap support.

#### The V9 region

The alignment of the V9 region included 125 positions, excluding primer positions and inserts. The region was missing or incomplete in the sequences of *B*. *jadranum*, *C*. *affinis*, *C*. *laevisporus*, *C*. *mannaii*, *C*. *mitra* and *C*. *neogracilis* (cryptic species 2 and 4). In about half of the other chaetocerotacean sequences ca. 22 bases at the 3′-end were missing as well. Inserts corrupted the V9f primer near its non-critical 5′-end in eight taxa (Table 1). The V9f primer target region matched the primer perfectly in all other chaetocerotacean taxa. Inserts were detected in the V9f primer target region of six terminal taxa and in one of the *C*. *decipiens* strains (Table 1). Most of the terminal taxa recovered in the 18S tree were recovered also in the V9 tree (S7 Fig). Yet, several pairs, or even groups, of sister terminal taxa in the 18S tree were found to collapse into single terminal taxa in the V9 tree, for example, the group of *C*. *diadema 1*, *C*. *diadema 2* and *C*. *rotosporus*, and the pair of *C*. *sporotruncatus* and *C*. *dichatoensis*. In the V9 tree *Chaetoceros* was not monophyletic.

## Discussion

We gathered 413 partial 28S and 216 18S sequences from 443 strains of the genera *Chaetoceros* and *Bacteriastrum*. Of these, 244 28S sequences and 164 18S sequences were collected in this study, mainly from the Gulf of Naples but also from the European Atlantic and Chilean Pacific coast. The ingroup sequences grouped in at least 76 terminal taxa of *Chaetoceros* and eight of *Bacteriastrum*, providing a reasonable coverage of the species diversity in these genera, at least for what concerns coastal temperate waters. Many of the isolated strains correspond to described species for which 28S or 18S sequences were already available. Nonetheless, 94 isolated strains correspond morphologically to 25 described species for which no sequences were available yet, 15 strains (four terminal taxa) resemble but do not quite match described species (cf.), and 31 strains (nine terminal taxa) whose morphology apparently does not match at all with any described species. Results of the present study corroborate findings of earlier studies (e.g., [23]; [24]; [25]; [27]; [26]; [28, 29]) showing that at least 17 of the ca. 50 morphospecies are in fact complexes of cryptic and pseudo-cryptic species.

Previous taxonomic studies in this family focused mainly on the 28S to characterize species molecularly and determine their phylogenetic position. The 18S sequences, gathered predominantly in the present study, allowed to compare phylogenies obtained with the two markers demonstrating that trees are remarkably well resolved and agree well, revealing seven major clades. Furthermore, the 18S sequences can act as references for barcoding applications; their V4 and V9 regions are widely used for High Throughput Sequencing metabarcoding to assess biodiversity in planktonic and microbenthic protistan communities (e.g. [31], [10]; [47]). The availability of curated reference sequences produced in this paper represents an important resource for such studies.

### Species circumscriptions

Although several of the species of Chaetocerotaceae that we were able to distinguish genetically using 18S and 28S data cannot be identified reliably using morphological characteristics alone, ultrastructural features of *Bacteriastrum* and *Chaetoceros* do provide potentially useful characters for species delineation and diagnosis. Seta ultrastructure was shown by [48, 49] to be remarkably informative and distinctive for the eleven species of the subgenus *Phaeoceros* and the 22 in the subgenus *Hyalochaetae* they studied. Morphometric analyses of the ultrastructure and density of pores, poroids and spines in the setae need to be carried out on a broader number of taxa to assess the diagnostic value of these characters. Likewise, valve ultrastructure appears be distinctive for some species or groups of related species and is a promising feature to be further explored (see *C*. *brevis*, *C*. *contortus/compressus*, this study, [50]).

Resting spores allow distinctions to be drawn between some closely related species (Ishii et al. 2011). Species within the *C*. *socialis* complex (Clade VI) are basically indistinguishable in their vegetative morphology ([33]; [24]; [26]), but their spore morphology differs markedly. Spores of *C*. *dichatoensis* [26] exhibit valves ornamented with spines whose basal parts form curved ridges; in those of *C*. *sporotruncatus* [26] the primary valve is ornamented on its central portion with raised lenticular-shaped structures; those of *C*. *gelidus* are smooth with more or less fused processes along the valve rim [24]; and those of *C*. *socialis* [24] exhibit spines on both valves, but no ridges. Spore morphology also differs markedly among species in sub-clade VIIb, which share a very similar chain and cell morphology. Spores of the *C*. *diadema* complex ([27]; this study) possess one or several dichotomously branching spines on one of the valves, those of *C*. *rotosporus* [19] are smooth and possess a wing-like structure, and those of *C*. *seiracanthus* possess numerous spines on both valves and one of its valves exhibit a characteristic bulbous protuberance. Similarly, spore morphology can help distinguishing *C*. *protuberans* from its sister *C*. *didymus*.

In a few cases, however, convergence or parallelism in spore morphology has been reported between distantly related species. For example, *C*. *curvisetus*, *C*. *pseudocurvisetus*, *C*. *gelidus* (all in Clade VI), and *C*. *protuberans* (in Clade II) all form smooth spores with a siliceous collar ([27], [24]). In addition, spore morphology can show intraspecific plasticity, e.g. in *C*. *dichatoensis* [26]. Although neither plasticity nor convergence are phylogenetically positively informative, they prompt questions about causation and functionality.

### Cryptic diversity

The present study has added additional cryptic entities to morphologically delineated taxa for which cryptic diversity was already known, which include *C*. *contortus* ([51]; [25]), *C*. *curvisetus*, *C*. *debilis*, and *C*. *peruvianus* [27], *C*. *diadema* [19] and *C*. *lorenzianus–decipiens* [29]. We uncovered cryptic and pseudocryptic diversity in *B*. *furcatum*, *C*. *brevis*, *C*. *diadema*, *C*. *didymus*, *C*. *diversus* and *C*. *tortissimus*. Thus, in Chaetocerotaceae, as in most other diatom lineages, cryptic diversity is common and lineage sorting, as deduced from genetic data, proceeds faster than morphological differentiation [52].

The genetically distinct geographic strains within the morphospecies *C*. *didymus*, *C*. *debilis*, and *C*. *radicans* could represent biologically separate species, but genetic differentiation between distant populations is by itself no proof for them being biologically separate. Reproductive incompatibility would provide such proof but is experimentally difficult to achieve because Chaetocerotaceae are homothallic; strains generate both male and female gametes. Yet, the fact that the cryptic or pseudocryptic entities within some of the morphospecies (e.g. *C*. *curvisetus*, *C*. *diadema*, *C*. *diversus*, and *C*. *tortissimus*) coexist in the Gulf of Naples but retain their genetic identity, suggest that they represent biologically distinct (i.e. reproductively isolated) species.

Even within our limited geographic sample coverage, identical sequences have been obtained from conspecific strains obtained from distant sites in each of the following species: *C*. *constrictus*, *C*. *costatus*, *C*. *diadema*, *C*. *elegans*, *C*. *laevisporus*, *C*. *peruvianus*, *C*. *protuberans*, *C*. *rotosporus* and *C*. *socialis*, suggesting that these species are widely distributed. Studies on genetic structuring among large numbers of strains in other planktonic diatom genera have shown that geographical distribution patterns differ markedly among groups of closely related species. In *Skeletonema*, *S*. *japonicum* appears widely distributed in both northern and southern latitudes and *S*. *tropicum* occurs all over the tropics and temperate zones, whereas e.g. *S*. *grethae* seems to be restricted to the warm-temperate Atlantic coast of the USA [53]. Within *Pseudo-nitzschia pungens*, three cryptic species occur, exhibiting markedly distinct distribution patterns ([54]; [55]).

We refrained from formally assigning species names to any of the cryptic entities in the species complexes and we refer to them as ‘species name’ 1, 2, 3, etc. The provision of reference sequences coupled with the morphological information included in this paper will hopefully foster the study of the chaetocerotacean diversity in different geographic areas also taking advantage of HTS-based environmental metabarcode studies, with the goal of achieving a sounder circumscription of the different cryptic and morphologically distinct species.

### Introns

The long insertions can be identified as Group IC1 introns based on their rRNA primary structural details and the phylogenetic relatedness of their conserved regions with homologous regions of Group IC1 introns in other organisms ([56]; [57]; [58]). The short ones comprise spliceosomal introns ([59]; [60]) based on rRNA sequence details. Spliceosomal introns are generally found in eukaryotic protein-coding genes but have also been detected in the ribosomal genes of Ascomycetes [59], and now also of diatoms ([61]; this study). The two longer inserts at locations #14 and #15 exhibit the typical spliceosomal 5′-end (GTAATA and GTACGT, respectively), but their exact nature is not clear.

The introns uncovered in this study are restricted to Clades I and VII, where they occur in some but not all species. Why they occur only in these clades is not clear, and neither is it clear why the 18S of some species seems to be crowded with introns (e.g. *C*. *diversus* 2 with eleven of them). The introns are less conserved than the rDNA core regions. At times the intron sequences differ between conspecific strains, and in a few cases even show intra-individual polymorphism (presence/absence). Such introns could be used as markers to discriminate seasonal populations within a species or different cryptic species within a morpho-species, simply based on the length of PCR products.

The presence of multiple introns, especially the Group 1 introns, in 18S genes will affect PCR-amplification and sequencing, also because disrupted primer sites render many internal sequencing primers useless. These issues may have frustrated earlier attempts to infer chaetocerotacean phylogenies using this marker because the species in whose 18S the introns are encountered are common and widespread. Since the introns are spliced out during maturation of the rRNA product ([57]; [60]), biodiversity assessments of environmental samples based on rRNA might provide a more complete picture of the chaetocerotacean diversity than those based on rDNA, depending on which marker region in the ribosomal genes and which HTS primers are used for the assessment. However, starting from environmental rRNA for metabarcoding implies addition of a reverse transcription step in the protocol. Moreover, RNA degrades more rapidly in environmental samples than DNA. So, it depends on the aims of the study whether to start from rRNA or rDNA.

### Species detection and identification using the V4 and V9 markers

Regarding the V4 region, virtually all chaetocerotacean species can be differentiated from one another using only this marker. Evidence for differentiation among species is based on the entire 18S, partial 28S, cell- and chain morphology, and frustule ultrastructure. There are only two cases in our dataset in which the V4-core region is unable to distinguish entities for which we have evidence that these entities are genetically and morphologically distinct. The two morphotypes of *C*. *diversus* possess identical 18S-core regions, but they differ in their setae orientation, in their 28S, and in the presence—absence of introns, and in sequence differences in introns present in both morphotypes. They can therefore be told apart. All these differences suggest that the morphotypes constitute distinct species. The two groups of strains of *C*. cf. *vixvisibilis* exhibit identical 18S and 28S core sequences, but their introns differ markedly. The two groups could represent two genetically distinct populations or constitute distinct species. In any case, genetic differentiation exists below what is detectable by 18S and allows for discrimination of such closely related entities.

The V4-primers [32] fit their intended target region in virtually all chaetocerotacean species, allowing detection of almost all of them in HTS metabarcoding. Mismatches between the critical 5′-end of the V4 reverse primer and its target region in *C*. *cinctus* and *C*. *radicans* are likely to affect amplification of their V4 regions, and hence, their detection in HTS metabarcodes. Yet, this needs to be checked with monoclonal cultures or with field samples in which these species are present. Even the six species exhibiting a spliceosomal intron in their V4 region are detectable despite the fact that the region including the insert is close to 600 bp in length. HTS-data polishing procedures generally trash long forward and reverse sequences because they cannot be processed reliably into contigs. However, with appropriate adjustments to the bioinformatics, such sequences can be recuperated and assigned correctly.

Since the tree inferred from chaetocerotacean V4 sequences is reasonably well resolved, groups of HTS metabarcode sequences close to reference sequences are likely to represent relatives of the species. The good resolution is relevant also for the taxonomic characterization of metabarcodes that are not particularly close to reference sequences of known species, as it allows accurate placement of such metabarcodes in or outside Chaetocerotaceae.

Several large datasets have been generated with the V9-region, e.g. TARA [62]; [30]), offering a prime opportunity to assess distribution patterns of chaetocerotacean species at the global scale. Inserts in the V9-region or its primer target regions could affect detection of at least eight chaetocerotacean species and information is still missing for several species in the clade of *C*. *lorenzianus* and its relatives (Clade VIId). Despite the fact that the V9 is shorter than V4, most of the species identified in this study can be discriminated also when using just the V9 as metabarcode marker. Moreover, HTS metabarcodes close to the reference sequences are likely to represent relatives of these species. However, the peculiar sister relationships among ingroup- and outgroup clades in the V9-tree seriously impairs the reliability of placing taxonomically unassigned metabarcodes in or outside Chaetocerotaceae.

## Supporting information

S1 Fig 18S rRNA secondary structure model with intron locations in the DNA sequence. 18S rRNA secondary structure model of *Chaetoceros tenuissimus* strain CHMS01 (http://www.rna.icmb.utexas.edu) with the 19 locations at which introns have been detected in the chaetocerotacean 18S rDNA sequences mapped over it. Note that *C*. *tenuissimus* 18S itself does not contain introns, and neither would any mature ribosome because intron sequences are removed from the maturing rRNA.
(PDF)

S2 Fig FastTree of all 18S sequences included in this study. Figures on the left side of clades are bootstrap values (1000 replicates).
(PDF)

S3 Fig Bayesian Inference tree obtained using MrBayes 3.2.2 with the same 18S sequences as in Fig 1. Figures on the left side of clades are posterior probability values expressed in %. Note that values below 95% signify insufficient support. *Chaetoceros* spp represent species requiring taxonomic description; the first code refers to the representative strain of the Clade as a proxy for the species name, the second code refers to the actual strain.
(TIF)

S4 Fig FastTree of all 28S sequences included in this study. Figures on the left side of clades are bootstrap values (1000 replicates).
(PDF)

S5 Fig Bayesian Inference tree obtained using MrBayes 3.2.2 with the same 28S sequences as in Fig 2. Figures on the left side of clades are posterior probability values expressed in %. Note that values below 95% signify insufficient support. *Chaetoceros* spp represent species requiring taxonomic description; the first code refers to the representative strain of the Clade as a proxy for the species name, the second code refers to the actual strain.
(TIF)

S6 Fig FastTree of available V4 sequences. Figures on the left side of clades are bootstrap values (1000 replicates).
(PDF)

S7 Fig FastTree of available V9 sequences. Figures on the left side of clades are bootstrap values (1000 replicates).
(PDF)

S1 Table Strains included in this study. References are provided for morphological and molecular information of strains not collected in this study. Collection date: dd/mm/yyyy; NA indicates not available. Regarding the V4 and V9 regions: A available; I incomplete, and N not available.
(XLSX)

S2 Table Primers used for PCR-amplification and sequencing. (DOCX)

S3 Table V4-primers and their misfits in primer target sites in the 18S of *Chaetoceros* species. Misfits are indicated in boldface—normal script. Primer target sites are depicted in the forward reading frame.
(DOCX)

S1 Supporting Information Description of the *Bacteriastrum* and *Chaetoceros* taxa included in this study. The criteria for attribution to different types is provided in the Material and Methods section.
(DOCX)

S1 File Figs 1–44 of S1 Supporting Information. Photographic illustrations (LM, SEM, TEM) of cells and frustule elements of selected strains of *Bacteriastrum* and *Chaetoceros* taxa included in this study.
(PDF)
