# Supplementary figures and images for: Annotated 18S and 28S rDNA reference sequences of taxa in the planktonic diatom family Chaetocerotaceae
Source: PLoS One. 2018 Dec 26;13(12):e0208929. doi: 10.1371/journal.pone.0208929 (PMC6306197; doi:10.1371/journal.pone.0208929)

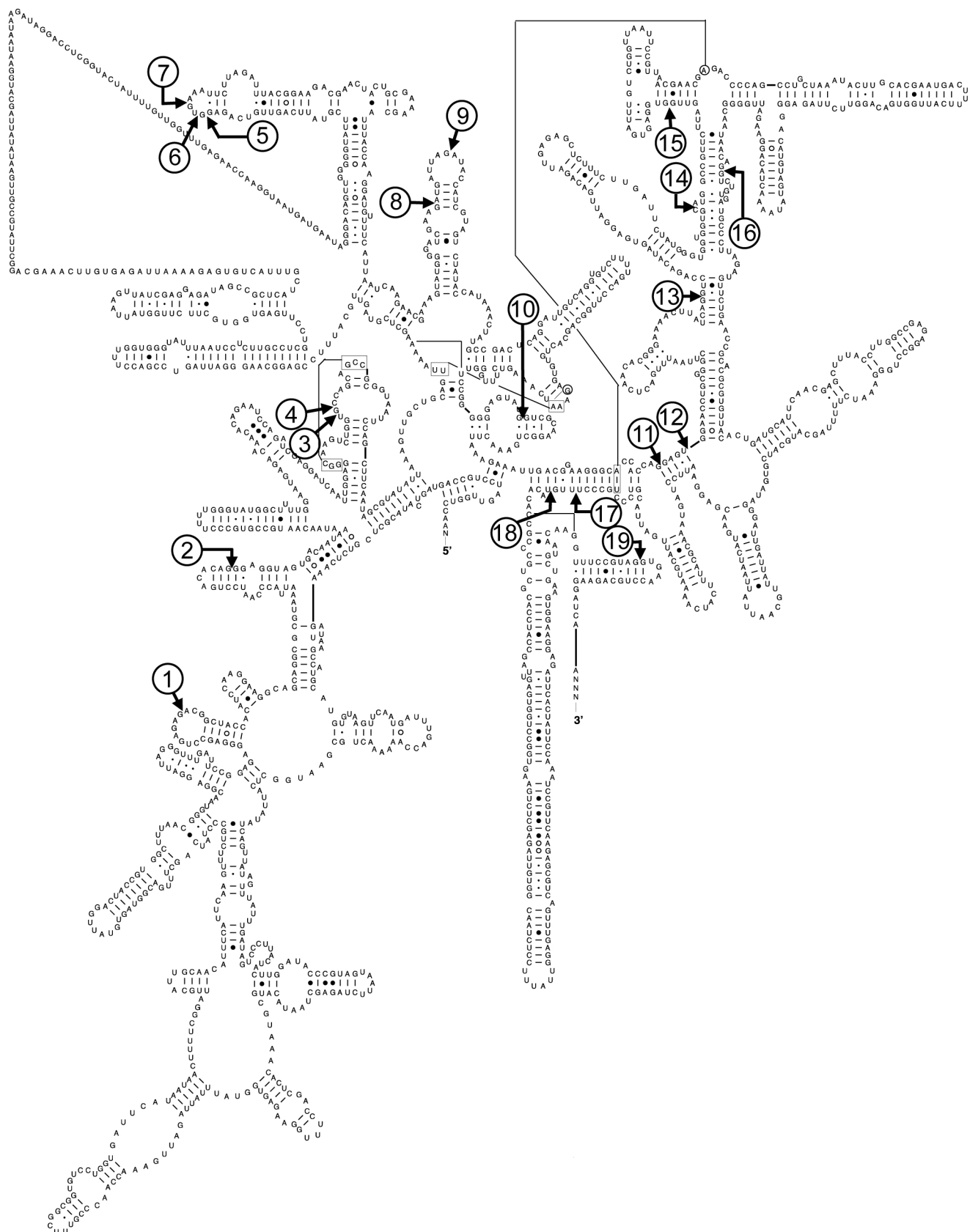

Supplement: S1 Fig — 18S rRNA secondary structure model of Chaetoceros tenuissimus strain CHMS01 (http://www.rna.icmb.utexas.edu) with the 19 locations at which introns have been detected in the chaetocerotacean 18S rDNA sequences mapped over it. Note that C. tenuissimus 18S itself does not contain introns, and neither would any mature ribosome because intron sequences are removed from the maturing rRNA. (PDF) [file pone.0208929.s001.pdf]

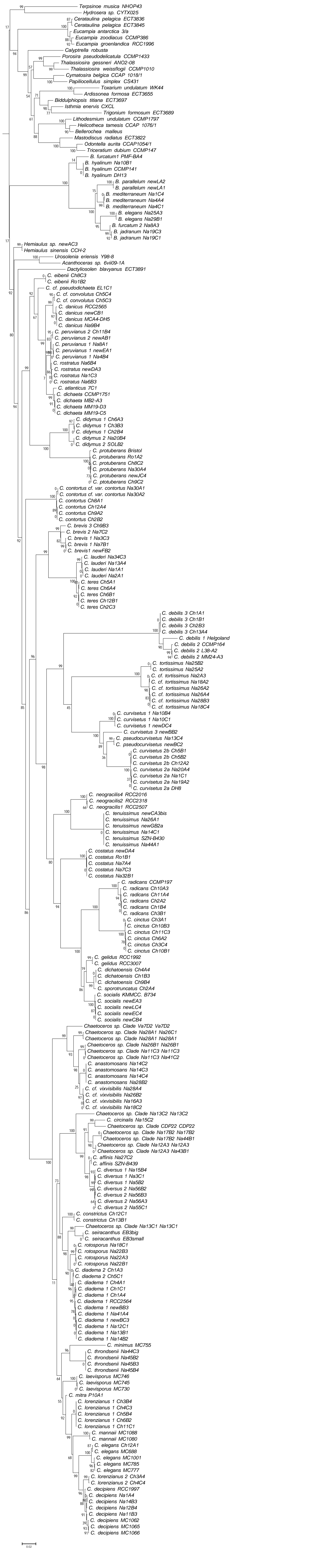

Supplement: S2 Fig — Figures on the left side of clades are bootstrap values (1000 replicates). (PDF) [file pone.0208929.s002.pdf]

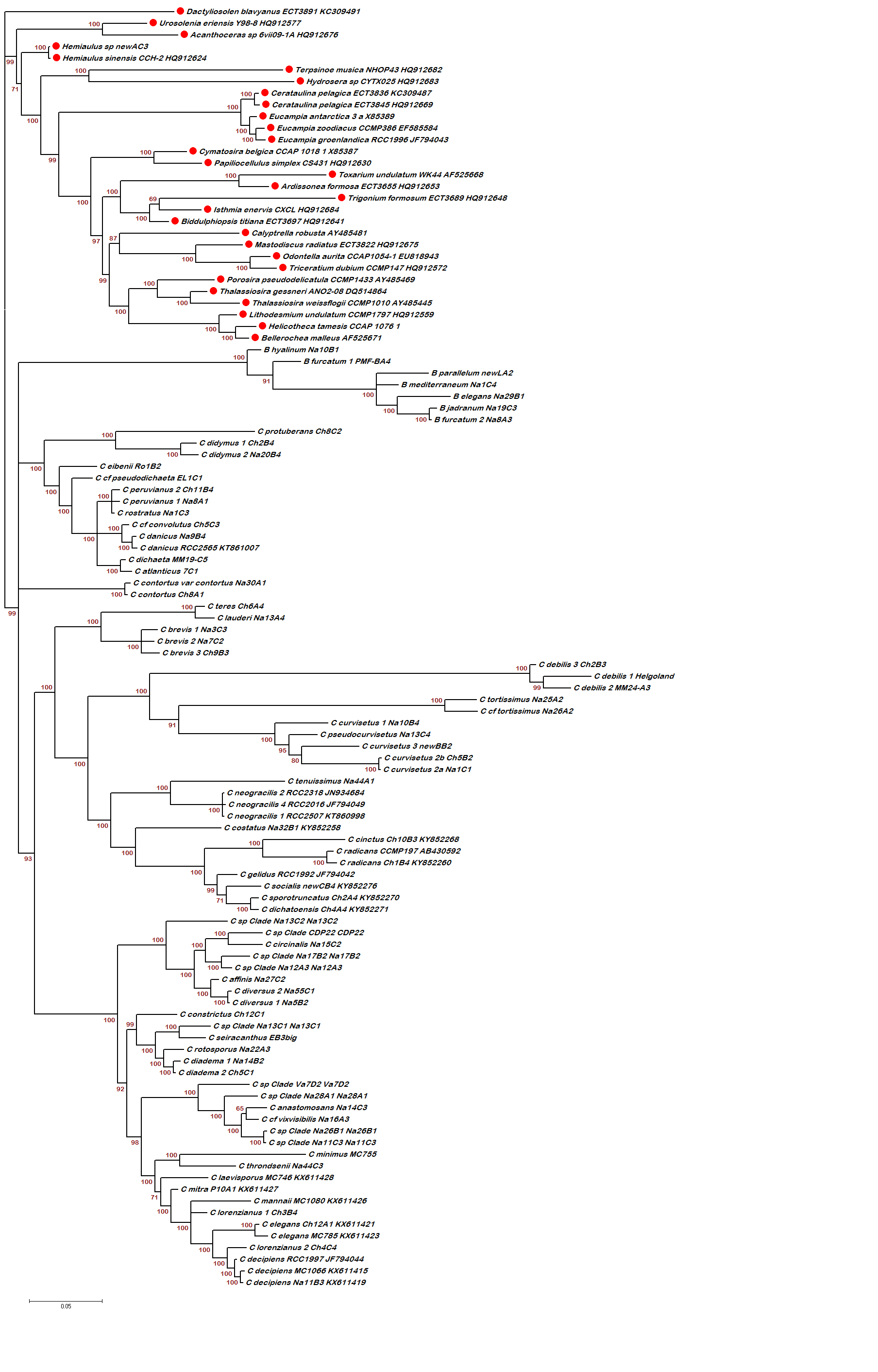

Supplement: S3 Fig — Figures on the left side of clades are posterior probability values expressed in %. Note that values below 95% signify insufficient support. Chaetoceros spp represent species requiring taxonomic description; the first code refers to the representative strain of the Clade as a proxy for the species name, the second code refers to the actual strain. (TIF) [file pone.0208929.s003.tif]

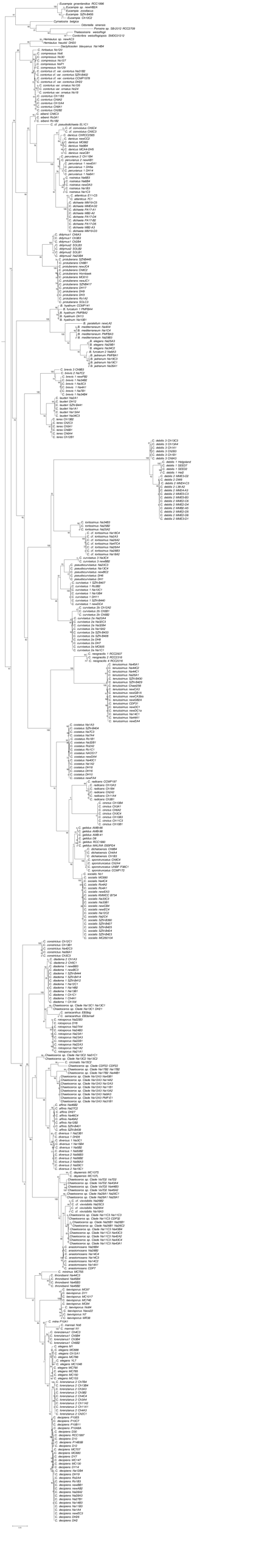

Supplement: S4 Fig — Figures on the left side of clades are bootstrap values (1000 replicates). (PDF) [file pone.0208929.s004.pdf]

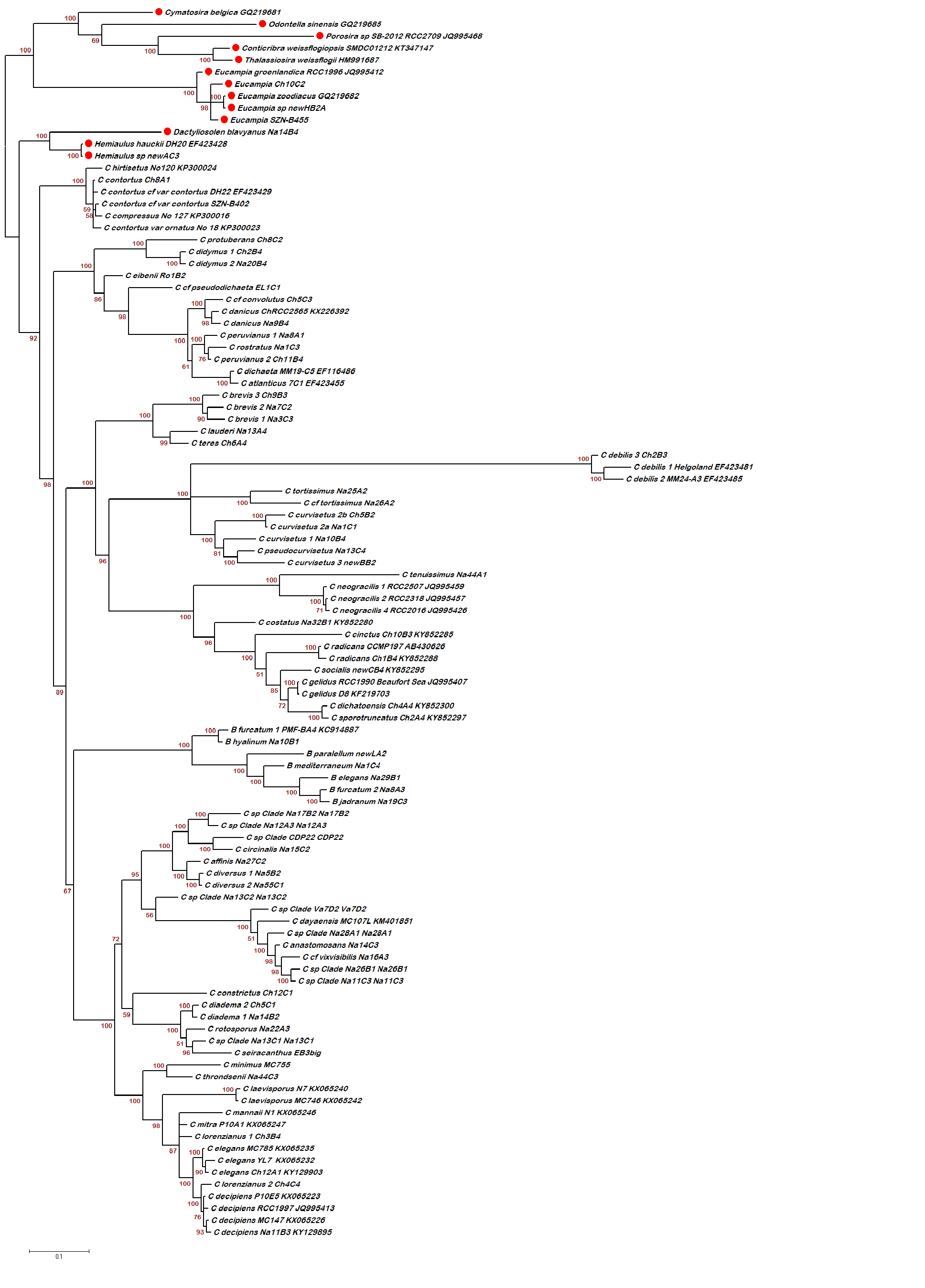

Supplement: S5 Fig — Figures on the left side of clades are posterior probability values expressed in %. Note that values below 95% signify insufficient support. Chaetoceros spp represent species requiring taxonomic description; the first code refers to the representative strain of the Clade as a proxy for the species name, the second code refers to the actual strain. (TIF) [file pone.0208929.s005.tif]

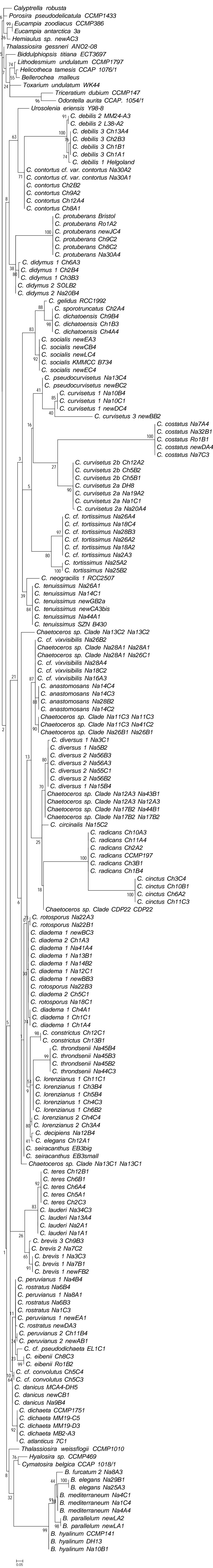

0.05

Supplement: S7 Fig — Figures on the left side of clades are bootstrap values (1000 replicates). (PDF) [file pone.0208929.s007.pdf]
