# Supplementary material for: Annotated 18S and 28S rDNA reference sequences of taxa in the planktonic diatom family Chaetocerotaceae
Source: PLoS One. 2018 Dec 26;13(12):e0208929. doi: 10.1371/journal.pone.0208929 (PMC6306197; doi:10.1371/journal.pone.0208929)
Supplement: S2 Table — (DOCX) [file pone.0208929.s009.docx]

**S2 Table. Primers used for PCR-amplification and sequencing**.

| **Primer** | **Primer** | **Primer** | **Sequence** | **Reference** |
| --- | --- | --- | --- | --- |
| **Name** | **Position** | **direction** | **(5’→3’)** |  |
| **28S rDNA primers for PCR and Sequencing** | | | | |
| D1R | external | Forward | ACCCGCTGAATTTAAGCATA | Scholin *et al.* 1994 |
| D2C2 | Internal | Reverse | CCTTGGTCCGTGTTTCAAGA | Scholin *et al.* 1994 |
| D2R2 | Internal | Reverse | TGAAAAGGACTTTGAAAAGA | Scholin *et al.* 1994 |
| D3Ca | external | Reverse | ACGAACGATTTGCACGTCAG | Scholin *et al.* 1994 |
| **18S rDNA primers for PCR and Sequencing** | | | | |
| SSU-F | external | Forward | TCYAAGGAAGGCAGCAGGCGC | Hamsher *et al*. 2011 |
| SSU-R | external | Reverse | GTTTCAGCCTTGCGACCATACTCC | Ki *et al.* 2007 |
| Ch-11F | external | Forward | TGATCCTGCCAGTAGTCATACGCT | Alverson *et al.* 2007 |
| Ch-82F | internal | Forward | TTGAAACTGCGAAYGGCTCAT | This study |
| Ch-300F | internal | Forward | ATTAGGGTTTGATTCCGGAGAGG | This study |
| Ch-528F | internal | Forward | GCGGTAATTCCAGCTCCAATAGCGT | This study |
| Ch-536R | internal | Reverse | AGCTCCAATTACCGCGGCTGCTGGCA | This study |
| Ch-690F | internal | Forward | TMAGAGGTGAAATTCTTAG | This study |
| Ch-690R | internal | Reverse | TCTAAGAATTTCACCTCTKA | This study |
| Ch-1055R | internal | Reverse | TAAGAACGGCCATGCACCACCACC | This study |
| Ch-1055F | internal | Forward | GTGGTGGTGCATGGCCGTTCTTAG | This study |
| 1147R | internal | Reverse | AGTTTCAGCCTTGCGACCATAC | Alverson *et al.* 2007 |
| Ch-1147F | internal | Forward | GGTCGCAAGGCTGAAACT | This study |
| Ch-1400R | internal | Reverse | ACGGGCGGTGTGTACAAAGGGCA | This study |
| Ch-1400F | internal | Forward | CCTTTGTACACACCGCCCGTCGCA | This study |
| TAR-EukF1 | internal | Forward | CCAGCAGCCGCGGTAATTCC | Stoeck *et al.* 2010 |
| TAR-EukR | internal | Reverse | ACTTTCGTTCTTGATYAATGA | Stoeck *et al.* 2010 |
| Ch-V9R | external | Reverse | CCTTCYGCAGGTTCACCTAC | Amaral-Zettler *et al.* 2009 |

Alverson, A. J., Jansen, R. K., & Theriot, E. C. (2007). Bridging the Rubicon: Phylogenetic analysis reveals repeated colonizations of marine and fresh waters by thalassiosiroid diatoms. Molecular Phylogenetics and Evolution, 45, 193-210.

Amaral-Zettler, L. A., McCliment, E. A., Ducklow, H. W., Huse, S. M. (2009). A method for studying protistan diversity using massively parallel sequencing of V9 hypervariable regions of small-subunit ribosomal RNA genes. PLOS ONE 4(12): 10.1371/annotation/50c43133-0df5-4b8b-8975-8cc37d4f2f26.

Hamsher, S. E., Evans, K. M., Mann, D. G., Poulícková, A., & Saunders, G. W. (2011). Barcoding Diatoms: Exploring Alternatives to COI-5P. Protist, 162(3), 405-422.

Ki, J.-S., & Han, M.-S. (2007). Informative Characteristics of 12 Divergent Domains in Complete Large Subunit rDNA Sequences from the Harmful Dinoflagellate Genus, Alexandrium (Dinophyceae) doi:10.1111/j.1550-7408.2007.00251.x. Journal of Eukaryotic Microbiology, 54(2), 210-219.

Scholin, C. A., Herzog, M., Sogin, M., & Anderson, D. M. (1994). Identification of group- and strain-specific genetic markers from globally distributed Alexandrium (Dinophyceae). II. Sequence analysis of fragments of the LSU rRNA gene. Journal of Phycology, 30, 999-1011.

Stoeck, T., Bass, D., Nebel, M., Christen, R., Jones, M. D. M., Breiner, H.-W., & Richards, T. A. (2010). Multiple marker parallel tag environmental DNA sequencing reveals a highly complex eukaryotic community in marine anoxic water. Molecular Ecology, 19, 21-31. doi: 10.1111/j.1365-294X.2009.04480.x
