# Supplementary material for: Annotated 18S and 28S rDNA reference sequences of taxa in the planktonic diatom family Chaetocerotaceae
Source: PLoS One. 2018 Dec 26;13(12):e0208929. doi: 10.1371/journal.pone.0208929 (PMC6306197; doi:10.1371/journal.pone.0208929)
Supplement: S3 Table — Misfits are indicated in boldface—normal script. Primer target sites are depicted in the forward reading frame. (DOCX) [file pone.0208929.s010.docx]

**S3 Table.** V4-primers and their misfits in primer target sites in the 18S of *Chaetoceros* species. Misfits are marked in red. Primer target sites are depicted in the forward reading frame.

| **V4-Primer**  **Species** | TAR-EukF1 (forward)  CCAGCASCYGCGGTAATTCC | TAR-EukR (reverse)  ACTTTCGTTCTTGATYRATGA |
| --- | --- | --- |
| *C. anastomosans* | CCAGCAGCCGCGGTAATTCC | TCATTG**g**TCAAGAACGAAAGT |
| *C. cinctus* | CCAGCAGCCGCGGTAATTCC | T**a**ATTGATCAAGAACGAAAGT |
| *Chaetoceros sp.* Clade Na11C3 | CCAGCAGCCGCGGTAATTCC | TCATTG**g**TCAAGAACGAAAGT |
| *Chaetoceros sp.* Clade Na26B1 | CCAGCAGCCGCGGTAATTCC | TCATTG**g**TCAAGAACGAAAGT |
| *C.* cf. *vixvisibilis* | CCAGCAGCCGCGGTAATTCC | TCATTG**g**TCAAGAACGAAAGT |
| *C. radicans* | CCAGCAGCCGCGGTAATTCC | T**gt**TTG**g**TCAAGAAC**t**AAAGT |
