# Supplementary material for: Annotated 18S and 28S rDNA reference sequences of taxa in the planktonic diatom family Chaetocerotaceae
Source: PLoS One. 2018 Dec 26;13(12):e0208929. doi: 10.1371/journal.pone.0208929 (PMC6306197; doi:10.1371/journal.pone.0208929)
Supplement: S1 Supporting Information — The criteria for attribution to different types is provided in the Material and Methods section. (DOCX) [file pone.0208929.s011.docx]

Annotated 18S and 28S rDNA reference sequences of taxa in the planktonic diatom family *Chaetocerotaceae*

Chetan C. Gaonkar, Roberta Piredda, Carmen Minucci, David G. Mann, Marina Montresor, Diana Sarno, Wiebe H.C.F. Kooistra

**S1 Supporting Information**

**Description of the *Bacteriastrum* and *Chaetoceros* taxa included in this study; the criteria for attribution to different types is provided in the Material and Methods section.**

**CLADE I**

***B. hyalinum* Lauder (Type A)**

Lauder HS. Remarks on the marine Diatomaceae found at Hong Kong, with descriptions of new species. Trans Micr Soc London, n s. 1864;12:75-9.

Type locality: Hong Kong Harbour, China

The 18S sequences were identical (S2 Fig) whereas the 28S sequences exhibited minor variation and grouped in a grade with *B. furcatum* 1 in the 28S tree (S4 Fig). Spore formation was observed in Strain Na10B1.

Additional references:

Kooistra WHCF, Sarno D, Hernández-Becerril DU, Assmy P, Di Prisco C, Montresor M. Comparative molecular and morphological phylogenetic analyses of taxa in the Chaetocerotaceae (Bacillariophyta). Phycologia. 2010;5:471-500.

Bosak S, Šupraha L, Nanjappa D, Kooistra WHCF, Sarno D. Morphology and phylogeny of four species from the genus *Bacteriastrum* (Bacillariophyta). Phycologia. 2015;54:130–48.

***B. furcatum* Shadbolt (Type C)**

Shadbolt G. A short description of some new forms of Diatomaceae from Port Natal. Trans Micr Soc London, n s. 1854;2:13-8.

Type locality: Port Natal (= Durban), South Africa

The sequences of two morphotypes corresponding to *B. furcatum* were not resolved as sisters in either the 18S and 28S trees. The sequence of the Adriatic strain PMF-BA4 attributed to *B. furcatum* in Bosak et al. (2015) was recovered as sister to a clade with the sequences of *B. hyalinum*, while the sequence of the Neapolitan strain Na8A3 (Fig. 1 in S1 File) was sister to *B. jadranum* (S2 Fig; S4 Fig). We identified the two as *B. furcatum* 1 and *B. furcatum* 2, respectively. The morphology of both strains matched with the Neapolitan strain illustrated in Sarno et al. (1997).

Additional references:

Bosak S, Šupraha L, Nanjappa D, Kooistra WHCF, Sarno D. Morphology and phylogeny of four species from the genus *Bacteriastrum* (Bacillariophyta). Phycologia. 2015;54:130–48.

Sarno D, Zingone A, Marino D. *Bacteriastrum parallelum* sp. nov., a new diatom from the Gulf of Naples, and new observations on *B. furcatum* (Chaetocerotaceae, Bacillariophyta). Phycologia. 1997;36:257-66.

***B. parallelum* Sarno, Zingone & D. Marino (Type B)**

Sarno D, Zingone A, Marino D. *Bacteriastrum parallelum* sp. nov., a new diatom from the Gulf of Naples, and new observations on *B. furcatum* (Chaetocerotaceae, Bacillariophyta). Phycologia. 1997;36:257-66.

Type locality: Gulf of Naples, Italy.

The cell morphology of the strains isolated in this study (Fig. 2 in S1 File) matched the original description. The 18S sequences were identical (S2 Fig). Only one 28S sequence was available (S4 Fig).

***B. mediterraneum* Pavillard (Type A)**

Pavillard J. Recherches sur les Péridiniens du Golfe du Lion. Trav Inst Bot Univ Montpellier. 1916;4:9-70.

Type locality: Gulf of Lyon, France.

The sequences grouped in a single terminal clade in both 18S and 28S trees (S2 Fig; S4 Fig).

Additional reference:

Bosak S, Šupraha L, Nanjappa D, Kooistra WHCF, Sarno D. Morphology and phylogeny of four species from the genus *Bacteriastrum* (Bacillariophyta). Phycologia. 2015;54:130–48.

***B. elegans*** **Pavillard (Type B)**

Pavillard J. Recherches sur les Péridiniens du Golfe du Lion. Trav Inst Bot Univ Montpellier. 1916;4:9-70.

Type locality: Gulf of Lyon, France.

The morphology of the strains isolated from the Gulf of Naples, for which we provide additional information (Figs 3-4 in S1 File), matched the original description. The sequences grouped in a single terminal clade in both 18S and 28S trees (S2 Fig; S4 Fig).

Cells cylindrical, colonial, containing numerous small plastids. Colonies heteropolar, with the setae of the anterior terminal valve being different from those of the posterior one. Valve face and mantle perforated with large poroids; central annulus. Some intercalary valves present a T-shaped outgrowth projecting from the valve margin. Terminal valves more silicified than intercalary ones and with a distinctive furrow on the valve mantle (see Pavillard 1916). Terminal valve with a central, slit-shaped rimoportula. Terminal setae thicker than the intercalary ones. The setae of the anterior terminal valve arise radially and form a regular curve in a counterclockwise direction in valve view. The setae of the posterior terminal valves bend towards the chain and then toward the posterior end with an umbrella shape. Intercalary setae fuse for a small distance with those of the adjacent cells and then diverge in the pervalvar axis. Setae circular in cross-section, ornamented with spirally arranged spines and poroids, and scattered elongated pores. Girdle bands with transverse costae ornamented with scattered poroids. Resting spores not observed.

***B. jadranum* Godrijan, Maric & Pfannkuchen, emend. Bosak & Sarno (Type A)**

Godrijan J, Maric D, Imešek M, Janekovic I, Schweikert M, Pfannkuchen M. Diversity, occurrence, and habitats of the diatom genus *Bacteriastrum* (Bacillariophyta) in the northern Adriatic Sea, with the description of *B. jadranum* sp. nov. Bot Mar. 2012;55:415–26. doi: DOI 10.1515/bot-2011-0021.

Type locality: Adriatic Sea

The sequences grouped in a single terminal clade in both 18S and 28S trees (S2 Fig; S4 Fig). The partial 18S sequence (JF930145) provided in the original description of *B. jadranum* (Godrijan et al. 2012) was found to belong to the Dictyochophyte *Rhizochromulina.*

Additional references:

Bosak S, Pletikapić G, Hozić A, Svetličić V, Sarno D, Viličić D. A novel type of colony formation in marine planktonic diatoms revealed by atomic force microscopy. PLoS ONE. 2012; doi: 10.1371/journal.pone.0044851.

Bosak S, Šupraha L, Nanjappa D, Kooistra WHCF, Sarno D. Morphology and phylogeny of four species from the genus *Bacteriastrum* (Bacillariophyta). Phycologia. 2015;54:130–48.

**CLADE II**

***C. protuberans* Lauder (Type A)**

Lauder HS. Remarks on the marine Diatomaceae found at Hong Kong, with descriptions of new species. Trans Micr Soc London, n s. 1864;12:75-9.

Type locality: Hong Kong, China

The sequences grouped in a single terminal clade in both 18S and 28S trees (S2 Fig; S4 Fig). The morphology of the Neapolitan (Kooistra et al. 2010) and Chilean strains is identical and we provide additional information for the Chilean strain Ch8C2 (Fig. 5 in S1 File).

Additional references:

Kooistra WHCF, Sarno D, Hernández-Becerril DU, Assmy P, Di Prisco C, Montresor M. Comparative molecular and morphological phylogenetic analyses of taxa in the Chaetocerotaceae (Bacillariophyta). Phycologia. 2010;5:471-500.

Lee SD, Joo HM, Lee JH. Critical criteria for identification of the genus *Chaetoceros* (Bacillariophyta) based on setae ultrastructure. II. Subgenus *Hyalochaete*. Phycologia. 2014;53:614-38. doi: 10.2216/14-51r2.1.

***C. didymus* Ehrenberg (Type C)**

Ehrenberg CG. Mitteilung über 2 neue Lager von Gebirgsmassen aus Infusorien als Meeres-Absatz in Nord-Amerika und eine Vergleichung derselben mit den organischen Kreide-Gebilden in Europa und Afrika. Bericht über die zur Bekanntmachung geeigneten Verhandlungen der Königlich Preußischen Akademie der Wissenschaften zu Berlin 1844;1844:57-97.

Type locality: unknown to the authors

From the rDNA trees, the strains from central Chile and those from Naples were genetically distinct but morphologically similar (S2 Fig; S4 Fig). We identified the Chilean strains as *C. didymus* 1 (Fig. 6 in S1 File) and the Neapolitan strains as *C. didymus* 2 (Fig. 7 in S1 File).

Chains long and straight, with wide apertures. Two chloroplasts. The valves possess a characteristic central protuberance. Radially branching costae depart from the central annulus and small poroids perforate the marginal part of the valve face and the mantle. Valves ornamented by several thin and often branched capilli. Terminal valves exhibit a rimoportula located in the center of the protuberance. Intercalary setae arise from valve corners and cross just outside the chain margin. Terminal setae almost straight, U-shaped, and more or less parallel to the colony axis. Setae pentagonal in cross section, ornamented with numerous elliptical poroids aligned on the face of the seta and few large scattered pores. Shark fin-shaped spines aligned on the seta’s corners. Setae rounded in their basal portion, where only large and scattered pores are present. Girdle bands ornamented by transverse costae and relatively large poroids. Resting spores not observed.

It seems that the Chilean strain (Ch2B4) has setae ornamented with stretched elliptical poroids with a lesser number of pores as compared to the Neapolitan strain (Na20B4). The latter has more oval poroids and a higher number of pores.

Additional references:

Lee SD, Joo HM, Lee JH. Critical criteria for identification of the genus *Chaetoceros* (Bacillariophyta) based on setae ultrastructure. II. Subgenus Hyalochaete. Phycologia. 2014;53:614-38. doi: 10.2216/14-51r2.1.

Bosak S, Sarno D. The planktonic diatom genus *Chaetoceros* Ehrenberg (Bacillariophyta) from the Adriatic Sea. Phytotaxa. 2017;314:001–44.

**CLADE III**

***C. eibenii* Grunow in Van Heurck (Type B)**

van Heurck HF. Synopsis des Diatomées de Belgique. Anvers: Edité par l'Auteur; 1885. Texte, 235 p. Atlas, 120 p. p.

Type locality: Borkum, Germany

The morphology of the strains isolated in this study matched the descriptions provided by Lee et al. (2014) and Bosak & Sarno (2017). Here we provide additional information for two French Atlantic strains (Fig. 8 in S1 File). The sequences grouped in a single terminal clade in both 18S and 28S trees (S2 Fig; S4 Fig).

Chains straight with narrow elliptical apertures. Multiple chloroplasts, also in the setae. Valves perforated with poroids and possessing a central annulus with radially branching costae. A central rimoportula, slit-shaped internally and as a short tube externally, present on each valve. Intercalary setae emerge just inside the valve corners and cross on chain margin; sibling setae diverge from the apical plane at an angle of about 45° and bend towards the terminal portions of the chain. Terminal setae oriented parallel to the chain axis, forming a broad V-shape. Setae quadrangular/ pentagonal in cross-section. Seta faces ornamented with very small poroids organized into a grid pattern, matching the description by Lee et al. (2014) and Bosak & Sarno (2017). Girdle bands ornamented with transverse costae alternating with rows of very small poroids and scattered larger pores. Resting spores not observed.

Additional references:

Lee SD, Park JS, Yun SM, Lee JH. Critical criteria for identification of the genus *Chaetoceros* (Bacillariophyta) based on setae ultrastructure. I. Subgenus *Chaetoceros*. Phycologia. 2014;53(2):174-87. doi: 10.2216/13-154.1.

Bosak S, Sarno D. The planktonic diatom genus *Chaetoceros* Ehrenberg (Bacillariophyta) from the Adriatic Sea. Phytotaxa. 2017;314:001–44.

***C*. cf. *pseudodichaeta* Ikari (Type D)**

Ikari J. On some *Chaetoceras* of Japan. I. Botanical Magazine of Tokyo. 1926;40(478):517-34.

Type locality: Seto Sea, Japan

We attributed strain EL1C1, for which both 18S and 28S sequences were obtained (S2 Fig; S4 Fig), to *C.* cf. *pseudodichaeta* because we did not observe the distinctive ultrastructure of the setae (Fig. 9 in S1 File).

Short and straight chains with wide rectangular apertures. Numerous chloroplasts present inside the setae. The setae originate inside the valve margin, have a prominent basal part and cross at the chain margin. Also terminal setae have a basal part and then diverge with a narrow V-shape. A tube-like process is present only on terminal valves.

Additional reference:

Bosak S, Sarno D. The planktonic diatom genus *Chaetoceros* Ehrenberg (Bacillariophyta) from the Adriatic Sea. Phytotaxa. 2017;314:001–44.

***C. peruvianus* Brightwell (Type C)**

Brightwell T. On the filamentous long-horned Diatomaceae, with a description of two new species. Quarterly Journal of Microscopical Science. 1856;4:105-9.

Type Locality: Callao, Peru.

In both the 18S and 28S trees, *C. peruvianus* strains formed two groups (S2 Fig; S4 Fig). 28S sequence of the Neapolitan strain Na8A1, representative of *C. peruvianus* 1, was identical to that of strain DH14 illustrated in Kooistra et al. (2010). We illustrate strain Ch11B4, representative of *C. peruvianus* 2, which also includes strains from the Gulf of Naples (Fig. 10 in S1 File).

Additional references:

Kooistra WHCF, Sarno D, Hernández-Becerril DU, Assmy P, Di Prisco C, Montresor M. Comparative molecular and morphological phylogenetic analyses of taxa in the Chaetocerotaceae (Bacillariophyta). Phycologia. 2010;5:471-500.

Lee SD, Park JS, Yun SM, Lee JH. Critical criteria for identification of the genus *Chaetoceros* (Bacillariophyta) based on setae ultrastructure. I. Subgenus *Chaetoceros*. Phycologia. 2014;53(2):174-87. doi: 10.2216/13-154.1.

***C. rostratus* Ralfs in Lauder (Type B)**

Lauder HS. Remarks on the marine Diatomaceae found at Hong Kong, with descriptions of new species. Trans Micr Soc London, n s. 1864;12:75-9.

Type Locality: Hong Kong, China

The morphology of the strains isolated in this study matched the descriptions provided by Lee et al. (2014) and Bosak & Sarno (2017). The sequences grouped in a single terminal clade in both 18S and 28S trees (S2 Fig; S4 Fig). We provide additional information on strains isolated from the Gulf of Naples (Fig. 11 in S1 File).

Colony straight and robust, with narrow apertures. Solitary cells often seen in culture. Cells not joined by the setae but by the fusion of the central process present on the valve face. The setae arise from valve corners, then diverge perpendicularly to the chain axis, forming variable angles in the apical plane. Numerous small chloroplasts spread in the cell as well as in setae. Valve face ornamented with several poroids and costae radiating from an eccentric annulus. The rimoportula is located within the annulus, close to the central protuberance. Terminal setae have the same orientation as the intercalary setae. Setae ultrastructure match the description of *C. rostratus* provided by Lee et al. (2014) and Bosak & Sarno (2017). Girdle bands ornamented with transverse ribs and large scattered pores. Resting spores not observed.

Additional references:

Lee SD, Park JS, Yun SM, Lee JH. Critical criteria for identification of the genus *Chaetoceros* (Bacillariophyta) based on setae ultrastructure. I. Subgenus *Chaetoceros*. Phycologia. 2014;53(2):174-87. doi: 10.2216/13-154.1.

Bosak S, Sarno D. The planktonic diatom genus *Chaetoceros* Ehrenberg (Bacillariophyta) from the Adriatic Sea. Phytotaxa. 2017;314:001–44.

***C.* cf. *convolutus* Castracane (Type D)**

For *C. convolutus*: Castracane F. Report on the Diatomaceae collected by H.M.S. Challenger during the years 1873-1876. Botany 2(4) I-III. Report on the scientific results of the voyage of HMS Challenger during the years 1873-76. London: Pls. Her Majesty's Stationery Office; 1886. p. 1-178.

Type locality: unknown to the authors.

We attributed the two Chilean strains illustrated here (Fig. 12 in S1 File) to *C.* cf. *convolutus* because we could not see the prehensors between adjacent setae described by Fryxell & Medlin (1981). In both 18S and 28S trees the two strains group in a well-supported clade (S2 Fig; S4 Fig).

Short chains, sometimes twisted, with extremely narrow apertures. Cells heterovalvate due to the emergence pattern of the setae: they emerge from a round prominence in the anterior valve and from a depression in the posterior one. Setae arise inside the valve margin, cross on chain axis and then bend towards one end of the colony. Several globular chloroplasts extended into setae. Valves face ornamented with several poroids. Each valve with a central rimoportula with the shape of a small tube externally. Setae square in cross section with thick arrowhead-shaped spines on the setae edges. Resting spore not observed. The distinction between *C. convolutus* and *C. concavicornis* is not clear.

Additional references:

Fryxell GA, Medlin LK. Chain forming diatoms, evidence of parallel evolution in *Chaetoceros*. Cryptogamie Algol. 1981;2:3-29.

Shevchenko OG, Orlova TY, Hernández-Becerril DU. The genus *Chaetoceros* (Bacillariophyta) from Peter the Great Bay, Sea of Japan. Bot Mar. 2006;49:236-58.

***C. danicus* Cleve (Type A)**

Cleve PT. Pelagisk Diatomeer från Kattegat. In: Petersen CGJ, editor. Det Videnskabelige Udbytte af Kanonbaaden "Hauchs" Togter i de Danske Have Indefor Skagen, I Aarene 1883-86: Kjøbenhavn: Andr. Fred. Høst & Sons Forlag.; 1889. p. 53-6.

Type locality: Kattegat.

The sequences grouped in a single terminal clade in both 18S and 28S trees (S2 Fig; S4 Fig).

Additional references:

Kooistra WHCF, Sarno D, Hernández-Becerril DU, Assmy P, Di Prisco C, Montresor M. Comparative molecular and morphological phylogenetic analyses of taxa in the Chaetocerotaceae (Bacillariophyta). Phycologia. 2010;5:471-500.

Bosak S, Sarno D. The planktonic diatom genus *Chaetoceros* Ehrenberg (Bacillariophyta) from the Adriatic Sea. Phytotaxa. 2017;314:001–44.

***C. atlanticus* Cleve (Type A)**

Cleve PT. On diatoms from the Arctic Sea. Bihang Till Konglica Svenska Vetenskaps- Akademiens Handlingar. 1873;1(13):1-28.

Type locality: Davis Strait, North Atlantic.

The sequences grouped in a single terminal clade in the 28S tree (S4 Fig); we added the 18S sequence (S2 Fig) for the strain 7C1 illustrated in Kooistra et al. (2010) where the 28S was reported.

Additional reference:

Kooistra WHCF, Sarno D, Hernández-Becerril DU, Assmy P, Di Prisco C, Montresor M. Comparative molecular and morphological phylogenetic analyses of taxa in the Chaetocerotaceae (Bacillariophyta). Phycologia. 2010;5:471-500.

***C. dichaeta* Ehrenberg (Type A)**

Ehrenberg CG. Vorläufige Resultate seiner Untersuchungen der ihm von der Südpolreise des Capitain Ross, so wie von den Herren Schayer und Darwin zugekommenen Materialien über das Verhalten des kleinsten Lebens in den Ozeanen und den grössten bisher zugänglichen Tiefen des Weltmeers vor. Bericht über die zur Bekanntmachung geeigneten Verhandlungen der Königlichen Akademie der Wissenschaften zu Berlin. 1844:182-207.

Type Locality: Southern Ocean, Atlantic sector

The sequences grouped in a single terminal clade in both 18S and 28S trees (S2 Fig, S4 Fig).

Additional references:

Kooistra WHCF, Sarno D, Hernández-Becerril DU, Assmy P, Di Prisco C, Montresor M. Comparative molecular and morphological phylogenetic analyses of taxa in the Chaetocerotaceae (Bacillariophyta). Phycologia. 2010;5:471-500.

Assmy P, Hernández-Becerril DU, Montresor M. Morphological variability and life cycle traits of the type species of the diatom genus *Chaetoceros, C. dichaeta*. J Phycol. 2008;44:152-63.

**CLADE IV**

The 28S tree revealed six clades in the *C. contortus/compressus* complex. Five of these corresponded to those described in Chamnansinp et al. (2015), i.e., *C. hirtisetus*, *C. compressus*, *C. contortus* var. *ornatus* and two clades both identified as *C. contortus* cf. var. *contortus.* We have added a sixth clade of *C. contortus,* which includes strains isolated from Chile (S2 Fig; S4 Fig).

***C. contortus* F. Schütt (Type C)**

Schütt F. Arten von Chaetoceras und Peragallia. Ein Beitrag zur Hochseeflora. Berichte der Deutsche Botanisch Gesellschaft. 1895;13:35-50.

Type locality: Baltic Sea

The Neapolitan strains illustrated in Kooistra et al. (2010) and for which only 28S sequences are available, clustered in the two *C. contortus* cf. var. *contortus* identified by Chamnansinp et al. (2015); DH22 grouped with CCMP 1578 in one clade, while SZN-B402 and SZN-B403 clustered in the other one. In the present study, we have isolated two new *C. contortus* strains from the Gulf of Naples. Unfortunately, we could only obtain 18S and thus could not assess the match with the 28S clades illustrated by Chamnansinp et al. (2015) (S2 Fig; S4 Fig). We tentatively attributed these strains to *C. contortus* cf. var. *contortus*, based on their geographic origin. Five more *C. contortus* strains, for which we could obtain both phylogenetic markers (S2 Fig; S4 Fig), were isolated from Chile and for them we provide morphological information (Fig. 13 in S1 File). In the *C. contortus* species complex, there is an additional clade for which only LSU sequences are available: *C. contortus* var. *ornatus* (Chamnansinp et al. 2015)*.*

Chains slightly twisted with hexagonal apertures. Multiple chloroplasts. Valve face broadly elliptical to circular, with radially branched costae departing from the central annulus. Adjacent costae joined by transverse connections; at times small spirals are present at one end of the costae. Terminal valve with central slit-shaped rimoportula with a flattened external tube. Setae arise on the valve face well inside the valve margin and cross at the chain margin. Two types of intercalary setae are present: delicate ones, circular in cross section, ornamented with spirally arranged arrowhead-like spines and S-shaped slits, and robust ones, lacking poroids and ornamented with spirally arranged spines. The robust intercalary setae were observed only in field samples; not in culture material. Terminal setae ultrastructurally similar to the delicate intercalary setae. Girdle bands ornamented with transverse costae and minute poroids. Resting spores not observed.

Additional references:

Chamnansinp A, Moestrup Ø, Lundholm N. Diversity of the marine diatom *Chaetoceros* (Bacillariophyceae) in Thai waters – revisiting *Chaetoceros compressus* and *Chaetoceros contortus*. Phycologia. 2015;54(2):161–75.

Kooistra WHCF, Sarno D, Hernández-Becerril DU, Assmy P, Di Prisco C, Montresor M. Comparative molecular and morphological phylogenetic analyses of taxa in the Chaetocerotaceae (Bacillariophyta). Phycologia. 2010;5:471-500.

**CLADE V**

***C. brevis* Schütt (Type C)**

Schütt F. Arten von Chaetoceras und Peragallia. Ein Beitrag zur Hochseeflora. Berichte der Deutsche Botanisch Gesellschaft. 1895;13:35-50.

Type locality: Atlantic Ocean

Sequences belonging to strains identified as *C. brevis* grouped into three clades in both 18S and 28S trees (S2 Fig; S4 Fig). *C. brevis* 1 (Fig. 14 in S1 File) and *C. brevis* 2 (Fig. 15 in S1 File) included sequences from the Gulf of Naples and *C. brevis* 3 a sequence from Chile (Fig. 16 in S1 File). Strains of the three clades exhibit a similar morphology.

Straight chains with peanut-shaped apertures. Single chloroplast. Valves face with an eccentric annulus, with radially branched costae. The valves have a central, more silicified, area that looks like a gray halo in TEM. Terminal valve with an eccentric rimoportula that is slit-shaped internally and has a flattened tube externally. Intercalary setae arise at the valve margin where they cross. Within the setae, small globules without chlorophyll are present. Setae circular in cross section, ornamented with spiraling spines and rows of minute poroids. Girdle bands ornamented with transverse costae and minute poroids. Resting spores were obtained for *C. brevis* 2 and *C. brevis* 3. Both are biconvex and ornamented on both valves with spines, which in *C. brevis* 3 have a terminal portion resembling ‘capilli’.

Additional reference:

Bosak S, Sarno D. The planktonic diatom genus *Chaetoceros* Ehrenberg (Bacillariophyta) from the Adriatic Sea. Phytotaxa. 2017;314:001–44.

***C. teres* Cleve in Aurivillius (Type B)**

Aurivillius CWS. Das Plankton des Baltischen Meeres von Carl W.S.A.- unter Mitwirkung von P.T. Cleve: Mit einer Tafel und einer Karte. Eingereicht 11 December 18951896.

Type locality: Sweden

The morphology of the strains isolated in this study (Fig. 17 in S1 File) matched the descriptions provided by Hernández-Becerril & Flores Granados (1998) and Shevchenko et al. (2006). The sequences grouped in a single terminal clade in both 18S and 28S trees (S2 Fig; S4 Fig).

Short chains with narrow, lanceolate apertures. Multiple chloroplasts. Long intercalary setae emerging from the valve corner and crossing on the chain margin. All setae oriented in the apical plane. Terminal setae generally U-shaped. Valves with radially branched costae emerging from a large central annulus. Terminal valve with a central rimoportula, slit-shaped internally and with a short, flattened tube externally. Setae circular in cross section, ornamented with a row of spirally arranged spines and multiple rows of spirally arranged poroids. Girdle bands ornamented with transverse costae and small, scattered poroids. Resting spore not observed.

Additional references:

Hernández-Becerril DU, Flores Granados C. Species of the diatom genus *Chaetoceros* (Bacilariophyceae) in the plankton from the southern Gulf of Mexico. Bot Mar. 1998;41:505-19.

Shevchenko OG, Orlova TY, Hernández-Becerril DU. The genus *Chaetoceros* (Bacillariophyta) from Peter the Great Bay, Sea of Japan. Bot Mar. 2006;49:236-58.

***C. lauderi* Ralfs in Lauder (Type A)**

Lauder HS. Remarks on the marine Diatomaceae found at Hong Kong, with descriptions of new species. Trans Micr Soc London, n s. 1864;12:75-9.

Type locality: Hong Kong, China

The sequences grouped in a single terminal clade in both 18S and 28S trees (S2 Fig; S4 Fig).

Additional references:

Bosak S, Sarno D. The planktonic diatom genus *Chaetoceros* Ehrenberg (Bacillariophyta) from the Adriatic Sea. Phytotaxa. 2017;314:001–44.

Kooistra WHCF, Sarno D, Hernández-Becerril DU, Assmy P, Di Prisco C, Montresor M. Comparative molecular and morphological phylogenetic analyses of taxa in the Chaetocerotaceae (Bacillariophyta). Phycologia. 2010;5:471-500.

**CLADE VI**

***C. debilis* Cleve (Type C)**

Cleve PT. Redogörelse för de svenska hydrografiska undersökningarne Åren 1893-1894. II. Planktonundersökningar, Cilicoflagellater och Diatomacéer. Bihang till Kongliga Svenska Vetenskaps-Akademiens Handlingar. 1894;20(Afd. III, 2): 16 pp., 2 pls.

Type locality: Sweden

Sequences belonging to strains identified as *C. debilis* grouped into three clades in both 18S and 28S trees (S2 Fig; S4 Fig). *C. debilis* 1 and *C. debilis* 2 included strains from Helgoland (North Sea) and from the Atlantic sector of the Southern Ocean, respectively (Kooistra et al. 2010). Six strains isolated in this study from Chilean waters formed a third clade, *C. debilis* 3. Strains of the three clades show similar morphology. Here we provide a description of *C. debilis* 3 (Fig. 18 in S1 File).

Spiral chains with hexagonal apertures. Single chloroplast. Setae emerge from valve corners and cross at the chain margin, bent towards the convex side of the colony. Valves with radially branching costae departing from an eccentric annulus. Terminal valve with an eccentric slit-shaped rimoportula that has a flattened tube externally. Setae circular in cross section, ornamented with a row of spirally arranged spines, multiple rows of spirally arranged poroids and solitary large pores. Resting spores were not observed.

Additional reference:

Kooistra WHCF, Sarno D, Hernández-Becerril DU, Assmy P, Di Prisco C, Montresor M. Comparative molecular and morphological phylogenetic analyses of taxa in the Chaetocerotaceae (Bacillariophyta). Phycologia. 2010;5:471-500.

***C. tortissimus* Gran (Type B)**

Gran HH. Bemerkungen über einige Planktondiatomeen. Nytt Magasin for Naturvidenskapene. 1900;38:102-28.

Type locality: Coast and Fjords of Norway.

The morphology of the strains isolated in this study (Fig. 19 in S1 File) matched the descriptions provided by Bosak & Sarno (2017). The sequences grouped in a single terminal clade in both 18S and 28S trees (S2 Fig; S4 Fig).

Long chains strongly twisted around the axis; short chains (4-5 cells) do not show torsion. Narrow hexagonal apertures. One chloroplast. Intercalary setae originate from valve corners and cross over at the chain margin. Terminal setae U-shaped and extending almost parallel to the colony axis. Valves face with a central or slightly eccentric annulus and dichotomously branching costae. Terminal valves with a wide, slit-shaped rimoportula. Setae circular in cross section with a row of spirally arranged small spines and multiple rows of spirally arranged poroids. A large elongated pore is present on the proximal part of the setae. Girdle bands ornamented with transverse costae and scattered minute poroids. Resting spores not observed.

Additional reference:

Bosak S, Sarno D. The planktonic diatom genus *Chaetoceros* Ehrenberg (Bacillariophyta) from the Adriatic Sea. Phytotaxa. 2017;314:001–44.

***C.* cf*. tortissimus* Gran (Type D)**

Both the 18S and 28S trees showed a number of Neapolitan strains that were similar to *C. tortissimus* but formed a sister clade to it (S2 Fig; S4 Fig). We identified these strains as *C.* cf*. tortissimus* (Fig. 20 in S1 File)*.*

As compared to the strains identified as *C. tortissimus,* these strains exhibit larger apertures and chains seem to exhibit a less pronounced torsion, although this feature exhibits marked variation. The ultrastructural features match those of *C. tortissimus*. Resting spores not observed.

***C.* *curvisetus* Cleve (Type C)**

Cleve PT. Pelagisk Diatomeer från Kattegat. In: Petersen CGJ, editor. Det Videnskabelige Udbytte af Kanonbaaden "Hauchs" Togter i de Danske Have Indefor Skagen, I Aarene 1883-86: Kjøbenhavn: Andr. Fred. Høst & Sons Forlag.; 1889. p. 53-6.

Type locality: Kattegat, Sweden.

In both trees, three clades were recorded, grouping sequences of strains identified as *C. curvisetus* and one clade grouping strains of *C. pseudocurvisetus* (S2 Fig; S4 Fig). All four clades clustered within a larger well-supported clade. *C. curvisetus* 1 and *C. curvisetus* 3 include strains isolated from the Gulf of Naples, while *C. curvisetus* 2 includes both Neapolitan and Chilean strains. No differences were detected in gross morphology and cell ultrastructure of strains belonging to the three clades of *C. curvisetus*. *Chaetocero curvisetus* 1 and 2 were illustrated in Kooistra et al. (2010). Here we provide 18S of the three clades and a description of *C. curvisetus* 3 (Fig. 21 in S1 File).

Curved chains with large apertures, elliptical or circular. One chloroplast. Setae bent towards the convex side of the colony. Intercalary setae arise from the valve corners and cross at the chain margin. Terminal valve with a slightly eccentric, slit-shaped rimoportula with a flattened tube externally. Setae ornamented with spirally-arranged spines, spiral rows of poroids interspersed with larger pores. Spores not observed.

Additional reference:

Kooistra WHCF, Sarno D, Hernández-Becerril DU, Assmy P, Di Prisco C, Montresor M. Comparative molecular and morphological phylogenetic analyses of taxa in the Chaetocerotaceae (Bacillariophyta). Phycologia. 2010;5:471-500.

***C. pseudocurvisetus* Mangin (Type A**)

Mangin ML. Sur quelques Algues nouvelles ou peu connues du Phytoplancton de l'Atlantique. Bulletin de la Société Botanique de France. 1910;57(5):344-50. doi: 10.1080/00378941.1910.10832226.

Type locality: Atlantic Ocean

The sequences grouped in a single terminal clade in both 18S and 28S trees (S2 Fig; S4 Fig). 28S sequences and morphology of the strains isolated in this study were identical to those reported in Kooistra et al. (2010); here we provide additional information on the ultrastructure of vegetative cells and resting spores (Fig. 22 in S1 File).

Cells united to form curved chains, with an elliptical or circular aperture. Setae bend towards the convex side of the colony. Single chloroplast. Intercalary setae emerge from the valve apices and cross at the chain margin. Each valve possesses four sub-marginal projections close to the valve margins that join with those on adjacent cells, thus revealing narrow apertures. Valve face ornamented with radial costae branching from the eccentric annulus. Terminal valve with an eccentric, slit-shaped rimoportula. Setae ornamented with a row of spirally-arranged spines and rows of minute poroids. Large pores scattered along the setae. Girdle band with transverse costae. Resting spores smooth and rounded, with both valves possessing a silica collar of variable size, which can be perforated with holes or fissures.

Additional reference:

Kooistra WHCF, Sarno D, Hernández-Becerril DU, Assmy P, Di Prisco C, Montresor M. Comparative molecular and morphological phylogenetic analyses of taxa in the Chaetocerotaceae (Bacillariophyta). Phycologia. 2010;5:471-500.

***C. tenuissimus* Meunier (Type A)**

Meunier A. Microplankton de la mer Flamande. Bruxelles: Hayez, Imprimeur de l'Académie royale de Belgique; 1913.

Type locality: Ostende, Belgium

The sequences grouped in a single terminal clade in both 18S and 28S trees (S2 Fig; S4 Fig). *C. tenuissimus* is generally considered a single-celled species, however, we have also found relatively long colonies (Fig. 23 in S1 File).

Additional reference:

Kooistra WHCF, Sarno D, Hernández-Becerril DU, Assmy P, Di Prisco C, Montresor M. Comparative molecular and morphological phylogenetic analyses of taxa in the Chaetocerotaceae (Bacillariophyta). Phycologia. 2010;5:471-500.

***C. neogracilis* (Schütt) Van Landingham (Type A*)**

Schütt F. Arten von Chaetoceras und Peragallia. Ein Beitrag zur Hochseeflora. Berichte der Deutsche Botanisch Gesellschaft. 1895;13:35-50.

Type locality: Baltic Sea.

18S and 28S sequences of clades I, II and IV from Balzano et al. (2017) were used. Sequences of clade III were not included because only 28S was available. The sequences formed a clade in both 18S and 28S trees (S2 Fig; S4 Fig).

Additional reference:

Balzano S, Percopo I, Siano R, Gourvil P, Chanoine M, Marie D, et al. Morphological and genetic diversity of Beaufort Sea diatoms with high contributions from the *Chaetoceros neogracilis* species complex. J Phycol. 2017;53(1):161-87. doi: doi:10.1111/jpy.12489.

***C. costatus* Pavillard (Type A)**

Pavillard J. Observations sur les Diatomées. Bulletin de la Société Botanique de France. 1911;58:21-9.

Type locality: Mediterranean Sea.

The sequences grouped in a single terminal clade in both 18S and 28S trees (S2 Fig; S4 Fig).

Additional references:

Kooistra WHCF, Sarno D, Hernández-Becerril DU, Assmy P, Di Prisco C, Montresor M. Comparative molecular and morphological phylogenetic analyses of taxa in the Chaetocerotaceae (Bacillariophyta). Phycologia. 2010;5:471-500.

Bosak S, Sarno D. The planktonic diatom genus *Chaetoceros* Ehrenberg (Bacillariophyta) from the Adriatic Sea. Phytotaxa. 2017;314:001–44.

***C. radicans*** Schütt **(Type A*)**

Schütt F. Arten von Chaetoceras und Peragallia. Ein Beitrag zur Hochseeflora. Berichte der Deutsche Botanisch Gesellschaft. 1895;13:35-50.

Type locality: Atlantic Ocean

The strains included in this study are those reported in Gaonkar et al. (2017); in both 18S and 28S trees, the North Sea strain CCMP197 (*C. radicans* 1) is outside the clade grouping the other strains (*C. radicans* 2) (S2 Fig; S4 Fig).

Additional reference:

Gaonkar CC, Kooistra WHCF, Lange CB, Montresor M, Sarno D. Two new species in the *Chaetoceros socialis* complex (Bacillariophyta): *C. sporotruncatus* and *C. dichatoensis*, and characterization of its relatives, *C. radicans* and *C. cinctus*. J Phycol. 2017;53(4):889-907. doi: 10.1111/jpy.12554.

***C. cinctus* Gran (Type A*)**

Gran HH. Protophyta: Diatomaceae, Silicoflagellata og Cilioflagellata. Norske Nordhans-Expedition 1876-1878, XXIV Botanik Christiania1897.

Type locality: Atlantic and North Sea

The strains included in this study are those reported in Gaonkar et al. (2017); the sequences grouped in a single terminal clade in both 18S and 28S trees (S2 Fig; S4 Fig).

Additional reference:

Gaonkar CC, Kooistra WHCF, Lange CB, Montresor M, Sarno D. Two new species in the *Chaetoceros socialis* complex (Bacillariophyta): *C. sporotruncatus* and *C. dichatoensis*, and characterization of its relatives, *C. radicans* and *C. cinctus*. J Phycol. 2017;53(4):889-907. doi: 10.1111/jpy.12554.

***C. socialis* Lauder (Type A*)**

Chamnansinp A, Li Y, Lundholm N, Moestrup Ø. Global diversity of two widespread, colony-forming diatoms of the marine plankton, *Chaetoceros socialis* (syn. *C. radians*) and *Chaetoceros gelidus* sp. nov. J Phycol. 2013;49:1128–41. doi: 10.1111/jpy.12121.

Epitype locality: Daya Bay, Guangdon Coast, South China Sea

The strains included in this study are those reported in Kooistra et al. (2010) and Gaonkar et al. (2017); the sequences grouped in a single terminal clade in both 18S and 28S trees (S2 Fig; S4 Fig).

Additional references:

Gaonkar CC, Kooistra WHCF, Lange CB, Montresor M, Sarno D. Two new species in the *Chaetoceros socialis* complex (Bacillariophyta): *C. sporotruncatus* and *C. dichatoensis*, and characterization of its relatives, *C. radicans* and *C. cinctus*. J Phycol. 2017;53(4):889-907. doi: 10.1111/jpy.12554.

Kooistra WHCF, Sarno D, Hernández-Becerril DU, Assmy P, Di Prisco C, Montresor M. Comparative molecular and morphological phylogenetic analyses of taxa in the Chaetocerotaceae (Bacillariophyta). Phycologia. 2010;5:471-500

***C. gelidus*** Chamnansinp, Li, Lundholm & Moestrup **(Type A*)**

Chamnansinp A, Li Y, Lundholm N, Moestrup Ø. Global diversity of two widespread, colony-forming diatoms of the marine plankton, *Chaetoceros socialis* (syn. *C. radians*) and *Chaetoceros gelidus* sp. nov. J Phycol. 2013;49:1128–41. doi: 10.1111/jpy.12121.

Type locality: Skovshoved Harbour, The Sound (Øresund), Denmark

The strains included in this study are those reported in Gaonkar et al. (2017); the sequences grouped in a single terminal clade in both 18S and 28S trees (S2 Fig; S4 Fig).

***C. dichatoensis* Gaonkar, Montresor et Sarno (Type A*)**

Gaonkar CC, Kooistra WHCF, Lange CB, Montresor M, Sarno D. Two new species in the *Chaetoceros socialis* complex (Bacillariophyta): *C. sporotruncatus* and *C. dichatoensis*, and characterization of its relatives, *C. radicans* and *C. cinctus*. J Phycol. 2017;53(4):889-907. doi: 10.1111/jpy.12554.

Type locality: Las Cruces, Chile

The strains included in this study are those reported in Gaonkar et al. (2017); the sequences grouped in a single terminal clade in both 18S and 28S trees (S2 Fig; S4 Fig).

***C. sporotruncatus*** **Gaonkar, Kooistra et Lange (Type A*)**

Gaonkar CC, Kooistra WHCF, Lange CB, Montresor M, Sarno D. Two new species in the *Chaetoceros socialis* complex (Bacillariophyta): *C. sporotruncatus* and *C. dichatoensis*, and characterization of its relatives, *C. radicans* and *C. cinctus*. J Phycol. 2017;53(4):889-907. doi: 10.1111/jpy.12554.

Type locality: Las Cruces, Chile

The strains included in this study are those reported in Gaonkar et al. (2017); the sequences grouped in a single terminal clade in the 28S tree; the 18S was available for a single strain (S2 Fig; S4 Fig).

**CLADE VII**

This large clade includes four distinct subclades.

**SUB-CLADE VIIa**

This subclade includes eight species all of which share the presence of one single chloroplast and the tendency to rapidly become single-celled in culture. At least three species (*Chaetoceros* sp*.* clade Na12A3, *C. circinalis* and *C. affinis*) share a morphology resembling that of *C. affinis* or its varieties, as reported in Cupp (1943). Further studies are in progress to better define those species.

Cupp EE. Marine plankton diatoms of the west coast of north America. Bull Scripps Inst Oceanogr. 1943;5:1-237.

***Chaetoceros* sp. clade Na13C2** **(Type E)**

Two strains were isolated from the Gulf of Naples, which had identical 28S sequences; 18S sequence was obtained only for one strain (S2 Fig; S4 Fig).

Straight chains with oval apertures generally constricted in the central portion (Fig. 24 in S1 File). Single chloroplast. Long intercalary setae originate from the valve corners and cross at the chain margin. Setae oriented in the apical plane. Terminal setae with a U-shaped appearance.

***Chaetoceros* sp. clade Na12A3 (Type E)**

Seven strains were isolated from the Gulf of Naples, which exhibited identical 28S sequences; 18S sequences were obtained only for two strains (S2 Fig; S4 Fig). The 28S sequences are identical to that of strain PMF-E1 identified as *C. affinis* by Bosak & Sarno (2017).

Straight chains with narrow apertures (Fig. 25 in S1 File). Single chloroplast. Setae arise from the valve corners and cross immediately at the chain margin. Terminal setae U-shaped and slightly thicker than intercalary ones. Spores reported for strain PMF-E1 (Bosak & Sarno, 2017); not observed in the Neapolitan strains.

Additional reference:

Bosak S, Sarno D. The planktonic diatom genus *Chaetoceros* Ehrenberg (Bacillariophyta) from the Adriatic Sea. Phytotaxa. 2017;314:001–44.

***Chaetoceros* sp. clade Na17B2 (Type E)**

Two Neapolitan strains shared identical 18S and 28S sequences (S2 Fig; S4 Fig). Solitary cells with one chloroplast (Fig. 26 in S1 File). Long setae arise from the margin of the valves, have a U-shape and tend to cross their tips. Resting spores not observed.

***C. circinalis* (Meunier) Jensen & Moestrup (Type B)**

Meunier A. Microplankton de la mer Flamande. Bruxelles: Hayez, Imprimeur de l'Académie royale de Belgique; 1913.

Type locality: North Sea

Only one strain was isolated from the Gulf of Naples for which both 18S and 28S sequences were obtained (S2 Fig; S4 Fig). The gross morphology and the setae orientation (Fig. 27 in S1 File) matched the descriptions provided by Jensen & Moestrup (1988) and Bosak & Sarno (2017).

Short chains with narrow apertures. Single cells often present in culture. Single chloroplast. Setae arise from valve corners and cross at chain margin. Intercalary setae markedly curved and bend in a curve around the chain. Resting spore not observed.

Additional references:

Bosak S, Sarno D. The planktonic diatom genus *Chaetoceros* Ehrenberg (Bacillariophyta) from the Adriatic Sea. Phytotaxa. 2017;314:001–44.

Jensen KG, Moestrup Ø. The genus *Chaetoceros* (Bacillariophyceae) in inner Danish coastal waters. Opera Botanica. 1998;133:1-68.

***Chaetoceros* sp. clade CDP22** **(Type E)**

Only one strain was isolated from the Gulf of Naples (S2 Fig; S4 Fig). Solitary cells with single chloroplast (Fig. 28 in S1 File). Setae arise at valve corners and are perpendicular to pervalvar axis. Resting spores not observed.

***Chaetoceros affinis* (Type A)**

Lauder HS. Remarks on the marine Diatomaceae found at Hong Kong, with descriptions of new species. Trans Micr Soc London, n s. 1864;12:75-9.

Type locality: Hong Kong

The sequences grouped in a single terminal clade in both 18S and 28S trees (S2 Fig; S4 Fig). The morphology of the strains and the 28S sequence match what has been identified as *C. affinis* in Kooistra et al. (2010) for the presence of thicker terminal setae. However, the circumscription of this species requires further investigations.

Here we provide LM pictures of a strain from the Gulf of Naples (Fig. 29 in S1 File).

Additional reference:

Kooistra WHCF, Sarno D, Hernández-Becerril DU, Assmy P, Di Prisco C, Montresor M. Comparative molecular and morphological phylogenetic analyses of taxa in the Chaetocerotaceae (Bacillariophyta). Phycologia. 2010;5:471-500

***C. diversus* Cleve (Type C)**

Cleve PT. Examination of diatoms found on the surface of the sea of Java. Bihang Till Konglica Svenska Vetenskaps- Akademiens Handlingar. 1873;11:3-13.

Type locality: Java Sea, Indonesia

Strains from the Gulf of Naples separated into two groups: *C. diversus*  and *C. diversus* 2, their 18S and 28S core sequences showed just a few base differences and grouped into sister clades only in the 18S tree (S2 Fig; S4 Fig). Yet, the two groups of sequences showed marked differences in the types and positions of introns, whereas the pattern of introns was virtually the same within the groups.

The two groups of strains differed also morphologically. Strains identified as *C. diversus* 1 (Fig. 30 in S1 File) form short chains with narrow apertures. Single chloroplast. Two types of intercalary setae are present: thinner, straight setae and thicker specialized ones, which however disappear with time in culture. Intercalary setae arise from valve corners, cross immediately and diverge on the apical plane. The specialized setae are longer and usually slightly bent and thicker at their distal end. Terminal setae are thin and exhibit a very broad and progressively widening V shape. Resting spores not observed. Strains identified as *C. diversus* 2 (Fig. 31 in S1 File) differ from *C. diversus* 1 in having narrow U-shaped terminal setae and intercalary specialized setae, which are more bent towards the terminal end of the chain.

Additional reference:

Moreno Ruiz JL, Soto PJ, Zamudio ME, Hernández-Becerril DU, Licea SD. Morphology and taxonomy of *Chaetoceros diversus* (Bacillariophyceae) based on material from the southern Gulf of Mexico. Diatom Res. 1993;8:419-28.

**SUB-CLADE VIIb**

***C. constrictus* Gran (Type B)**

Gran HH. Protophyta: Diatomaceae, Silicoflagellata og Cilioflagellata. Norske Nordhans-Expedition 1876-1878, XXIV Botanik Christiania1897.

Type locality: North Sea, Skagerak

The morphology of the strains isolated in this study (Fig. 32) matched the descriptions provided by Jensen & Moestrup (1998), Lee et al. (2014) and Shevchenko et al. (2006). The sequences grouped in a single terminal clade in both 18S and 28S trees (S2 Fig; S4 Fig).

Cells united to form chains with lanceolate aperture. Two chloroplasts. Mantle with a distinct constriction near the margin. Terminal setae V-shaped and, at times, slightly thicker than the intercalary ones. Intercalary setae arise on the valve corner and cross immediately at the chain axis. Valve with a central annulus, with radially branching costae and a rim on the marginal ridge. Terminal valve with a central slit-shaped rimoportula showing a small tube externally. Terminal valve face ornamented with small spines. Setae polygonal in cross section, with spirally arranged shark-fin spines, longitudinally arranged poroids, and scattered large pores. Girdle bands ornamented by transverse costae and minute poroids Resting spores comprised of unequally convex valves. Primary valve shows long stout spines, sometimes bifurcated at the tip. Secondary valve ornamented with shorter spines. Mantle of the primary valve possesses small silica ornamentations.

Additional references:

Jensen KG, Moestrup Ø. The genus *Chaetoceros* (Bacillariophyceae) in innner Danish coastal waters. Opera Botanica. 1998;133:1-68. Lee SD, Joo HM, Lee JH. Critical criteria for identification of the genus *Chaetoceros* (Bacillariophyta) based on setae ultrastructure. II. Subgenus *Hyalochaete*. Phycologia. 2014;53:614-38. doi: 10.2216/14-51r2.1.

Shevchenko OG, Orlova TY, Hernández-Becerril DU. The genus *Chaetoceros* (Bacillariophyta) from Peter the Great Bay, Sea of Japan. Bot Mar. 2006;49:236-58.

***C. seiracanthus* Gran (Type B)**

Gran HH. Protophyta: Diatomaceae, Silicoflagellata og Cilioflagellata. Norske Nordhans-Expedition 1876-1878, XXIV Botanik Christiania1897.

Type locality: Skagerrak

Two strains were isolated from the Gulf of Naples, which had identical 28S and 18S sequences (S2 Fig; S4 Fig). The gross morphology of the vegetative cells and colonies (Fig. 33 in S1 File) is similar to that of *C. diadema* from which it is differentiated by the morphology of the spore (Jensen & Moestrup, 1998).

Chains short with wide hexagonal apertures. Single chloroplast. Intercalary setae emerge on valve corners, have a short basal part, cross slightly outside the chain margin and are oriented in all directions. Terminal setae U-shaped. A few resting spores were observed with an almost flattened primary valve bearing short spines. The secondary valve is ornamented with spines, markedly vaulted in the center and constricted at the base.

Additional reference:

Jensen KG, Moestrup Ø. The genus *Chaetoceros* (Bacillariophyceae) in innner Danish coastal waters. Opera Botanica. 1998;133:1-68.

***Chaetoceros* sp. clade Na13C1 (Type E)**

The 28S sequence of the strain isolated from the Gulf of Naples matches with the one identified as *C. diadema* (strain DH21) in Kooistra et al. (2010). We provide also the 18S sequence (S2 Fig; S4 Fig). The gross morphology of the vegetative cells and colonies (Fig. 34 in S1 File) is similar to *C. seiracanthus* and *C. diadema*.

Chains with large hexagonal aperture. Single chloroplast. Intercalary setae emerge on valve corners, have a short basal part, cross slightly outside the chain margin and are oriented in all directions. Terminal setae U-shaped. Valve face with dichotomously branched costae radiating from the central annulus. Terminal valve with a central slit-shaped rimoportula. Girdle band with transverse costae perforated with minute pores. Resting spores not observed.

Additional reference:

Kooistra WHCF, Sarno D, Hernández-Becerril DU, Assmy P, Di Prisco C, Montresor M. Comparative molecular and morphological phylogenetic analyses of taxa in the Chaetocerotaceae (Bacillariophyta). Phycologia. 2010;5:471-500

***C*. *rotosporus* Li, Lundholm & Moestrup (Type A)**

Li Y, Lundholm N, Moestrup Ø. *Chaetoceros rotosporus* sp. nov. (Bacillariophyceae), a species with unusual resting spore formation. Phycologia. 2013;52(6):600-8. doi: doi:10.2216/13-168.1.

Type locality: Daya Bay, South China Sea.

The sequences grouped in a single terminal clade in both 18S and 28S trees (S2 Fig; S4 Fig).

***C. diadema* (Ehrenberg) Gran (Type C)**

Gran HH. Protophyta: Diatomaceae, Silicoflagellata og Cilioflagellata. Norske Nordhans-Expedition 1876-1878, XXIV Botanik Christiania1897.

Type locality: Peru

In both trees, two clades were recorded, grouping sequences of strains identified as *C. diadema* (S2 Fig; S4 Fig). One clade (*C. diadema* 1) included strains isolated from the Gulf of Naples and from Chile; the 28S sequences matched with those (SZN-B412, SZN-B414, SZN-B444) illustrated in Kooistra et al. (2010). In this latter paper, strain DH21 was also identified as *C. diadema*, but the analysis of other sequences proved that this is a distinct genotype (*C*. sp. clade Na13C1). The second clade (*C. diadema* 2) included two strains isolated from Chile. Here we provide 18S of the two clades and illustrations of *C. diadema* 2 (Fig. 35 in S1 File), which shows the same morphology as *C. diadema* 1 described in Kooistra et al. (2010).

Additional reference:

Kooistra WHCF, Sarno D, Hernández-Becerril DU, Assmy P, Di Prisco C, Montresor M. Comparative molecular and morphological phylogenetic analyses of taxa in the Chaetocerotaceae (Bacillariophyta). Phycologia. 2010;5:471-500

**SUB-CLADE VIIc**

***Chaetoceros* sp. clade Va7D2 (Type E)**

The 28S sequences of the strains isolated from the Gulf of Naples (Fig. 36 in S1 File) formed a clade. We also provide the V4 region of the 18S sequence for one of the strains (S2 Fig; S4 Fig).

Chains with hexagonal apertures of variable size. Intercalary setae emerge on valve margin and cross just outside the chain axis. Terminal setae V-shaped. Setae pentagonal in cross-section, ornamented with lines of slit-shaped poroids and shark-fin spines. Valves with radially branched costae departing from a central annulus. Terminal valve with a central, slit-shaped rimoportula exhibiting an elongated tube externally. Girdle bands with transverse costae. Resting spores not observed.

***Chaetoceros* sp. clade Na28A1 (Type E)**

The sequences of the two strains isolated from the Gulf of Naples grouped in a terminal clade in both trees (S2 Fig; S4 Fig).

Chains with elliptic-lanceolate apertures (Fig. 37 in S1 File). Single chloroplast. Intercalary setae arise from valve apices, fuse at the chain margin and are oriented in all directions. Terminal setae oriented in a narrow U shape, sometimes crossing each other in the distal part. Setae ornamented with spirally arranged shark-fin spines, parallel rows of poroids and large pores at irregular intervals. Intercalary setae show a petal-like collar at their base along with a hyaline rim. Intercalary valves with dichotomous branching costae radiating from a central annulus. Girdle band with transverse costae and perforated with minute pores. Resting spores not observed.

***C.* cf*. vixvisibilis* Schiller in Hustedt (Type D)**

For *C. vixvisibilis:* Hustedt F. Die Kieselalgen Deutschlands, Österrreichs und der Schweiz under Berücksichtigung der übrigen Länder Europas sowie der angrenzenden Meeresgebiete. 1. Teil. Leipzig: Akademische Verlaggesellschaft; 1930. 920 p.

Type locality: Adriatic Sea

The sequences grouped in a single terminal clade in both 18S and 28S trees (S2 Fig; S4 Fig). We identified these strains from the Gulf of Naples as *C*. cf. *vixvisibilis* because we did not observe spores in our cultures. Spore morphology is the most distinctive character for the identification of *C*. *vixvisibilis*. Moreover, the setae of *C*. cf. *vixvisibilis* are ornamented with spines (Fig. 38 in S1 File), which were not reported in natural material of *C. vixvisibilis* from the Adriatic Sea (Hernández-Becerril et al. 2010).

Long chains with narrow lanceolate apertures. Two chloroplasts. Long intercalary setae emerge from the corner of the valves, cross over immediately and are directed perpendicular to chain axis. Valves with a central annulus and irregularly branched costae. Terminal valve with a central rimoportula that has a small tube externally. Setae circular in cross section, ornamented with spirally organized spines and rows of poroids, and large pores regularly distributed. Girdle bands with transverse costae and minute poroids. Resting spores not observed.

Additional references:

Hernàndez-Becerril DU, D. V, Bosak S, Djakovac T. Morphology and ecology of the diatom *Chaetoceros vixvisibilis* (Chaetocerotales, Bacillariophyceae) from the Adriatic Sea. J Plankton Res. 2010.

Bosak S, Sarno D. The planktonic diatom genus *Chaetoceros* Ehrenberg (Bacillariophyta) from the Adriatic Sea. Phytotaxa. 2017;314:001–44.

***C. anastomosans* Grunow in Van Heurck (Type B)**

van Heurck HF. Synopsis des Diatomées de Belgique. Anvers: Edité par l'Auteur; 1885. Texte, 235 p. Atlas, 120 p. p.

Type locality: Adriatic Sea

The morphology of the strains isolated in this study (Fig. 39 in S1 File) matched the descriptions provided by Hernández-Becerril & Flores Granados (1998) and Lee et al. (2014). The sequences grouped in a single terminal clade in both 18S and 28S trees (S2 Fig; S4 Fig).

Straight or slightly curved chains with wide octagonal apertures. Two chloroplasts. Valves ornamented with costae radiating from the central annulus. Terminal valve with a central slit-shaped rimoportula with a small tube externally. Setae with a long basal part emerge slightly inside the valve margin. Setae of adjacent cells joined together by a siliceous bridge. Setae circular in cross section, with spirally arranged shark-fin spines and rows of minute poroids. Girdle bands ornamented with transverse costae. Resting spores biconvex, with stout spines on both valves.

Additional references:

Hernández-Becerril DU, Flores Granados C. Species of the diatom genus *Chaetoceros* (Bacilariophyceae) in the plankton from the southern Gulf of Mexico. Bot Mar. 1998;41:505-19.

Lee SD, Joo HM, Lee JH. Critical criteria for identification of the genus *Chaetoceros* (Bacillariophyta) based on setae ultrastructure. II. Subgenus *nf*. Phycologia. 2014;53:614-38. doi: 10.2216/14-51r2.1.

***C. dayaensis* Li & Zhu (Type A*)**

Li Y, Zhu S, Lundholm N, Lü S. Morphology and molecular phylogeny of *Chaetoceros dayaensis* sp. nov. (Bacillariophyceae), characterized by two 90° rotations of the resting spore during maturation. J Phycol. 2015;51(3):469-79. doi: 10.1111/jpy.12290.

Type locality: Daya Bay, South China Sea

Only the 28S sequences reported in Li et al. (2015) were available (S4 Fig).

***Chaetoceros* sp. clade Na26B1 (Type E)**

The sequences of the strains isolated from the Gulf of Naples (Fig. 40 in ormationnformation) were recovered in a clade in the 28S tree; we also provide the 18S sequence for one of the strains (S2 Fig; S4 Fig).

Chains with narrow apertures. Single chloroplast. Intercalary setae arise on valve corners and cross on chain margin, directed in the apical plane. Terminal setae straight, almost parallel to the chain axis, forming a broad V-shape. Setae circular in cross-section, ornamented with numerous spirally arranged spines and longitudinal poroids. Several poroids are scattered on the valve face, with the exception of the central area; dichotomously branched costae radiating from the central annulus. Terminal valve showing a central small slit-shaped rimoportula with a short tube externally. Resting spores not observed.

***Chaetoceros* sp. clade Na11C3 (Type E)**

The sequences of the strains isolated from the Gulf of Naples (Fig. 41 in S1 File) formed a clade in the 18S tree but a grade in the 28S tree with the aforementioned species recovered inside it (S2 Fig; S4 Fig).

Chains with apertures of variable size. Single chloroplast. Intercalary setae arise on valve corners and cross on chain margin, directed in all directions. Terminal setae are straight, almost parallel to chain axis in the apical plane. Setae circular in cross-section and possess numerous spirally arranged spines and minute poroids. Valves face with dichotomously branched costae radiating from the central annulus. Terminal valve with a central slit-shaped rimoportula showing an external, short, flattened tube. Girdle bands with transverse costae perforated with minute poroids. Resting spores not observed.

**SUB-CLADE VIId**

***C*. *throndsenii* Marino, Montresor & Zingone (Type B)**

Marino D, Montresor M, Zingone A. *Miraltia throndsenii* gen. nov., sp. nov., a planctonic diatom from the Gulf of Naples. Diatom Res. 1987;2:205-11.

Type locality: Gulf of Naples, Italy, Tyrrhenian Sea, Mediterranean Sea.

The cell morphology of the strains isolated in this study (Fig. 42 in S1 File) matched the original description. The sequences grouped in a single terminal clade in both 18S and 28S trees (S2 Fig; S4 Fig).

Additional reference:

Marino D, Giuffé G, Montresor M, Zingone A. An electron microscope investigation on *Chaetoceros minimus* (Levander) comb. nov. and new observations on *Chaetoceros throndsenii* (Marino, Montresor and Zingone) comb. nov. Diatom Res. 1991;6:317-26.

***C*. *minimus* (Levander) Marino, Giuffrè, Montresor & Zingone (Type B)**

Levander KM. Zur Kenntnis der Rhizosolenien Finlands. Memoranda Societatis pro Fauna et Flora Fennica. 1904;30:112-7.

Type locality: Wiborg Bay, Finland.

Only one strain was isolated from the Gulf of Naples for which both 18S and 28S sequences were obtained (S2 Fig; S4 Fig). The single isovalvate cells possess two long setae that emerge each from one valve and run almost parallel to the pervalvar axis (Marino et al, 1991).

Additional reference:

Marino D, Giuffé G, Montresor M, Zingone A. An electron microscope investigation on *Chaetoceros minimus* (Levander) comb. nov. and new observations on *Chaetoceros throndsenii* (Marino, Montresor and Zingone) comb. nov. Diatom Res. 1991;6:317-26.

***C*. *laevisporus*** **Li, Boonprakob, Moestrup & Lundholm (Type A*)**

Li Y, Boonprakob A, Gaonkar CC, Kooistra W, Lange CB, Hernandez-Becerrill D, et al. Diversity in the globally distributed diatom genus *Chaetoceros* (Bacillariophyceae): Three new species from warm-temperate waters. PLoS ONE. 2017;12(1). doi: 10.1371/journal.pone.0168887.

Type locality: Mannai Island, Rayong Province, Thailand.

The sequences included in this study are those reported in Li et al. (2017); the sequences grouped in a single terminal clade in both 18S and 28S trees (S2 Fig; S4 Fig).

***C*. *mitra* (Bailey) Cleve (Type A*)**

Li Y, Boonprakob A, Gaonkar CC, Kooistra W, Lange CB, Hernandez-Becerrill D, et al. Diversity in the globally distributed diatom genus *Chaetoceros* (Bacillariophyceae): Three new species from warm-temperate waters. PLoS ONE. 2017;12(1). doi: 10.1371/journal.pone.0168887.

Type locality: Sea of Kamtschatka

The 28S and 18S sequences were available for a single strain (see Li et al. 2017) (S2 Fig; S4 Fig).

***C*. *mannaii*** Boonprakob, Li, Moestrup & Lundholm (Type A*)

Li Y, Boonprakob A, Gaonkar CC, Kooistra W, Lange CB, Hernandez-Becerrill D, et al. Diversity in the globally distributed diatom genus *Chaetoceros* (Bacillariophyceae): Three new species from warm-temperate waters. PLoS ONE. 2017;12(1). doi: 10.1371/journal.pone.0168887.

Type locality: Mannai Island, Rayong Province, Thailand.

The sequences included in this study are those reported in Li et al. (2017); the sequences grouped in a single terminal clade in both 18S and 28S trees (S2 Fig; S4 Fig).

***C*. *lorenzianus* Grunow (Type C)**

Grunow A. Über einige neue und ungenugend bekante Arten und Gattungen von Diatomaceen. Verhandlungen der Kaiserlich-Königlichen Zoologisch-Botanischen Gesellschaft in Wien. 1863;13:137-62, pls 13-14.

Type locality: Adriatic Sea

In both trees (S2 Fig; S4 Fig) two clades were detected: *C*. *lorenzianus* 1 (Fig. 43 in S1 File) and *C*. *lorenzianus* 2 (Fig. 44 in S1 File). Strains belonging to the two groups share similar morphology in LM. Chains are straight with lanceolate apertures. The straight intercalary setae originate from valve corners, cross at the chain margin and diverge immediately in the plane of the apical axis. Cells contain numerous plastids. The terminal setae are U-shaped. Spores were not observed.

Note that strains DH2, DH19 and DH26, identified as *C. lorenzianus* by Kooistra *et al.* (2010), were transferred to *C. decipiens* by Li et al. (2017).

Additional reference:

Li Y, Boonprakob A, Gaonkar CC, Kooistra W, Lange CB, Hernandez-Becerrill D, et al. Diversity in the globally distributed diatom genus *Chaetoceros* (Bacillariophyceae): Three new species from warm-temperate waters. PLoS ONE. 2017;12(1). doi: 10.1371/journal.pone.0168887.

***C. elegans*** Li, Boonprakob, Moestrup & Lundholm (Type A*)

Li Y, Boonprakob A, Gaonkar CC, Kooistra W, Lange CB, Hernandez-Becerrill D, et al. Diversity in the globally distributed diatom genus *Chaetoceros* (Bacillariophyceae): Three new species from warm-temperate waters. PLoS ONE. 2017;12(1). doi: 10.1371/journal.pone.0168887.

Type locality: Dapeng Bay, Guangdong Province, P. R. China.

The sequences included in this study are those reported in Li et al. (2017); the sequences grouped in a single terminal clade in both 18S and 28S trees (S2 Fig; S4 Fig).

***C*. *decipiens* Cleve (Type A)**

Cleve PT. On diatoms from the Arctic Sea. Bihang Till Konglica Svenska Vetenskaps- Akademiens Handlingar. 1873;1(13):1-28.

Type locality: North Atlantic

The sequences grouped in a single terminal clade in both 18S and 28S trees (S2 Fig; S4 Fig).

Additional reference:

Li Y, Boonprakob A, Gaonkar CC, Kooistra W, Lange CB, Hernandez-Becerrill D, et al. Diversity in the globally distributed diatom genus *Chaetoceros* (Bacillariophyceae): Three new species from warm-temperate waters. PLoS ONE. 2017;12(1). doi: 10.1371/journal.pone.0168887.
