# Supplementary material for: Annotated 18S and 28S rDNA reference sequences of taxa in the planktonic diatom family Chaetocerotaceae
Source: PLoS One. 2018 Dec 26;13(12):e0208929. doi: 10.1371/journal.pone.0208929 (PMC6306197; doi:10.1371/journal.pone.0208929)
Supplement: S1 File — Photographic illustrations (LM, SEM, TEM) of cells and frustule elements of selected strains of Bacteriastrum and Chaetoceros taxa included in this study. (PDF) [file pone.0208929.s012.pdf]

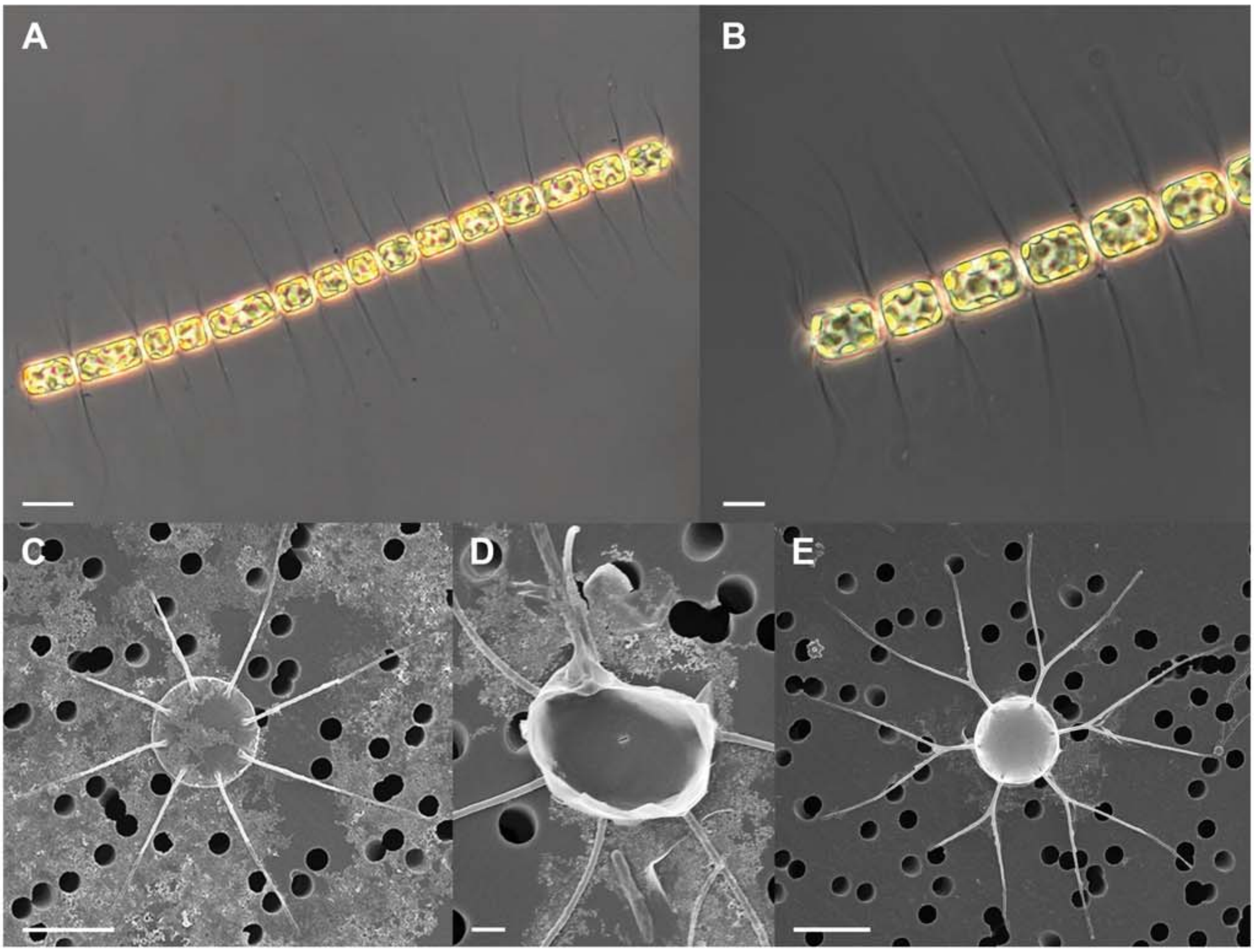

Fig. 1. *Bacteriastrum furcatum* 2, strain Na8A3. (A) Long heteropolar chain; (B) detail of the posterior end; (C) posterior terminal valve with radiating straight setae and central rimoportula; (D) anterior terminal valve with spiralling curved setae and central rimoportula; (E) intercalary valve with setae bifurcating in the valvar plane. A, B: LM; C-E: SEM. Scale bars = 20 µm (A); 10 µm (B, C, E); 2 µm (d).

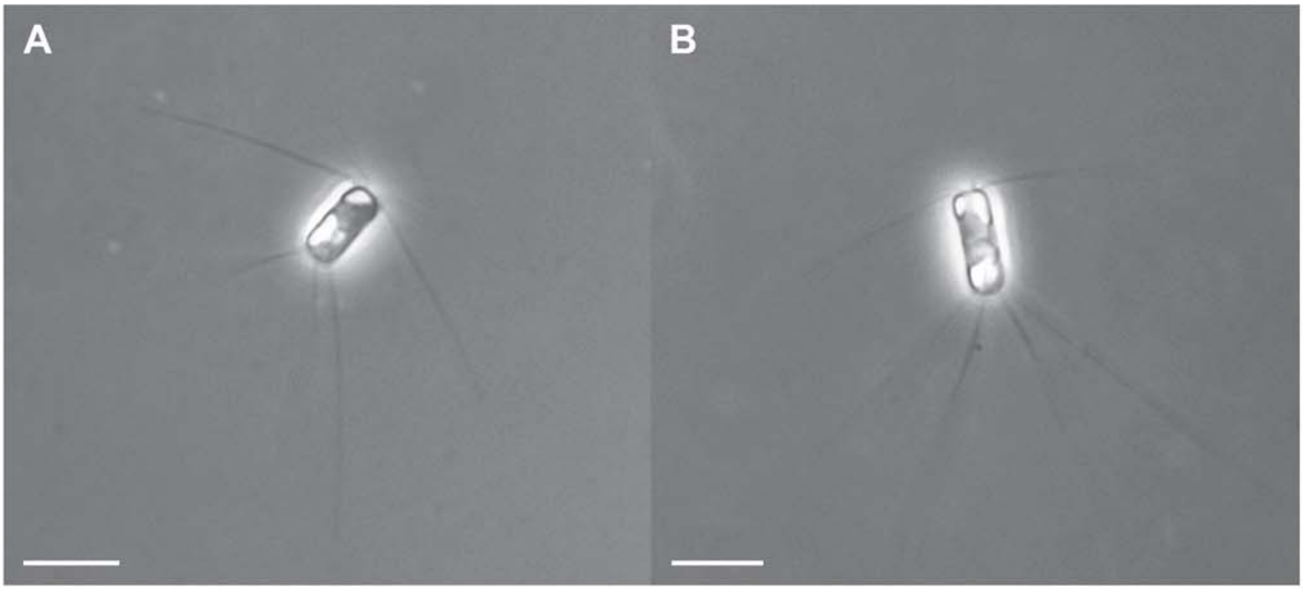

Fig. 2. *Bacteriastrum parallelum*, strain La1. (A, B) LM of single cells with heteropolar valves and the distinctive orientation of setae. Scale bars = 10 µm.

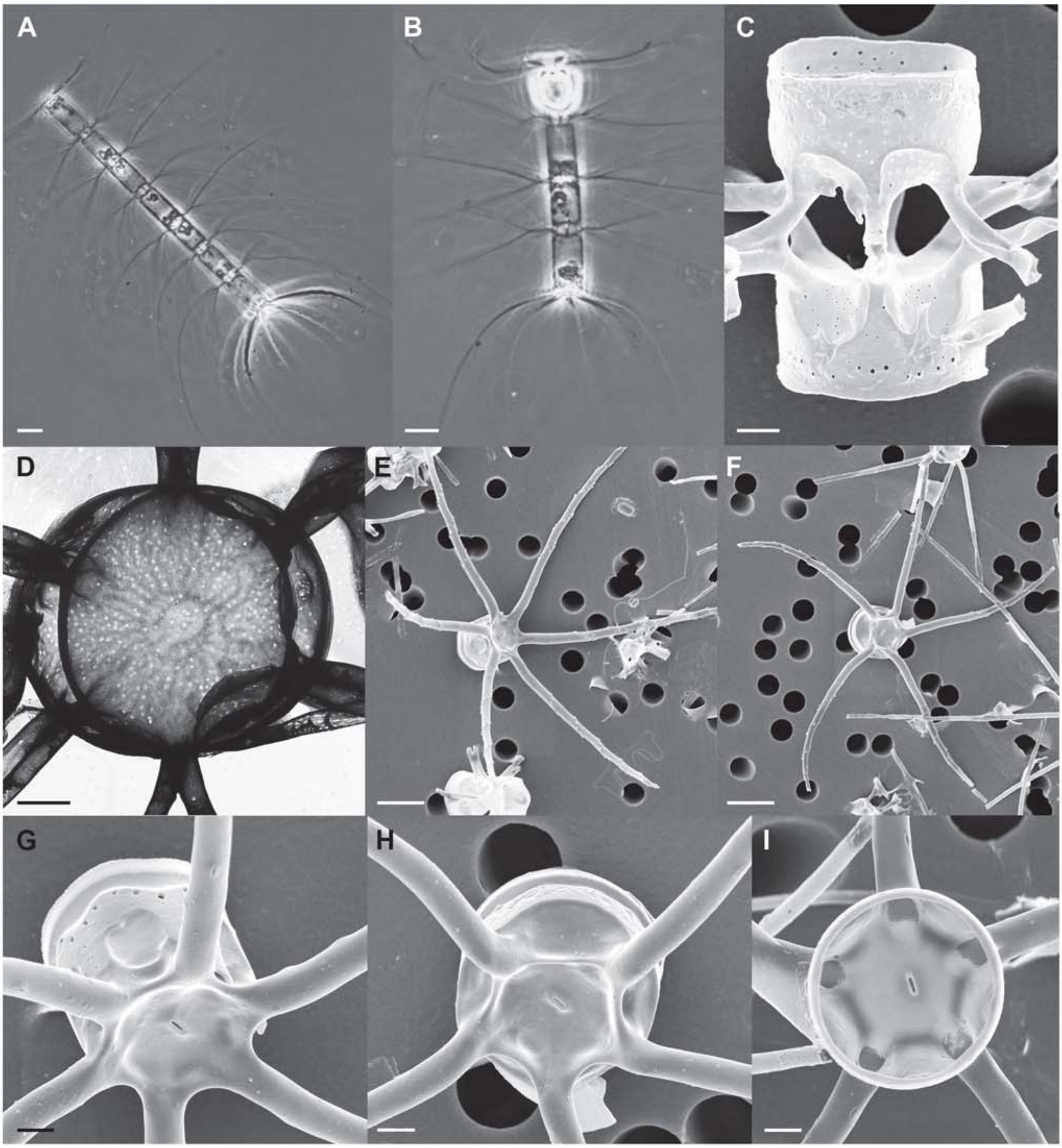

Fig. 3. *Bacteriastrum elegans,* strains Na25A3 (A-B) and Na29B1 (C-I). (A, B) Heteropolar chains; (C) junction between two cells in the chain; (D) intercalary valves; (E) posterior terminal valve with the characteristic umbrella-shaped setae; (F) anterior terminal valve with the characteristic pinwheel shaped setae; (G) detail of (E) showing the rimoportula on the posterior terminal valve; (H) detail of (F) showing the rimoportula on the anterior terminal valve; (I) internal view of a terminal valve showing the rimoportula. A, B: LM; C, E-I: SEM; D: TEM. Scale bars = 10 μm (A, B); 1 μm (C, D, G-I); 5 μm (E, F).

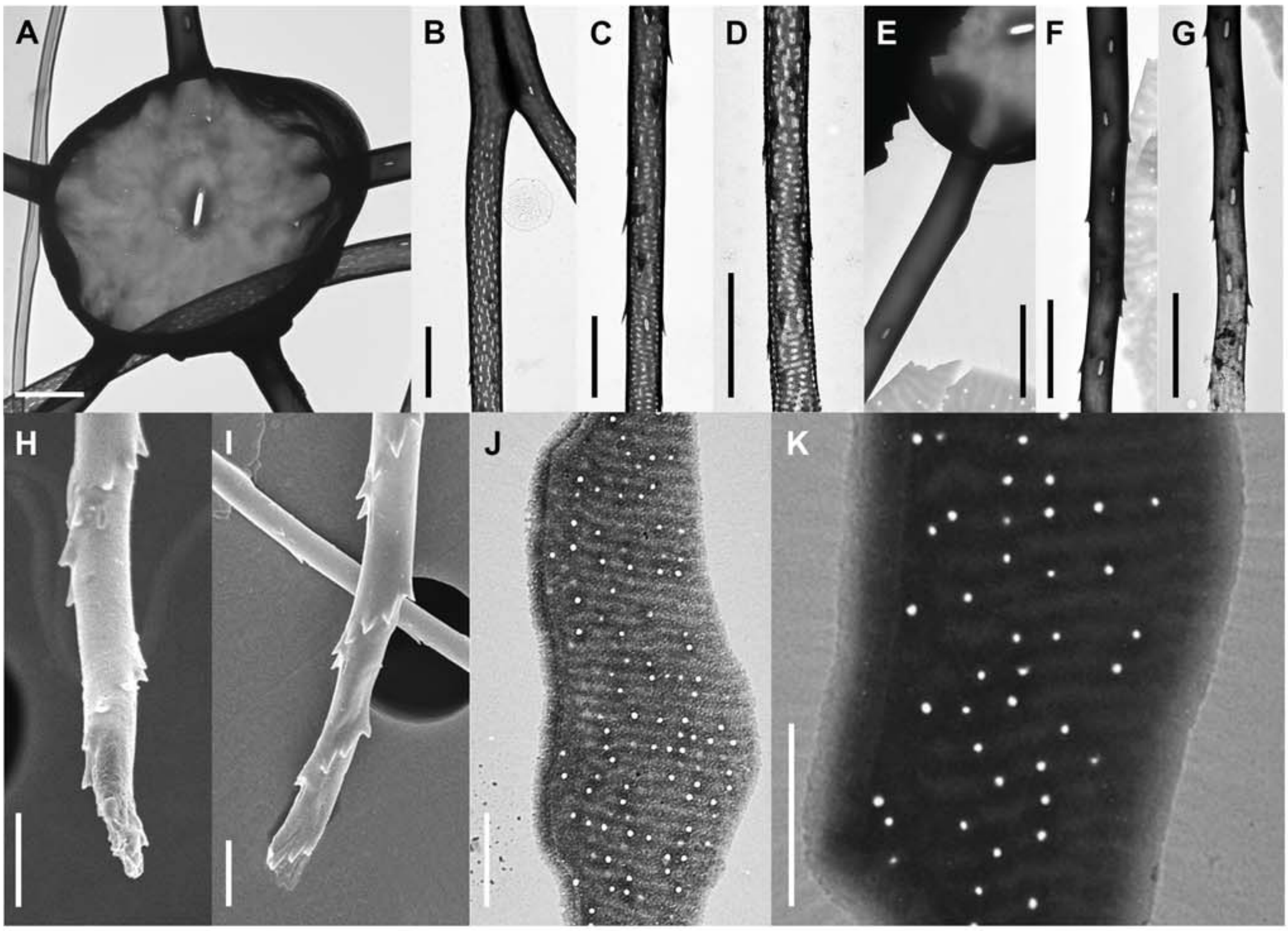

Fig. 4. *Bacteriastrum elegans*, strain Na29B1. (A) Terminal valve with slit-shaped rimoportula; detail of the basal (B), intermediate (C), and almost distal (D) portion of the intercalary setae; detail of the basal (E), intermediate (F) and almost distal (G) portion of the terminal setae; (H-I) distal portion of terminal setae; (J, K) girdle bands. A-G, J-K: TEM; H-I: SEM. Scale bars = 1 µm (A, B-E, H-K); 2 µm (F, G).

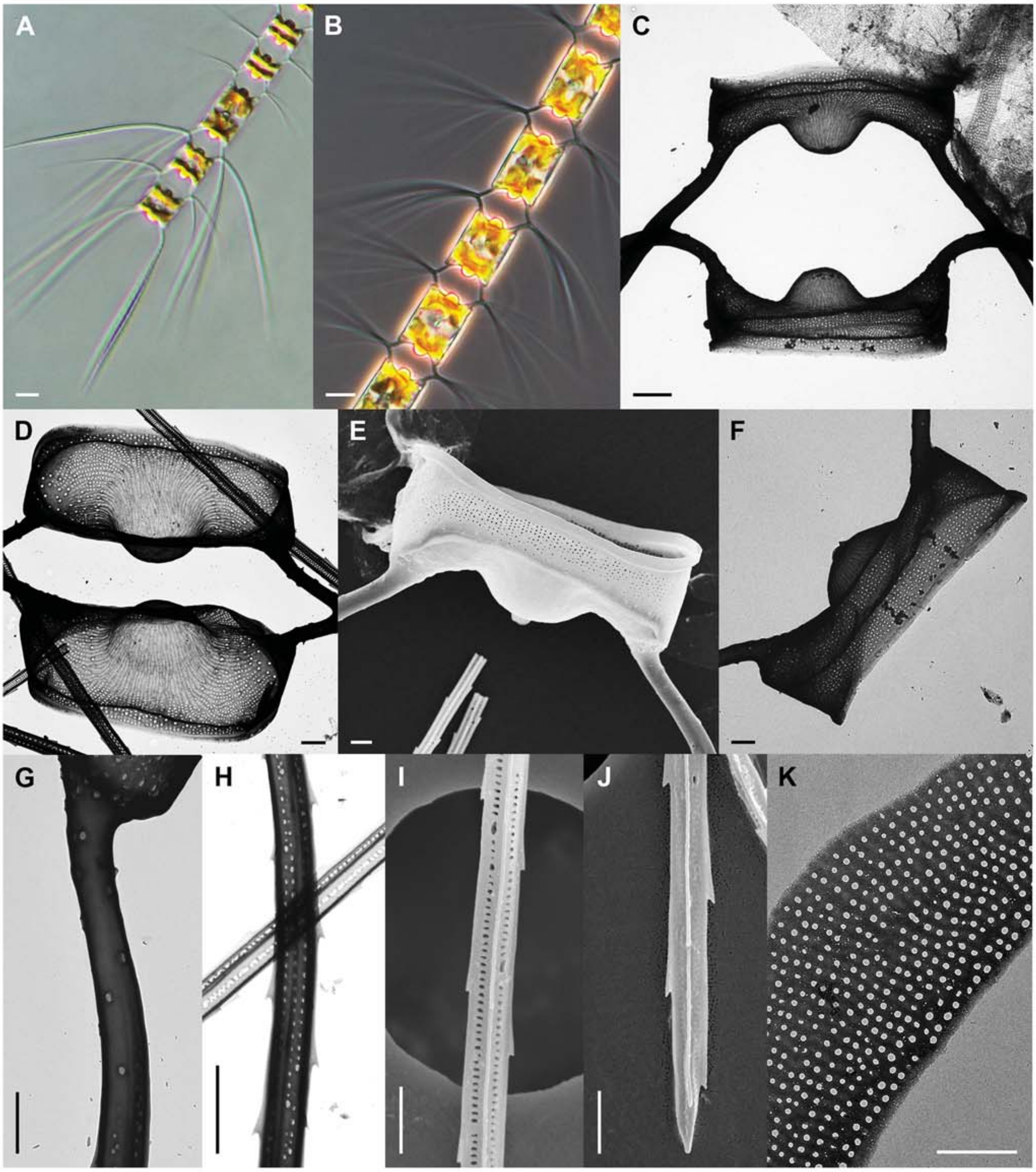

Fig. 5. *Chaetoceros protuberans*, strain Ch8C2. (A) Chain in broad girdle view with thicker terminal and specialized intercalary setae; (B) chain in broad girdle view with normal intercalary setae; (C) aperture; (D) intercalary valves; (E, F) terminal valves with a central tube-shaped rimoportula; (G) the basal portion of a terminal seta; (H, I) the middle portion of intercalary setae; (J) the distal part of a terminal seta; (K) girdle band. A, B: LM; C, D, F-H, K: TEM; E, I-J: SEM. Scale bars = 10 µm (A,B); 2 µm (C); 1 µm (D-K).

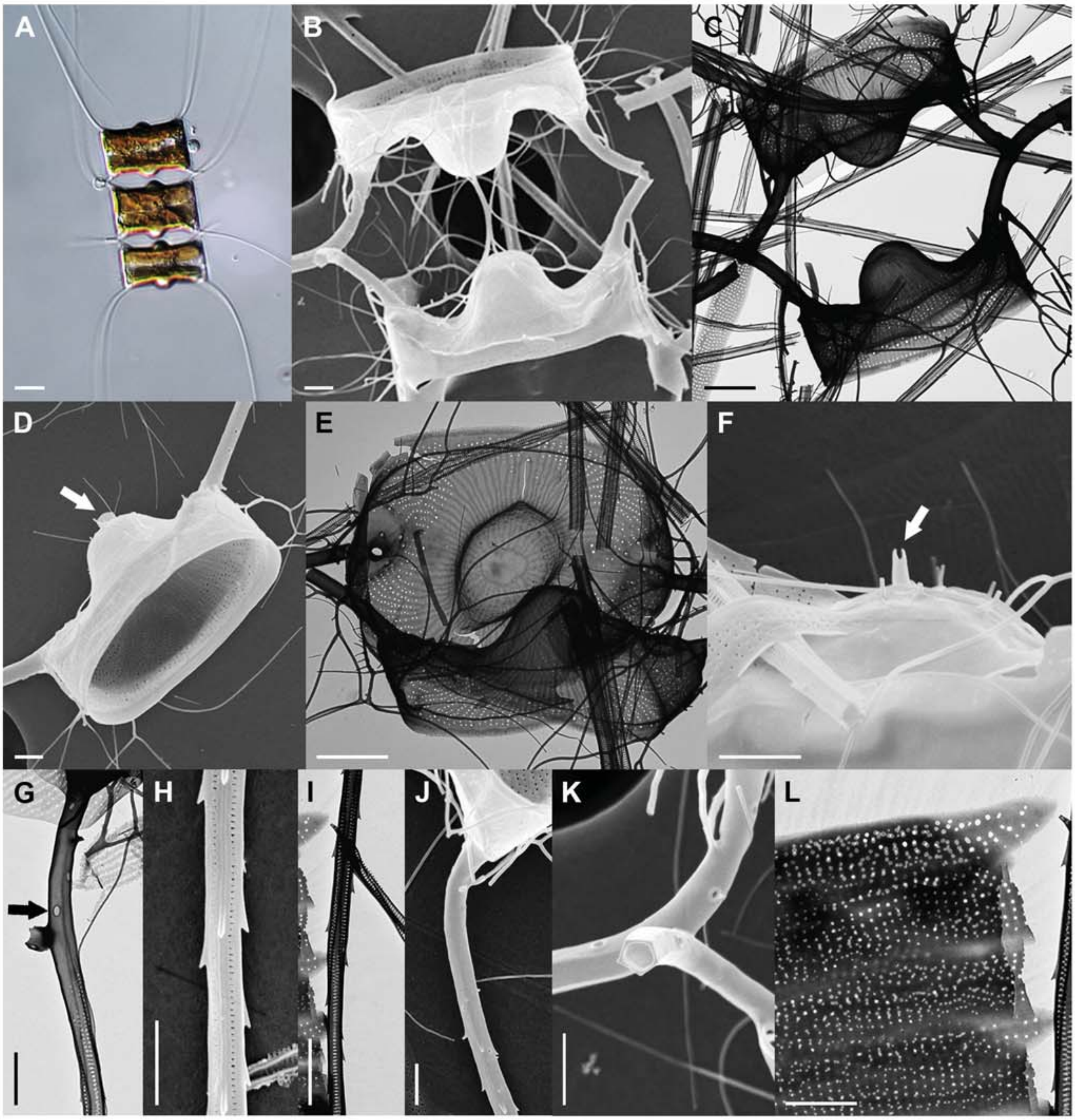

Fig. 6. *Chaetoceros didymus* 1, strains Ch6A3 (a) and Ch2B4 (B-L). (A) Chain in broad girdle view; (B-C) aperture between two adjacent c ells; note the capilli on the valves; (D) terminal valve with a central tube-shaped rimoportula (arrowed); (E) intercalary valve; (F) detail of terminal valve with rimoportula (arrowed); (G) detail of the basal part of an intercalary seta, a large pore is arrowed; (H, I) detail of the middle part of an intercalary seta; (J) detail of the basal part of a terminal seta; (K) cross section of an intercalary seta at the base; (L) girdle bands. A: LM; B, D, F, H, J, K: SEM; C, E, G, I, L: TEM; scale bars = 10 μm (A), 1 μm (B, D, F-H, J, K) and 2 μm (C, I, L).

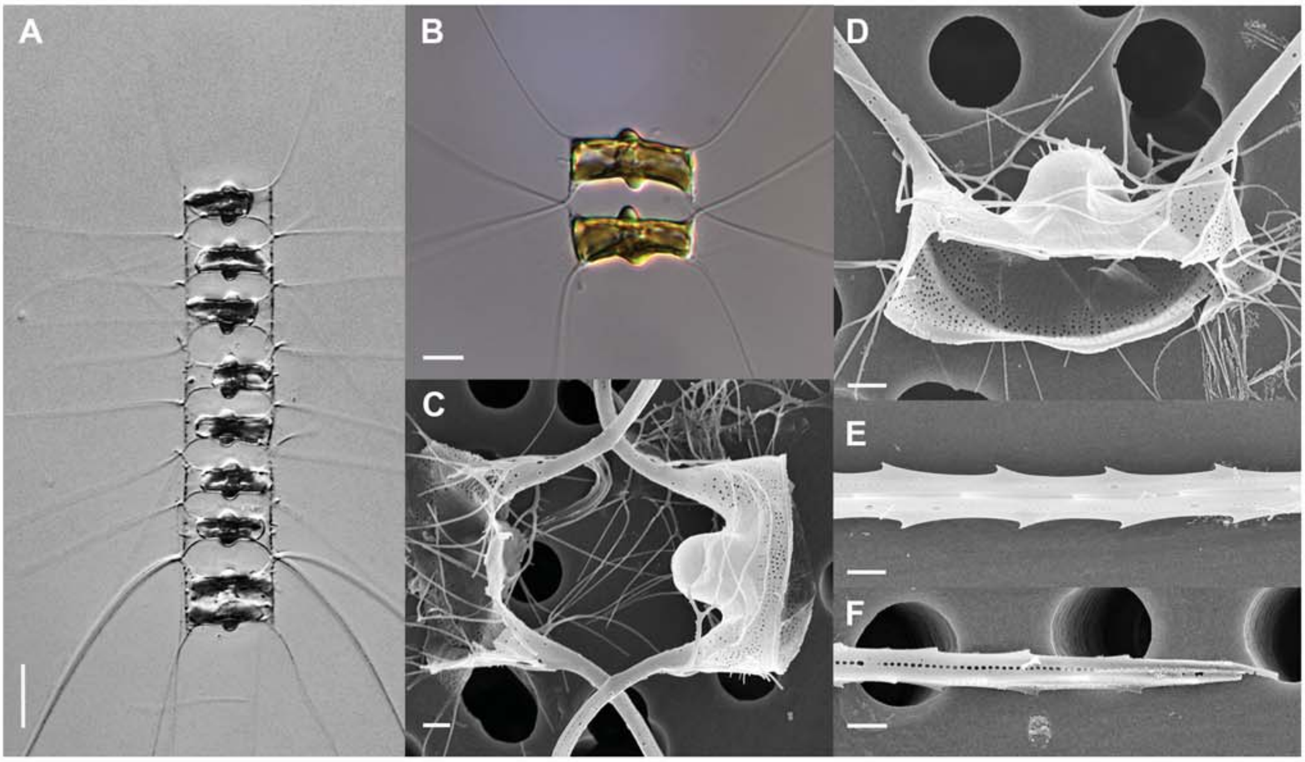

Fig. 7. *Chaetoceros didymus* 2, strain Na20B4. (A) Chain in broad girdle view, note the thicker specialized intercalary setae; (B) short colony in broad girdle view; (C) sibling valves with central protuberances and capilli; (D) terminal valve with capilli; (E) detail of the middle part of an intercalary seta; (F) detail of the distal part of an intercalary seta. A, B: LM; C-F SEM. Scale bars = 10 µm (A, B), 1 µm (C-F).

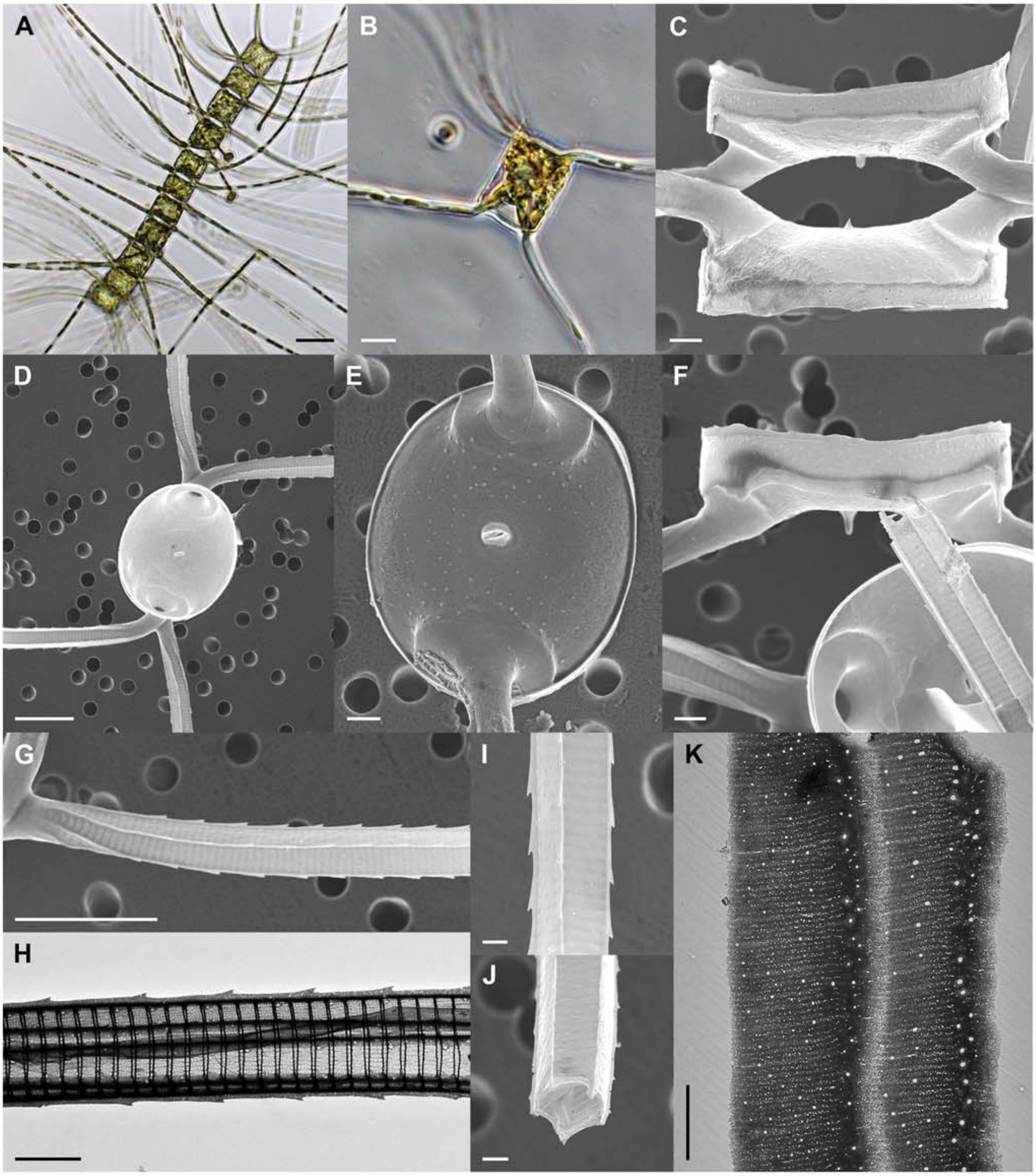

Fig. 8. *Chaetoceros eibenii*, strain Ro1B2. (A) Chain in broad girdle view; (B) single cell; (C) aperture between sibling valves with short tubular rimoportulae; (D) internal view of intercalary valve with a central slit-shaped rimoportula; (E) external view of a terminal valve with a central tube-shaped rimoportula, note poroids on the valve face; (F) broad girdle view of a terminal valve; (G) detail of a basal part of an intercalary seta; (H-I) Detail of the middle portion of an intercalary seta; (J) cross section of an intercalary seta; (K) girdle bands. A, B: LM; C-G, I, J: SEM; H, K: TEM. Scale bars = 20 µm (A), 10 µm (B, G), 2 µm (C, E, F, H, K), 1 µm (D, I, J).

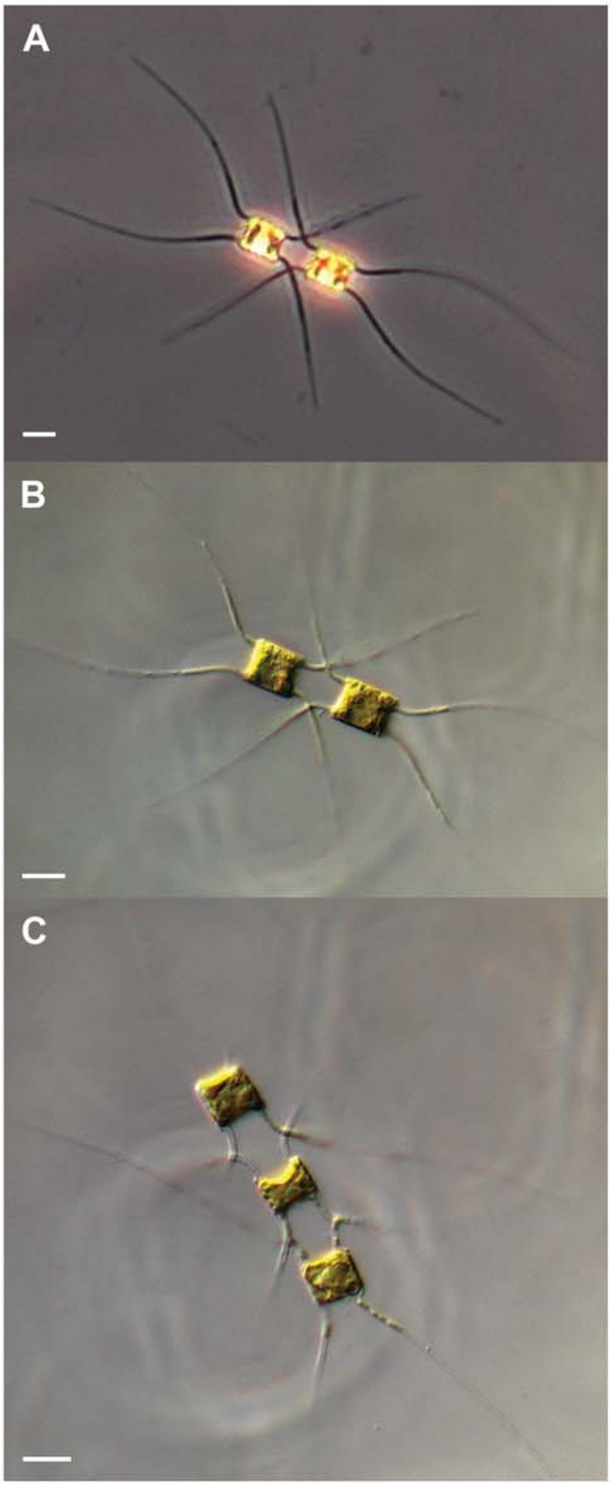

Fig. 9. *Chaetoceros* cf. *pseudodichaeta,* strain El1C1. (A-C) LM of short chains. Scale bars = 10 μm (A-C).

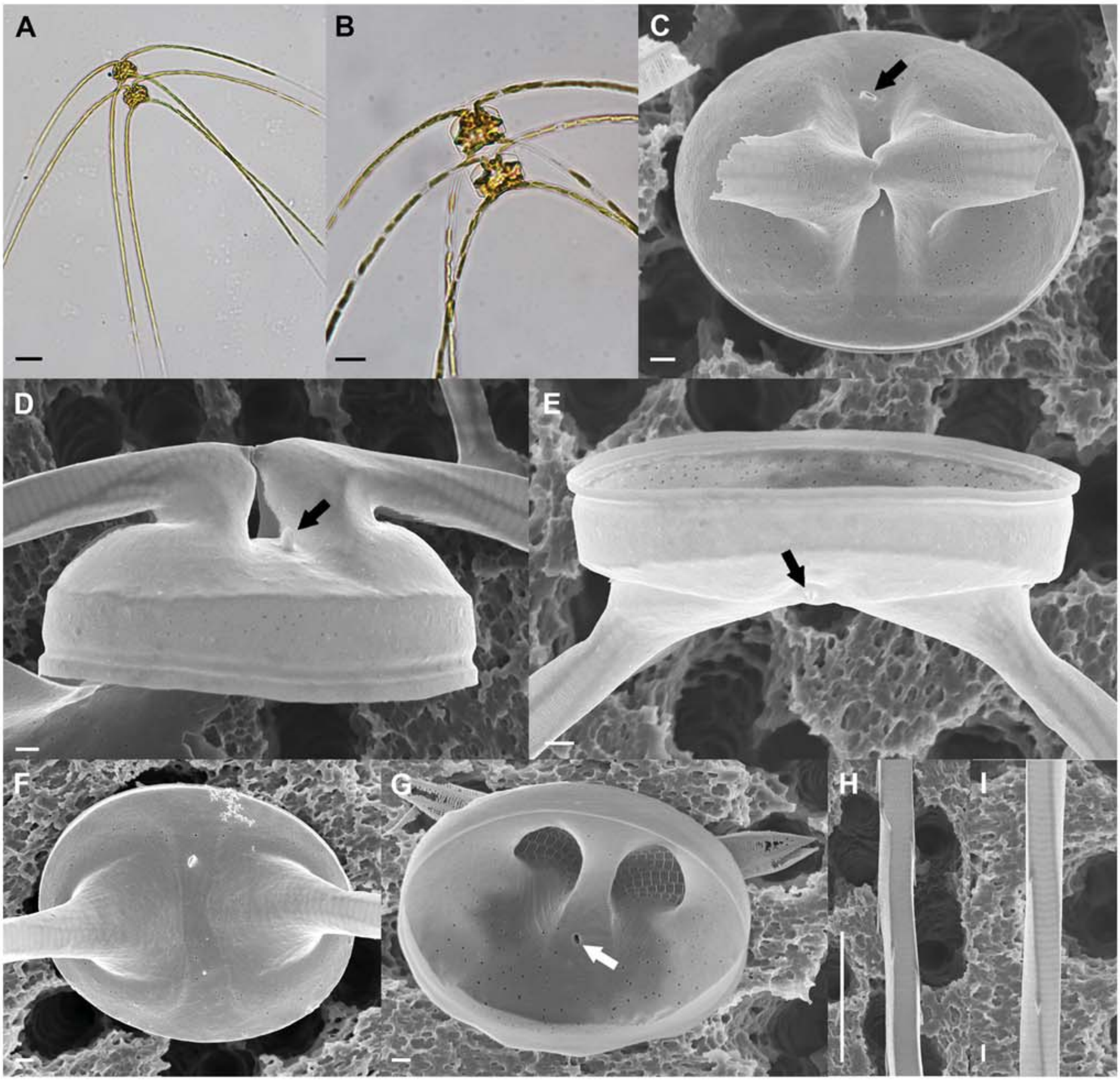

Fig. 10. *Chaetoceros peruvianus* 2, strain Ch11B4. (A, B) dividing cells; (C, D) epivalve with the junction between two setae in their proximal portion, the eccentric, tube-shaped rimoportula is arrowed; (E) hypovalve with rimoportula (arrowed): (F) epivalve in which the setae are not joined in their proximal portion; (G) internal view of the epivalve with the eccentric slit-shaped rimoportula (arrowed); (H, I) detail of a seta with shark-fin spines. A, B: LM; C-I: SEM. Scale bars = 20 µm (A), 10 µm (B, H); 1 µm (C-G, I).

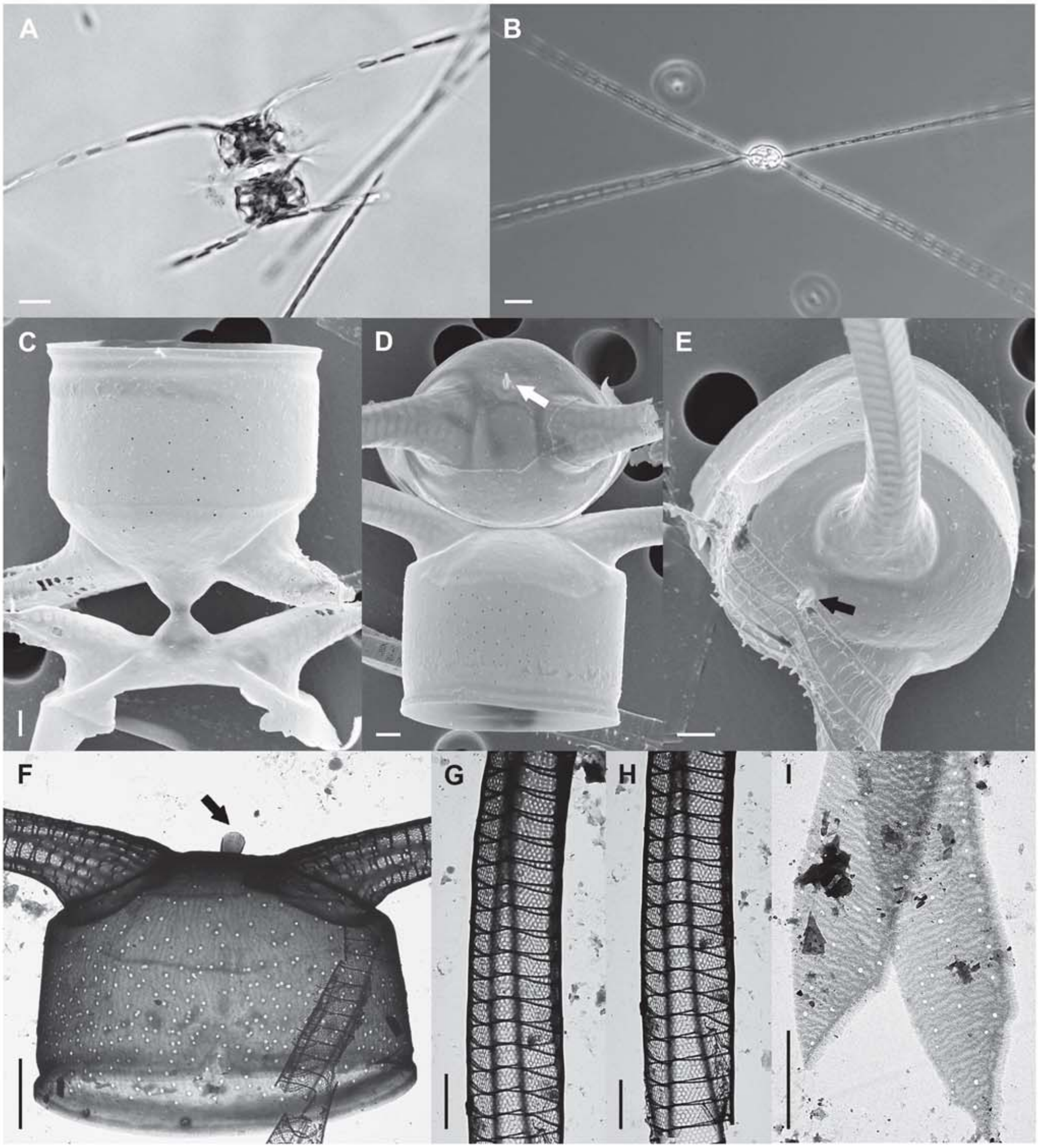

Fig. 11. *Chaetoceros rostratus,* strains Na1B3 (A, B) and Na1C3 (C-G). (A) Chain in broad girdle view; (B) single cell in valve view; (C) aperture between sibling valves, note the tube connecting the two adjacent valves; (D) apical and lateral view of two valves, the upper one has an eccentric tube-shaped rimoportula (arrowed); (E) valve with the eccentric tube-shaped rimoportula (arrowed); (F) lateral view of a valve with irregularly arranged pores and tube-shaped rimoportula (arrowed); (G-H) detail of setae; (I) girdle bands . A, B: LM; C-E: SEM; F-I: TEM. Scale bars = 10 μm (A), 20 μm (B), 1 μm (C-E, G, H), 2 μm (F, I).

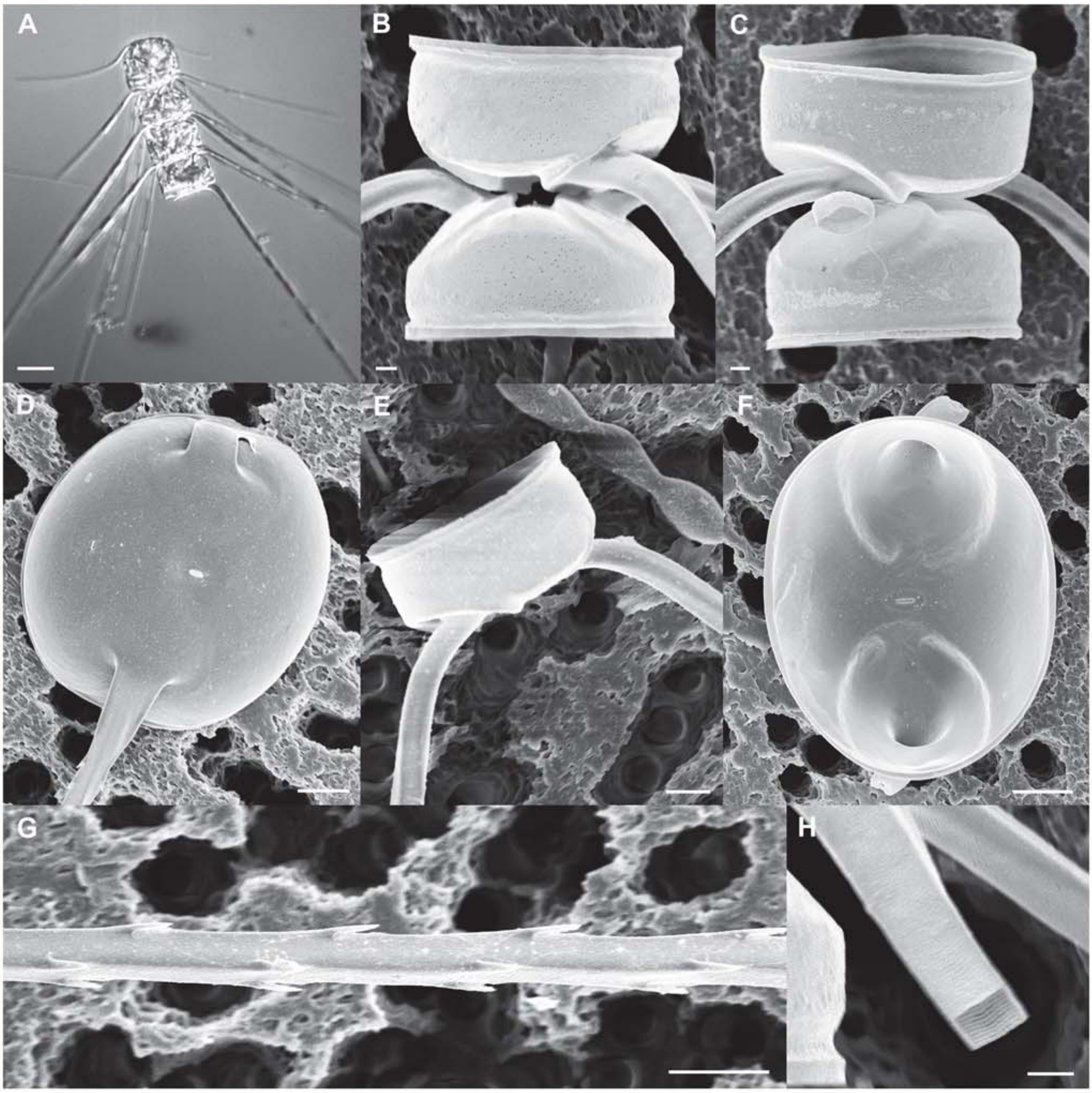

Fig. 12. *Chaetoceros* cf. *convolutus*, strains Ch5C3 (A-C, F-H) and Ch5C4 (D, E). (A) Chain in a broad girdle view; (B) intercalary valves ornamented with poroids; see the setae emerging from a depression in the posterior valve and from a round protuberance in the anterior valve; (C) lateral view of a terminal posterior valve; (D) intercalary valves; (E) internal view o a valve with slit-shaped rimoportula; (F) anterior terminal valve with tube-shaped rimoportula; (G) detail of the middle portion of a seta; (H) cross section of a seta. A: LM; B-H: SEM. Scale bars = 20 µm (A), 1 µm (B, H), 2 µm (C), 5 µm (D-G).

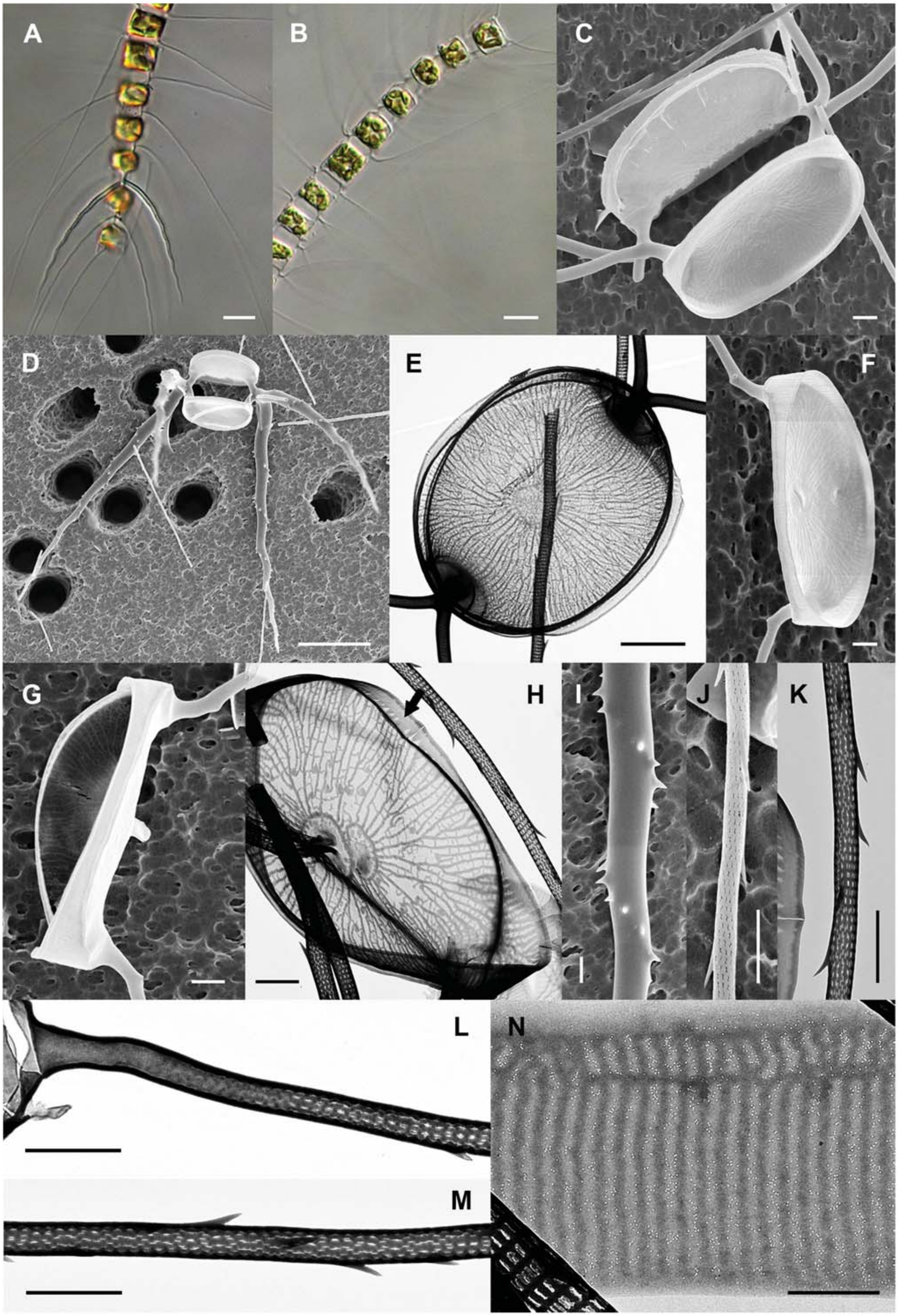
A
B
C
D
E
F
G
H
I
J
K
L
N
M

<<<<<

Fig. 13. *Chaetoceros contortus*, strain Ch8A1. (A) chain with thicker specialized intercalary setae; (B) colony in broad girdle view; (C) aperture between two adjacent intercalary valves; (D) two intercalary valves joined by thicker specialized setae; (E) intercalary valve; (F) internal view of a terminal valve with a slit-shaped rimoportula; (G) terminal valve with the tube-shaped rimoportula; (H) intercalary valve with the hyaline rim (arrowed); (I) detail of a thicker specialized seta with spirally arranged spines; (J, K) details of an intercalary seta with spines and poroids; (L) basal portion of an intercalary seta; (M) middle portion of an int ercalary seta ; (N) girdle band. A, B: LM; C, D, F, G, I, Jj: SEM; E, H, K-N: TEM. Scale bars = 10 µm (A, B, D), 2 µm (E), 1 µm (C, F-N).

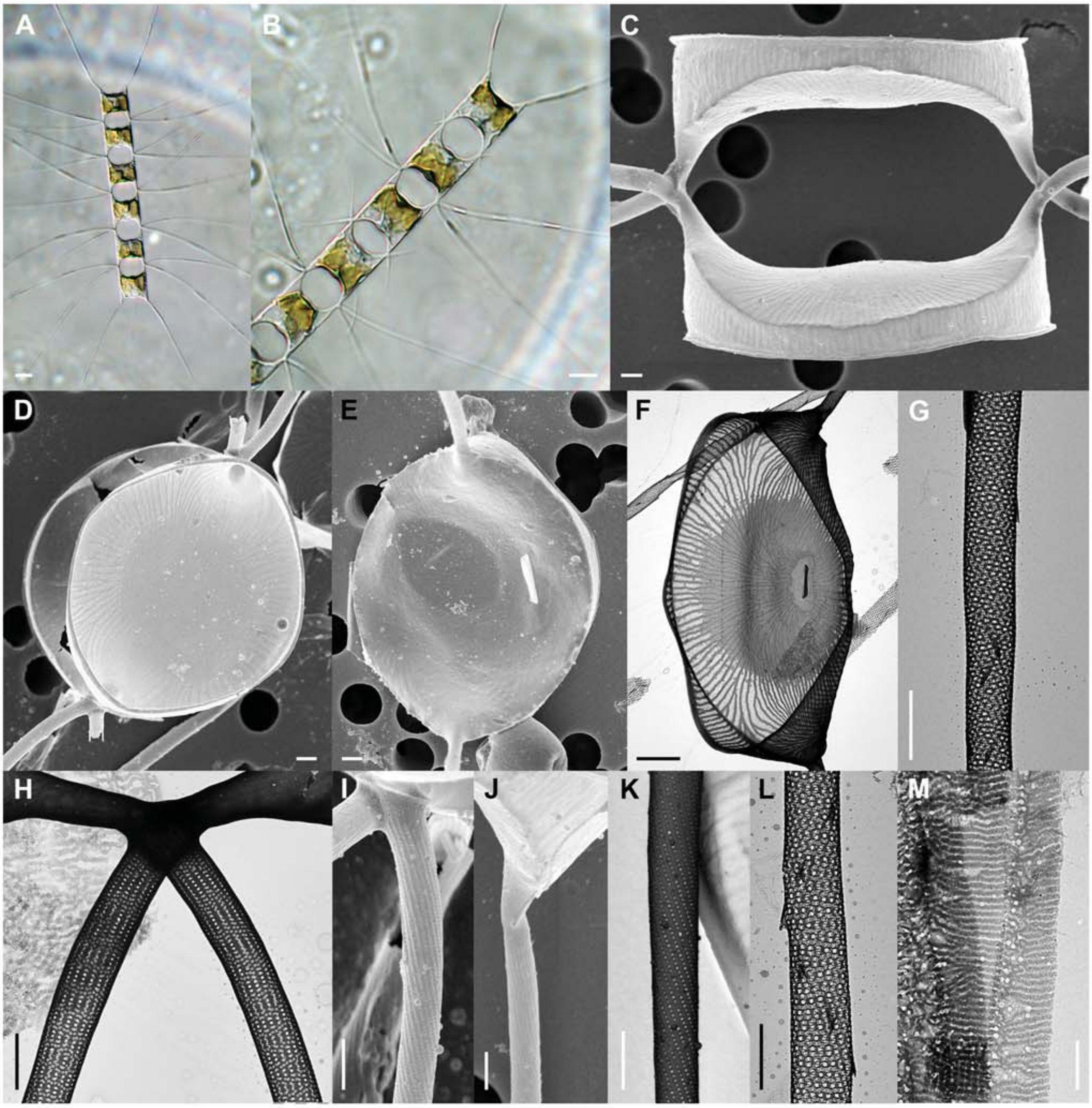

Fig. 14. *Chaetoceros brevis* 1, strains Na34B4 (A, B) and Na3C3 (C-M). (A-B) Chain in broad girdle view, with globules in the setae; (C) aperture between adjacent intercalary valves; (D) intercalary valves; (E) terminal valve with an elongated eccentric rimoportula; (F) terminal valve with a slit-shaped rimoportula; (G) detail of the middle part of an intercalary seta with spines and poroids; (H) detail of the junction between two intercalary setae; (I) detail of the basal portion of an intercalary seta; (J) detail of the base of a terminal seta; (K, L) details of the middle portion of terminal setae; (M) girdle bands. A, B: LM; C-E, I-K: SEM; F-H, L, M: TEM. Scale bars = 10 µm (A, B), 1 µm (C-M).

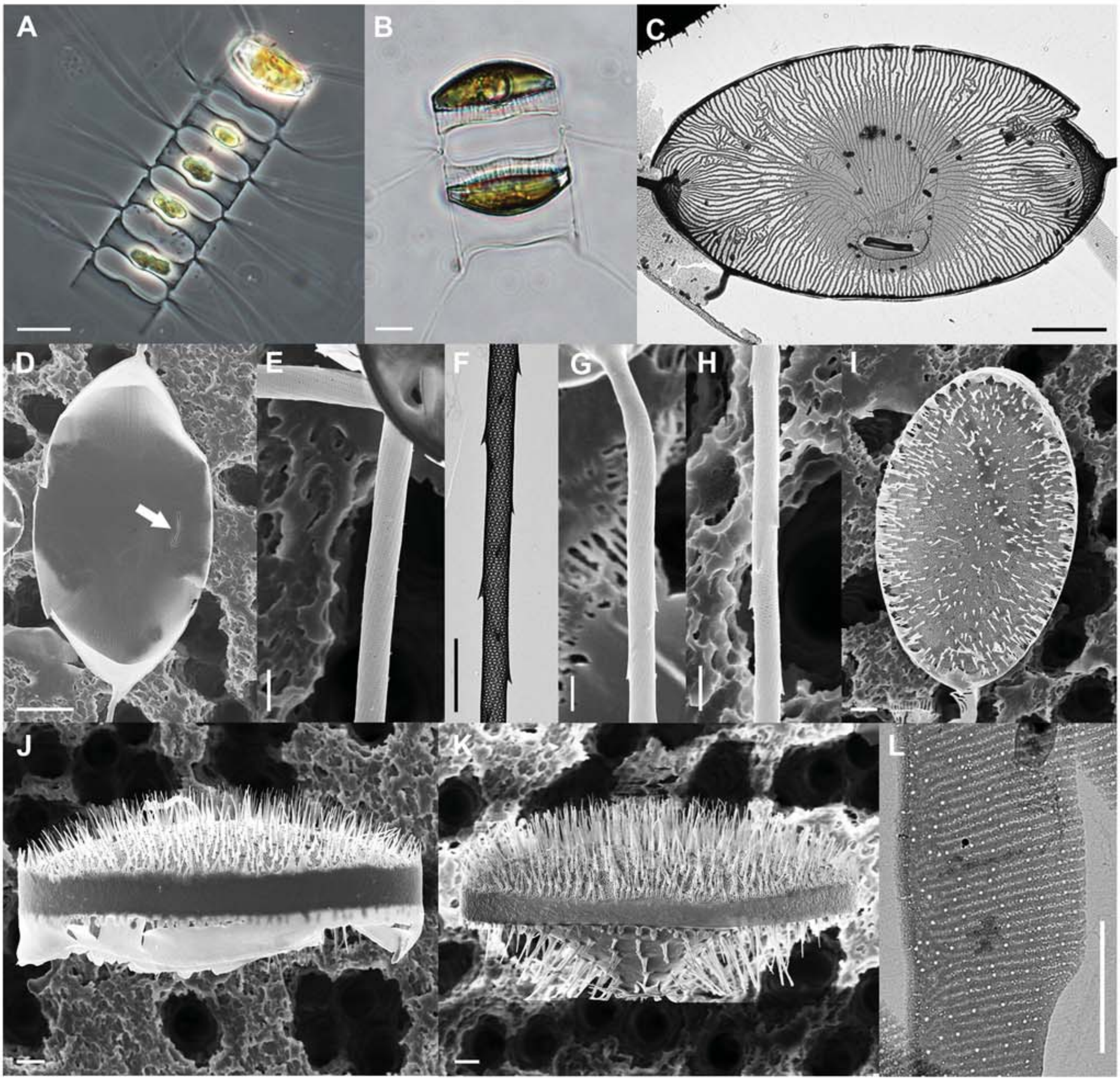

Fig. 15. *Chaetoceros brevis* 2, strain Na7C2. (A) Chain in broad girdle view; (B) spores; (C, D) internal view of terminal valve with an eccentric slit-shaped rimoportula (arrowed); (E) detail of the basal part of an intercalary seta; (F) detail of middle part of an intercalary seta; (G) detail of the basal part of a terminal seta; (H) detail of middle part of a terminal seta; (I) spore in valve view; (J-K) spores in girdle view; (L) girdle band. A, B: LM; D-E, G-K: SEM; C, F, L: TEM. Scale bars = 20 µm (A), 10 µm (B), 5 µm (C, D), 2 µm (F, H, I, J, K, L) and 1µm (E, G).

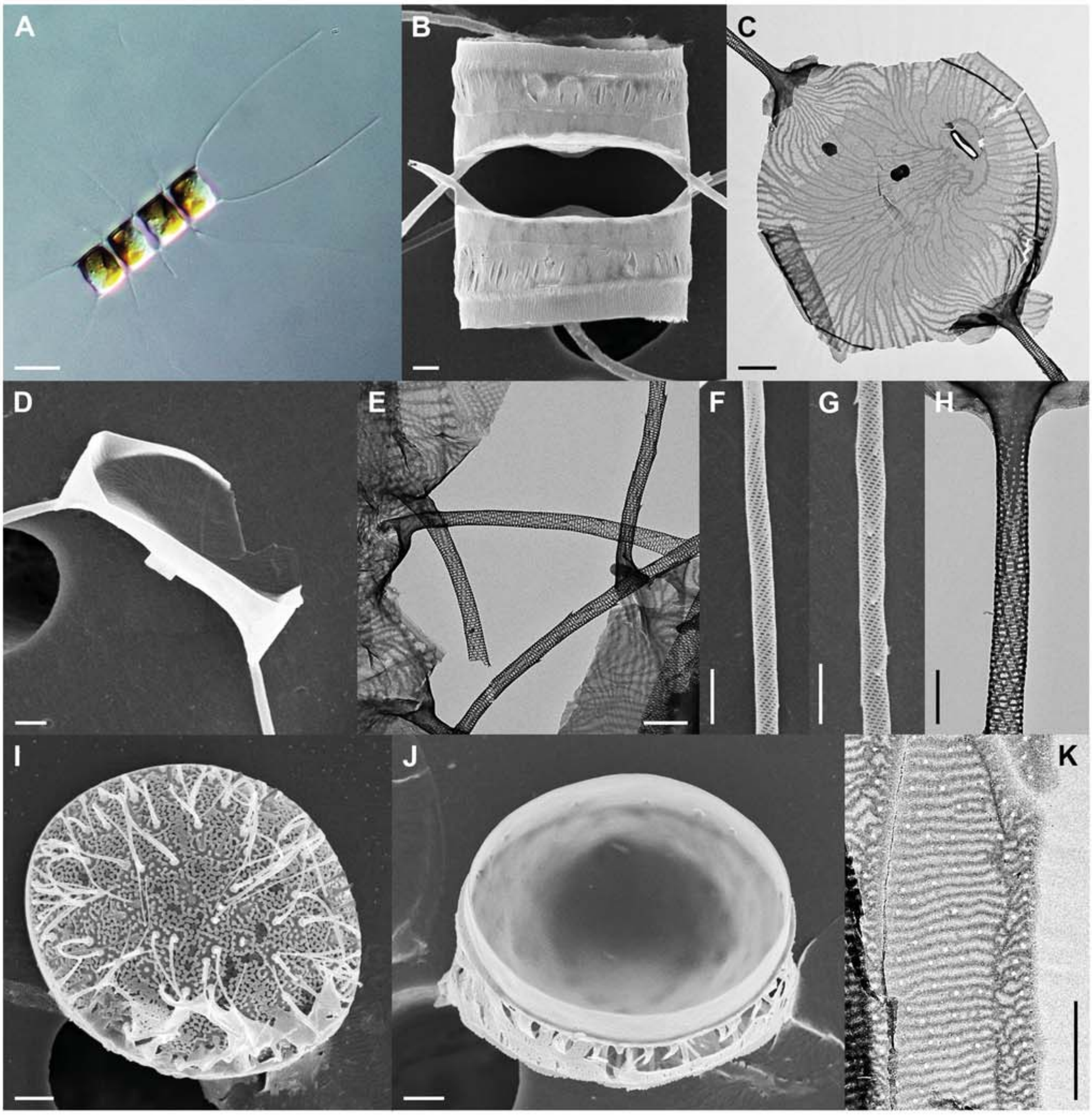

Fig. 16. *Chaetoceros brevis* 3, strain Ch9B3. (A) Chain in broad girdle view; (B) aperture between two adjacent intercalary valves; (C) terminal valve with eccentric slit shaped rimoportula; (D) terminal valve with tube-shaped rimoportula; (E) intercalary setae without spines in t heir basal portion, close to the junction (on the left), and with spines along their length; (F) detail of the basal part of an intercalary seta; (G) detail of the middle part of an intercalary seta; (H) detail of the basal part of a terminal seta; (I) spore in valve view; (J) internal view of the secondary valve of a spore; (K) girdle band. a: LM; B, D, F, G, I, J: SEM; C, E, H, L: TEM. Scale bars = 10 µm (A), 1 µm (B-K).

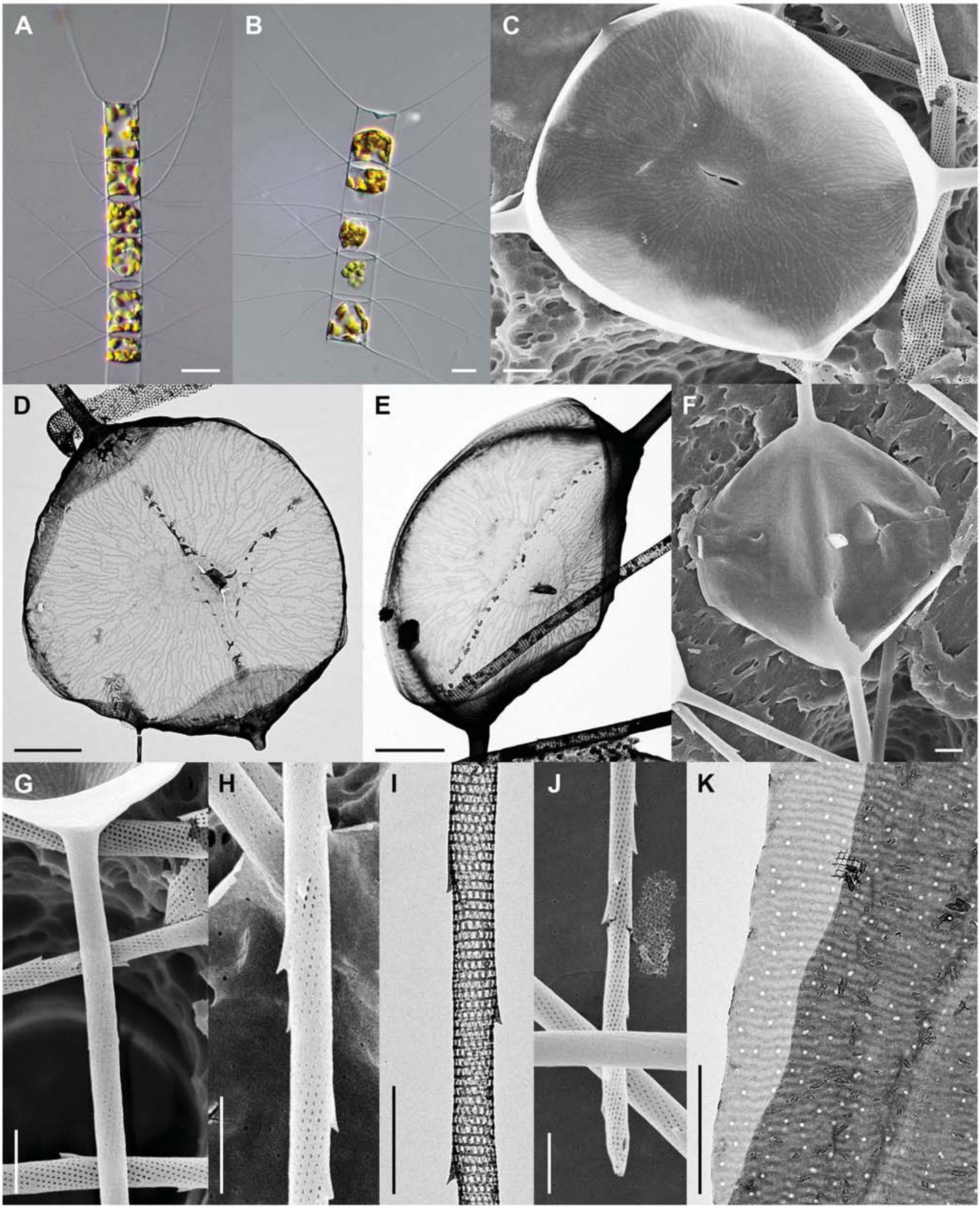

Fig. 17. *Chaetoceros teres*, strains Ch12B1 (A, B), Ch2C3 (C-K). (A, B) Chain in broad girdle view; (C) internal view of a terminal valve with a slit-shaped rimoportula; (D, E) terminal valve with tube-shaped rimoportula; (F) external view of a terminal valve with tube-shaped rimoportula; (G) detail of the basal part of an intercalary seta; (H I) detail of the middle part of an intercalary seta; (J) detail of the terminal part of an intercalary seta; (K) girdle bands. A, B: LM;C, F-H, J: SEM; F, E, I, K: TEM. Scale bars = 20 µm (A), 10 µm (B), 1 µm (C-K).

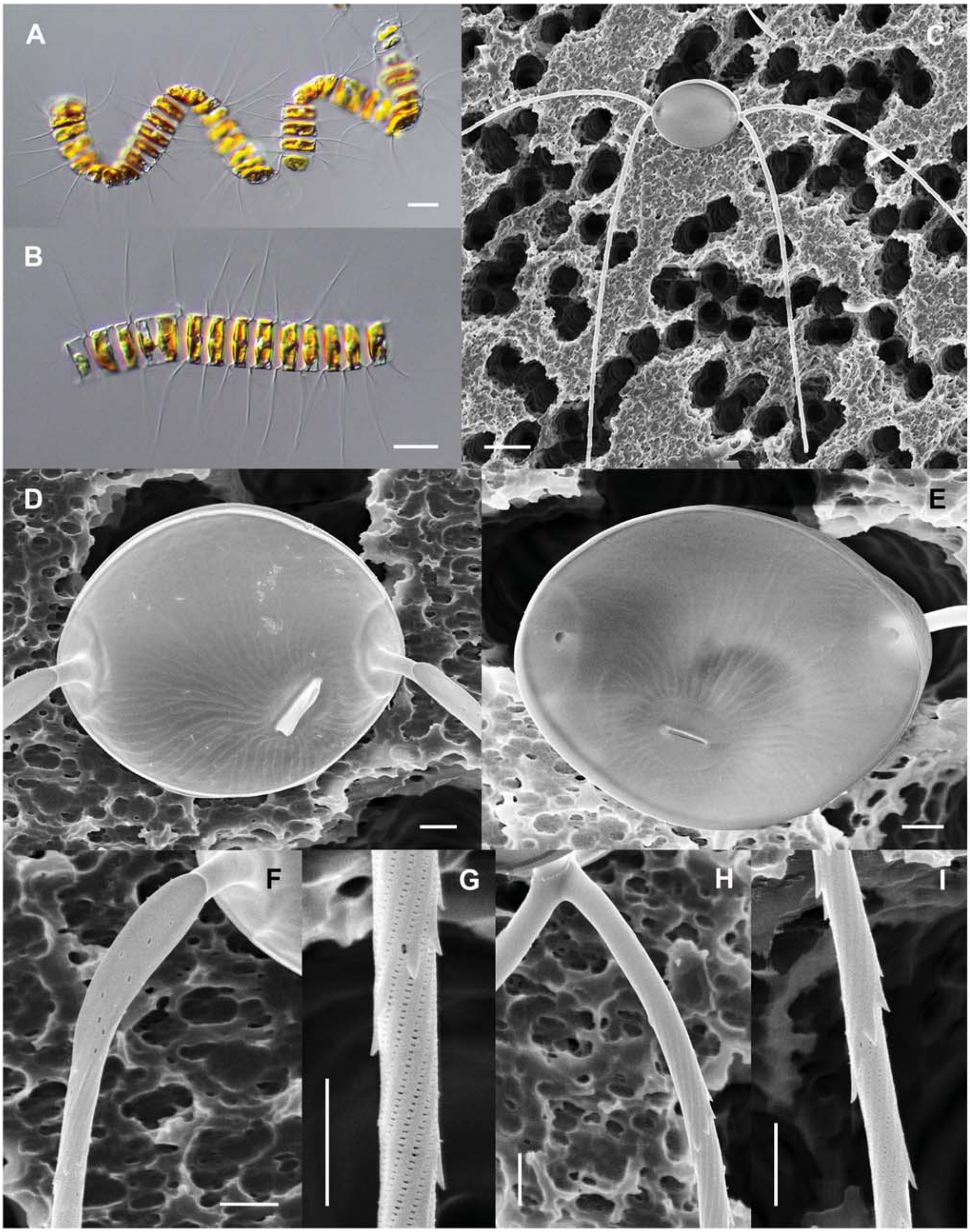

Fig. 18. *Chaetoceros debilis* 3, strains Ch13A4 (A-B) and Ch9A4 (C-I). (A) Spiralling chain; (B) chain in broad girdle view; (C) intercalary valve with setae joining two adjacent cells; (D) terminal valve with flattened tube-shaped rimoportula; (E) Internal view of terminal valve with slit-shaped rimoportula; (F) detail of the basal part of a terminal seta; (G) detail of the middle part of a terminal seta; (H) detail of the basal part of an intercalary seta; (I) detail of the middle part of an intercalary seta. A, B: LM; C-I: SEM. Scale bars = 20 μm (A, B), 10 μm (C), 1 μm (D-I).

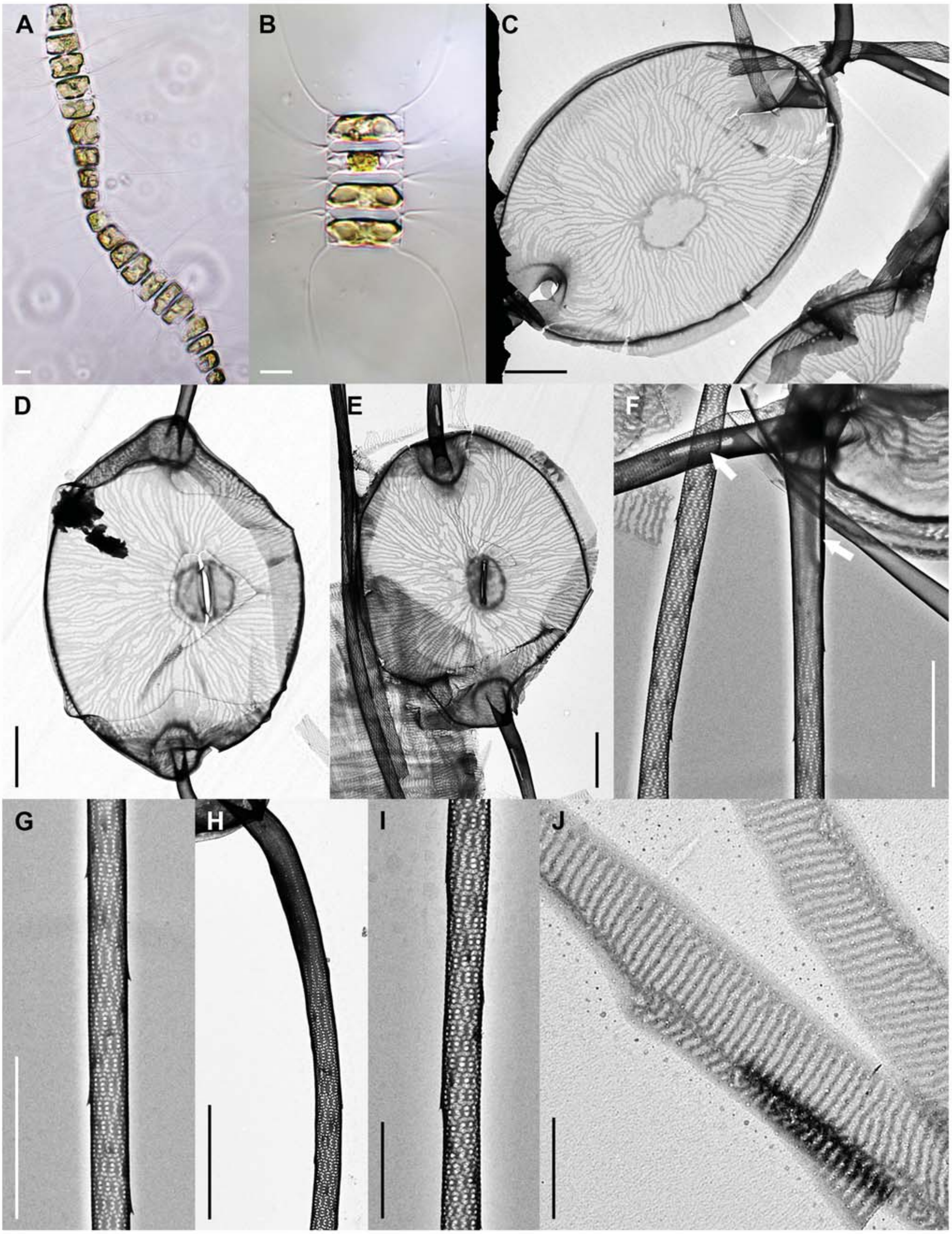

Fig. 19. *Chaetoceros tortissimus*, strains Na25B2 (A), Na25A3 (B) and Na34B3 (C-J). (a) Chain with a marked torsion; (B) colony in broad girdle view; (C) intercalary valve; the large pore in the basal part of seta is arrowed; (D, E) terminal valves with a slit-shaped rimoportula; (F) detail of the basal part of intercalary setae with large pores in their basal part (arrowed); (G) detail of the middle part of a terminal seta; (H) detail of the basal part of a terminal seta; (I) detail of the middle part of a terminal seta; (J) girdle bands. A, B: LM; C-J: TEM. Scale bars = 10 µm (A, B, 2 µm (C-H), 1 µm (I-J).

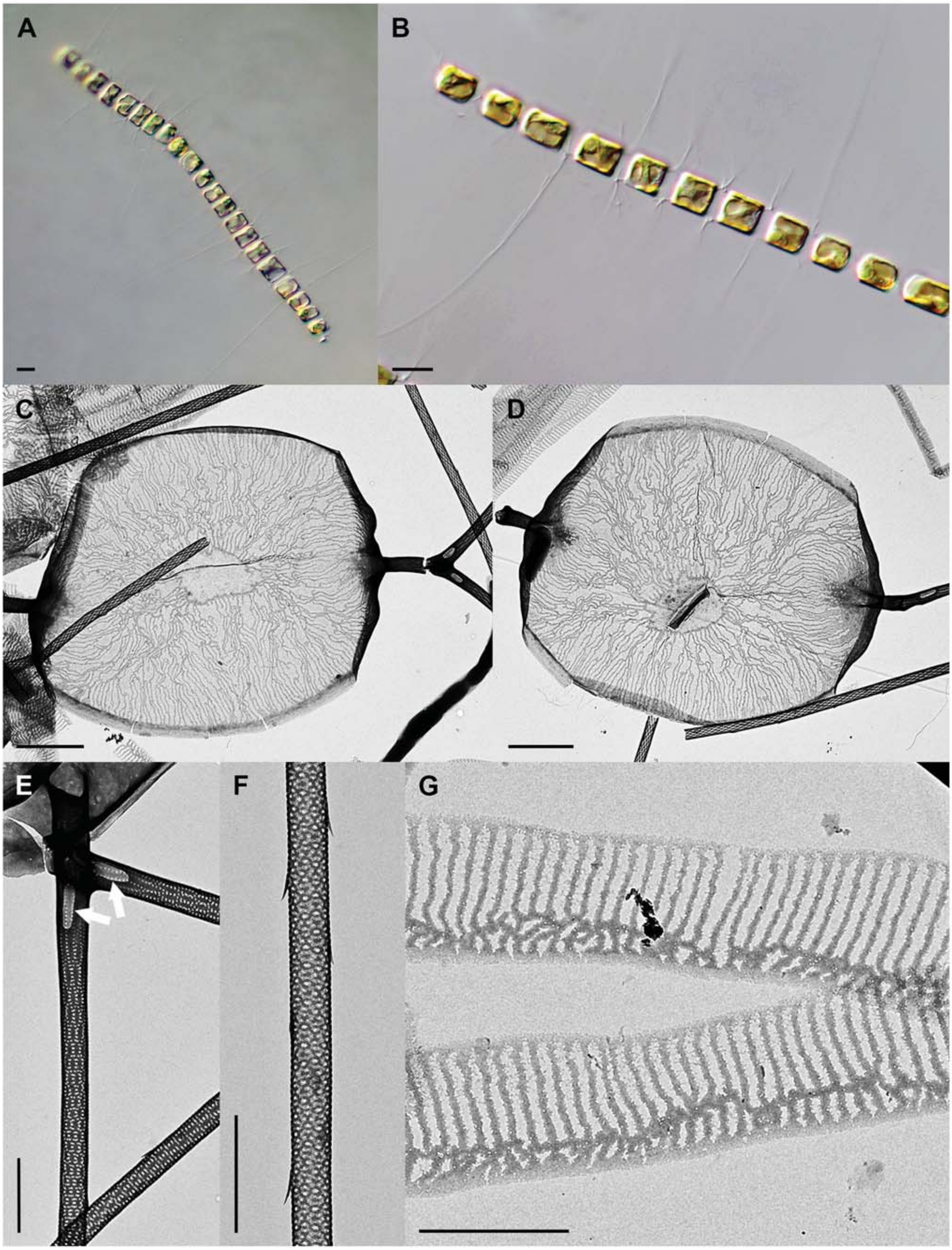

Fig. 20. *Chaetoceros* cf. *tortissimus*, strains Na28B3 (A), Na18C2 (B) and Na2A3 (C-G). (A, B) Colonies in broad girdle view; (C) intercalary valve with l arge pores on the base of setae arrowed; (D) external view of terminal valve with flattened tube-shaped rimoportula; (e) detail of the basal part of two intercalary setae at their junction with large pores arrowed; (F) detail of the middle part of an intercalary seta; (G) girdle bands. A, B: LM; C-G: TEM. Scale bars = 10 µm (A, B), 2 µm (C, D), 1 µm (E-G).

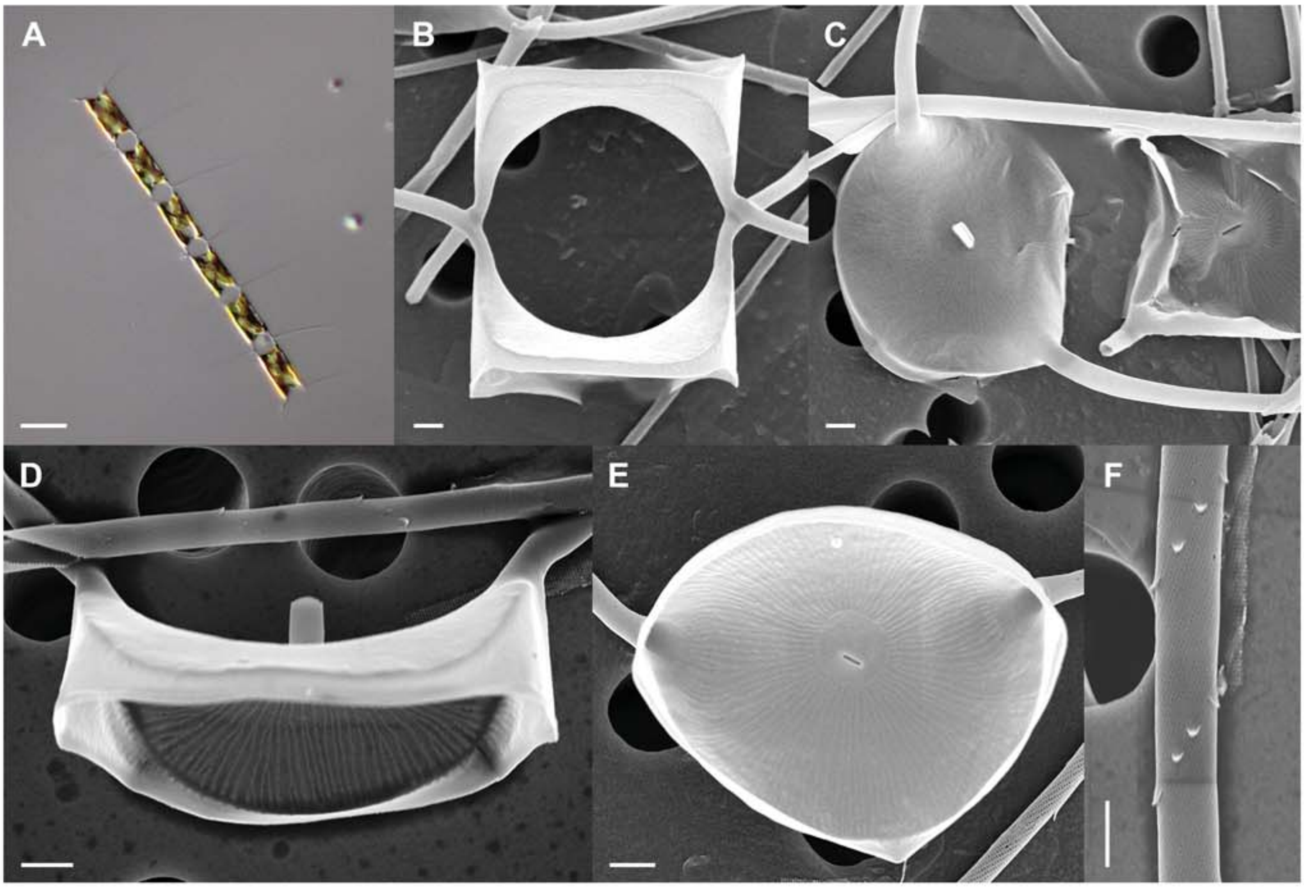

Fig. 21. *Chaetoceros curvisetus* 3, strain Na3C4. (A) Chain in broad girdle view; (B) aperture between two valves of adjacent intercalary cells; (C) external view of terminal valve with a tube-shaped rimoportula; (D) lateral view of terminal valve with a tube-shaped rimoportula; (E) internal view of terminal valve with a slit-shaped rimoportula; (F) detail of the middle part of an intercalary seta. A: LM; B-F: SEM. Scale bars = 20 µm (A), 1 µm (B-F).

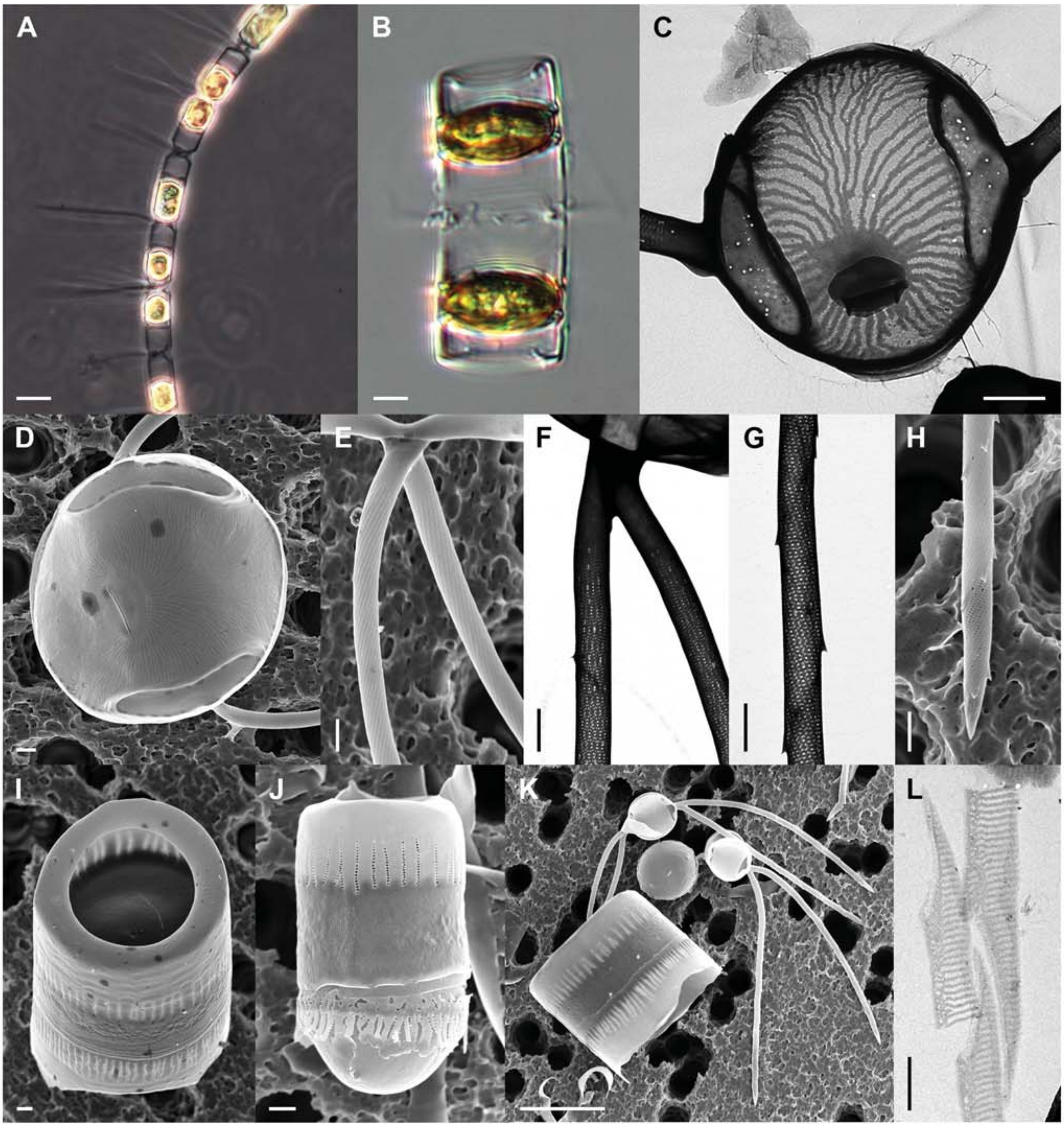

Fig. 22. *Chaetoceros pseudocurvisetus*, strain Na13C4. (A) Chain in narrow girdle view with spores within the frustules of the parental vegetative cells; (B) spores within the frustule in broad girdle view; (C) intercalary valve; (D) internal view of intercalary valve with slit-shaped rimoportula; (E, F) basal part of intercalary setae; (G) intermediate part of an intercalary seta; (H) terminal part of an intercalary seta; (I) spore; (J, K) lateral view of spores; (L) girdle bands. A, B LM; D, E, H, I-K: SEM; C, F G, L: TEM. Scale bars = 10 µm (A, K); 5 µm (B); 1 µm (C-J, L).

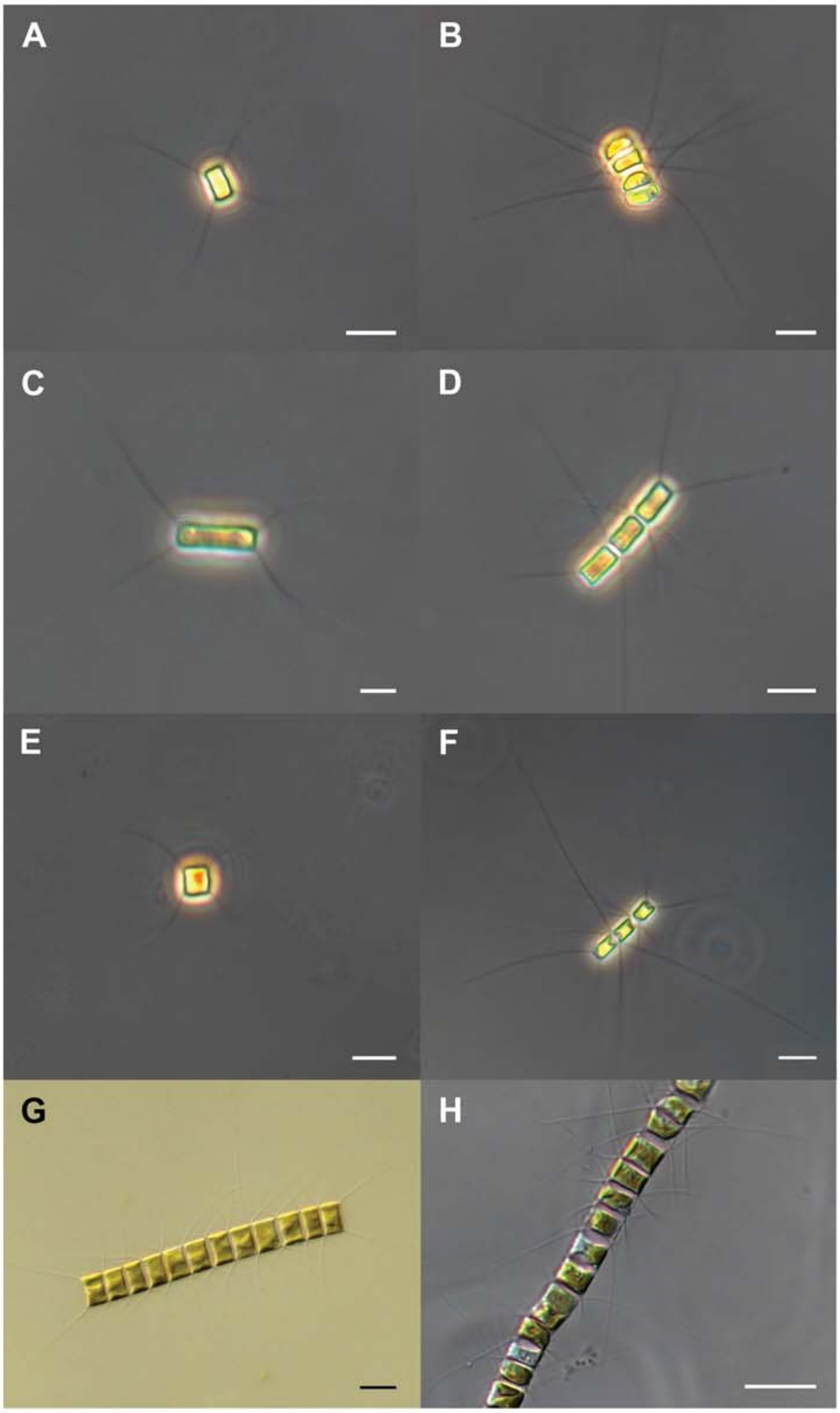

Fig. 23. *Chaetoceros tenuissimus*, strains GB1A (A, B), GB2A (C, D), EA4 (E, F), Na44A1 (G, H). (A) Single cell; (B) short chain; (C) elongated single cell; (D) short chain; (E) single cell; (F) short chain; (G, H) longer chains. A-H: LM. Scale bars = 10 µm (A, B, D-H); 5 µm (C).

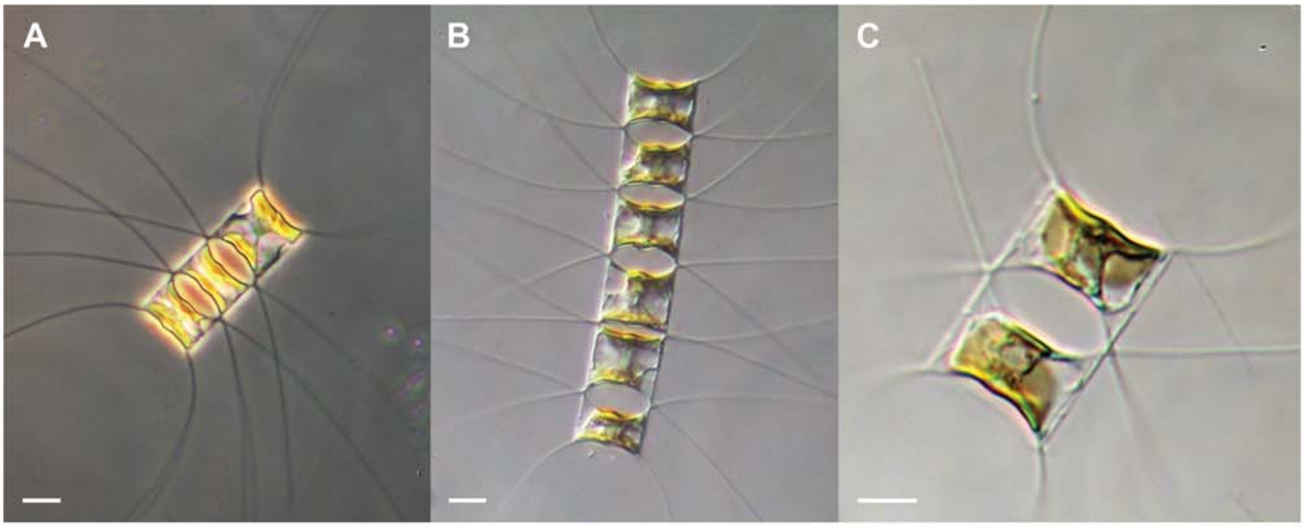

Fig. 24. *Chaetoceros* sp. clade NA13C2. (A, B) Chain in broad girdle view; (C) two-celled chain. A-C: LM. Scale bars = 20 µm (A-C).

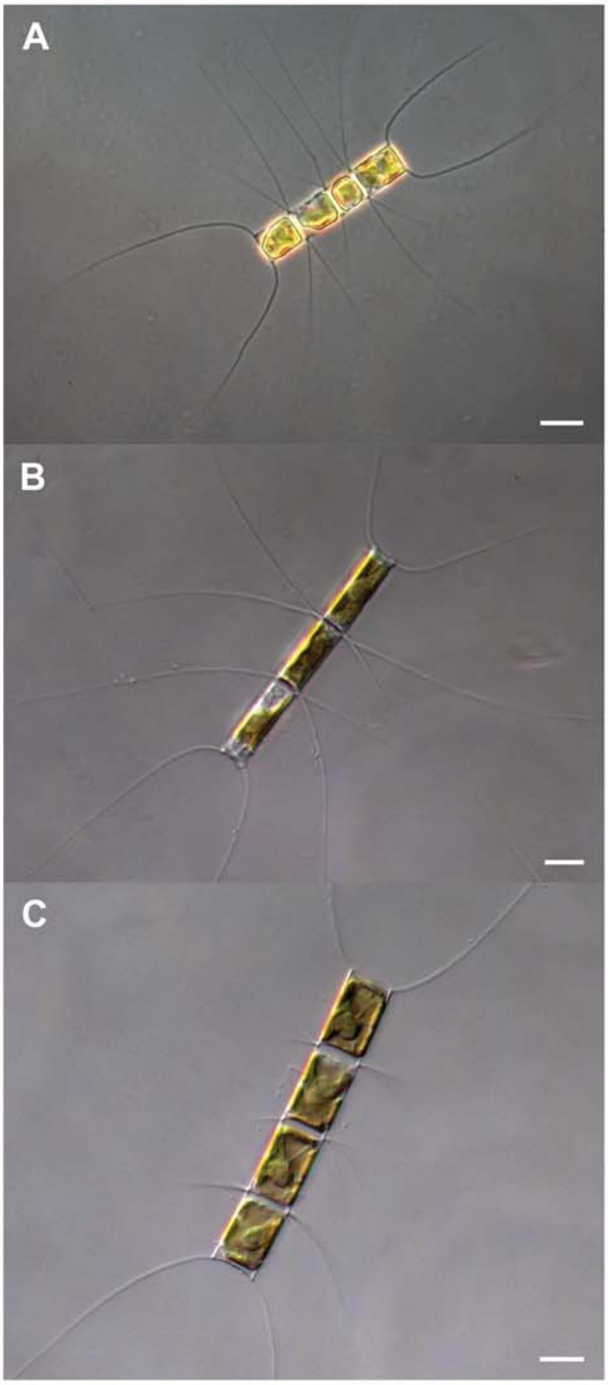

Fig. 25. *Chaetoceros* sp. clade Na12A3, strains NA9A3 (A), Na12A3 (B), Na12A2 (C). (A-C) Short chains of cells of different size. A-C: LM. Scale bars = 20 µm (A), 10 µm (B, C).

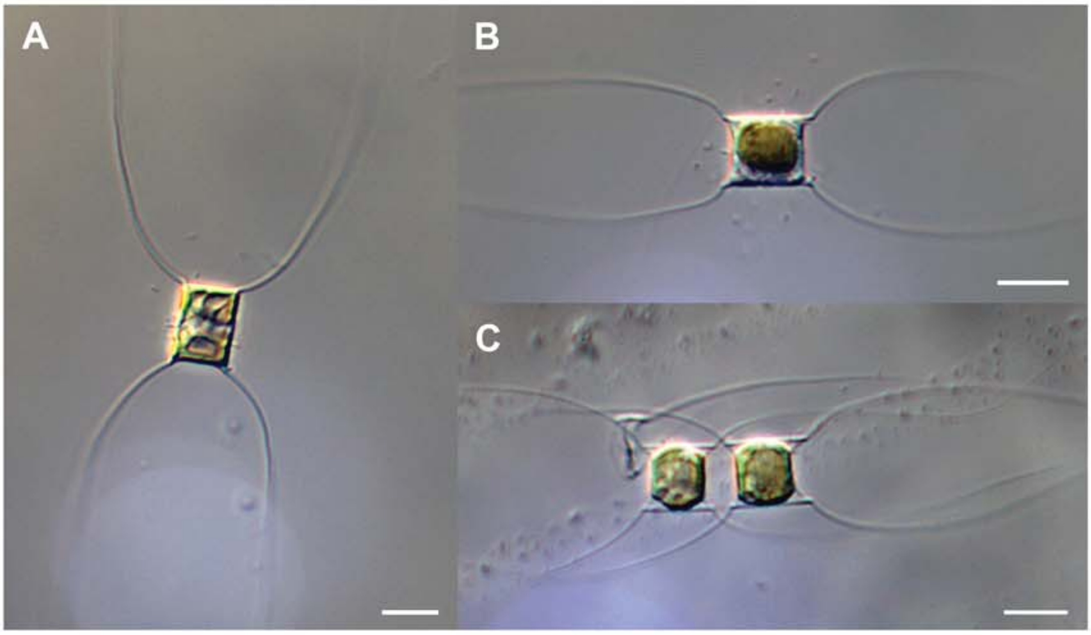

Fig. 26. *Chaetoceros* sp. clade Na17B2. (A, B) Single cells; (C) recently divided cells. A-C: LM. Scale bars = 10 µm (A-C).

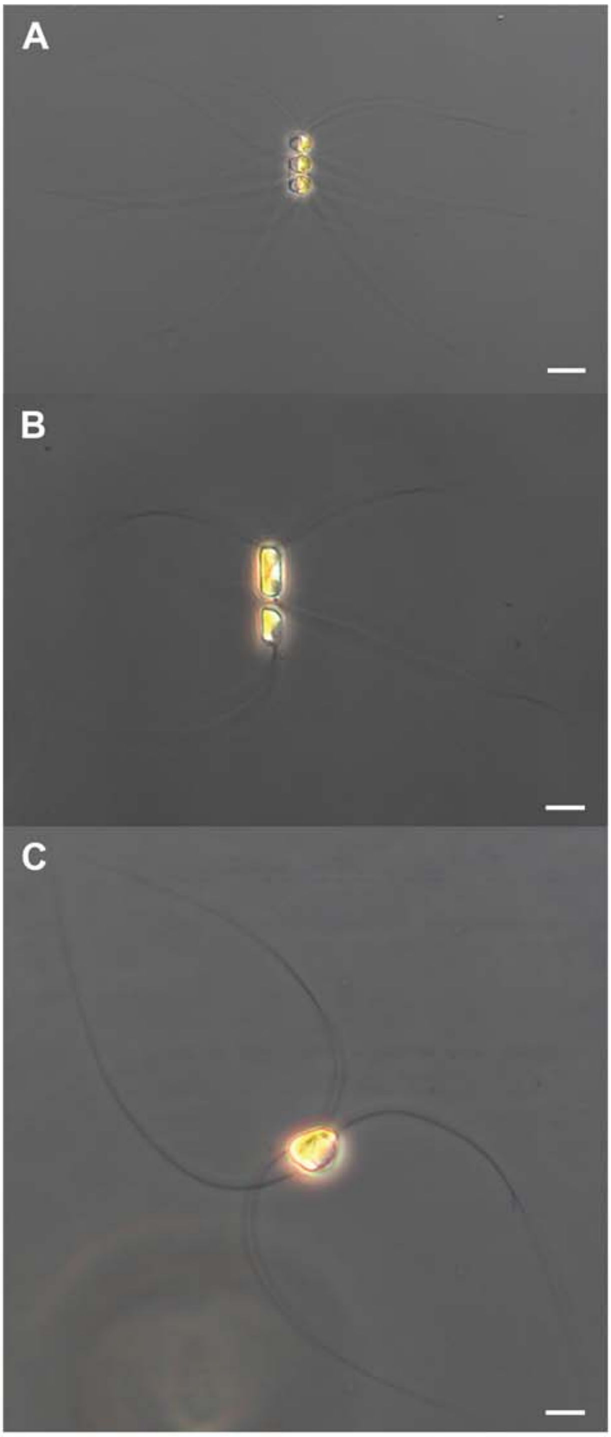

Fig. 27. *Chaetoceros circinalis, strain Na15C2*. (A, B) Short chains; (C) single cell. A-C: LM. Scale bars = 20 µm (A), 10 µm (B, C).

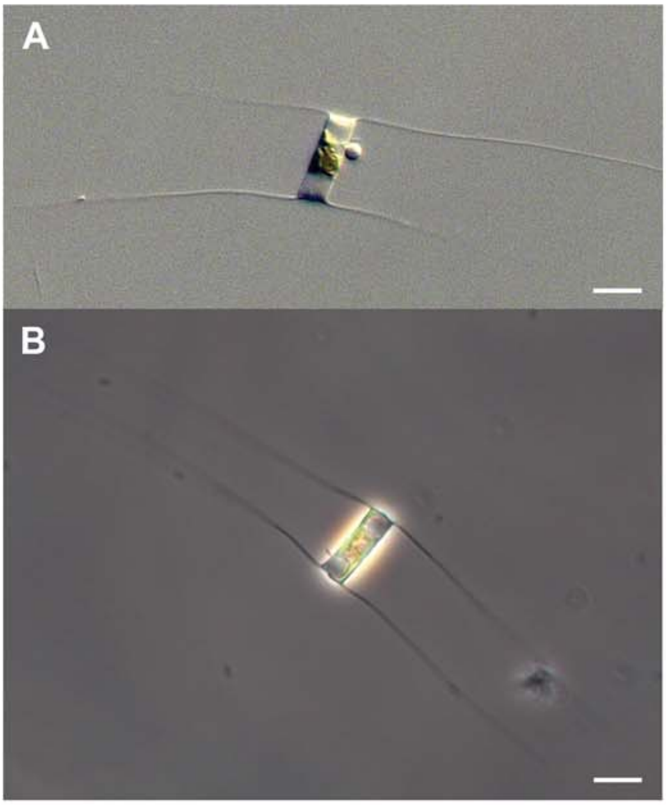

Fig. 28. *Chaetoceros* sp. clade CDP22, strain CDP22. (A, B) LM of single cells. Scale bars = 5 µm (A, B).

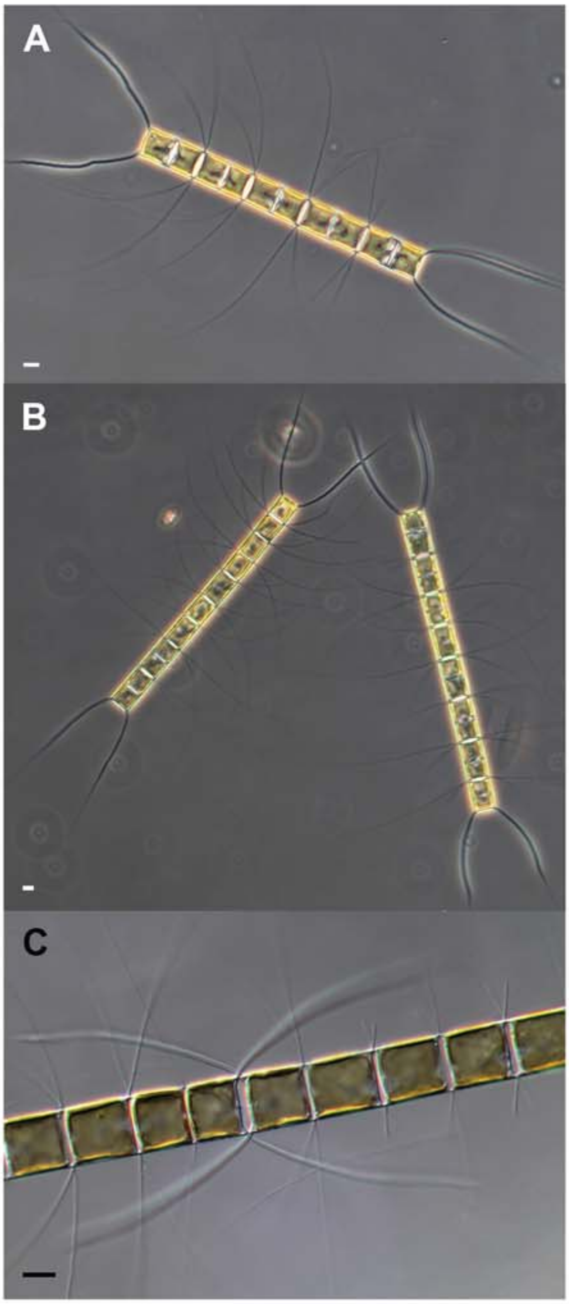

Fig. 29. *Chaetoceros affinis*, strain Na27C2. (A, B) Chains with distinct terminal setae; (C) detail of a chain. A-C: LM. Scale bars = 10 µm (A-C).

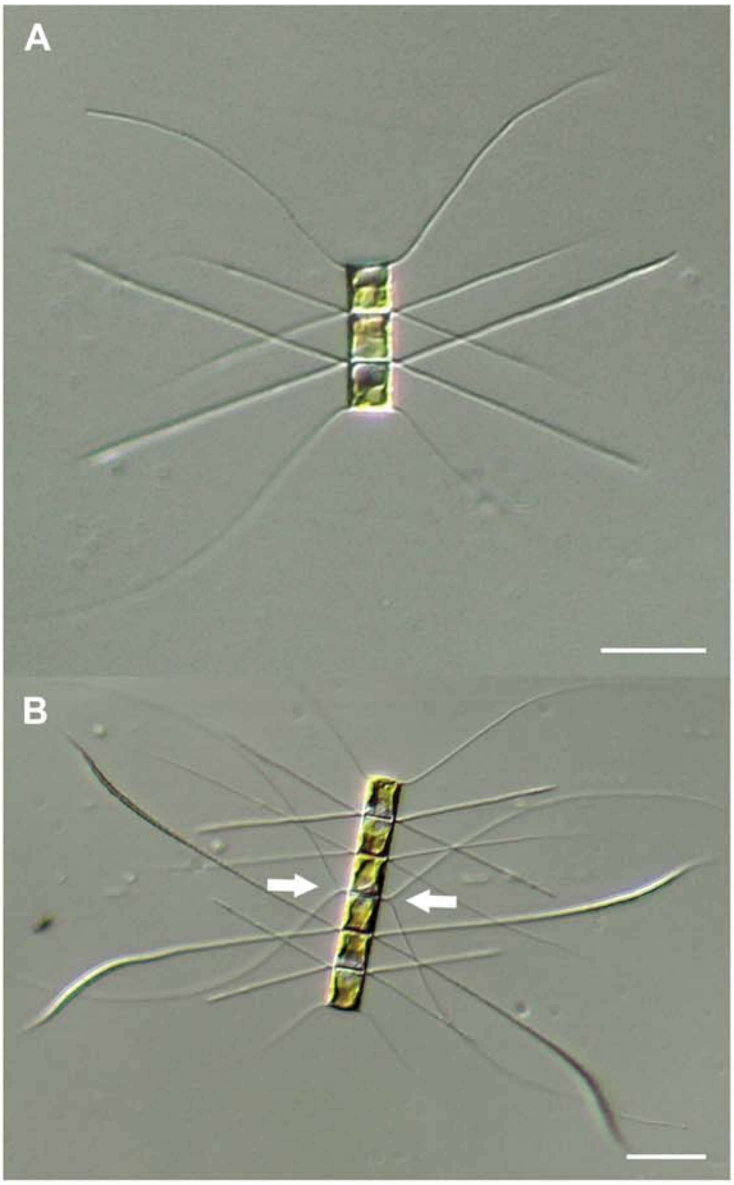

Fig. 30. *Chaetoceros diversus* 1, strain Na5B2. (A) Chain in broad girdle view; (B) chain in broad girdle view with thicker intercalary specialized setae; note that the chain is separating (new terminal setae are arrowed). A, B = LM. Scale bars = 20 µm (A, B).

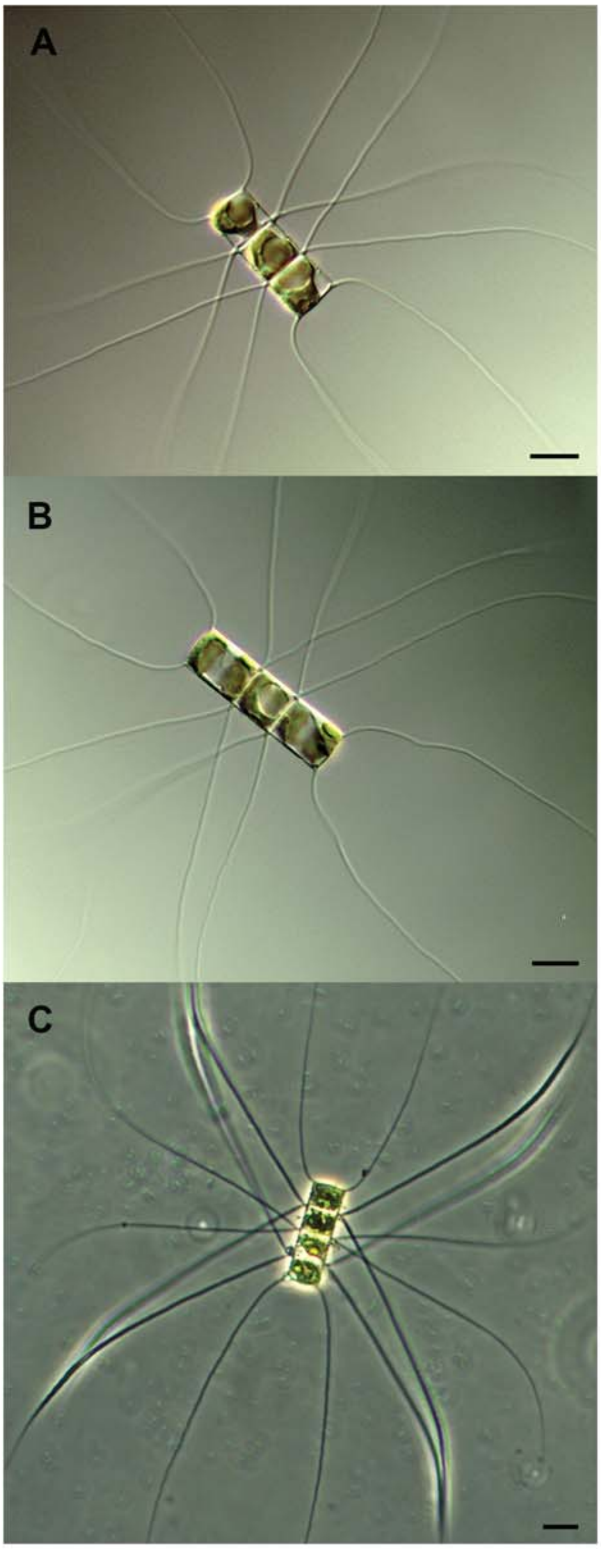

Fig. 31. *Chaetoceros diversus* 2, strain Na55C1 (A, B); natural sample (C). (A, B) Chains in broad girdle view; only the normal intercalary setae are present; (C) chain with thicker intercalary specialized setae. A-C: LM. Scale bars = 10 µm (A-C).

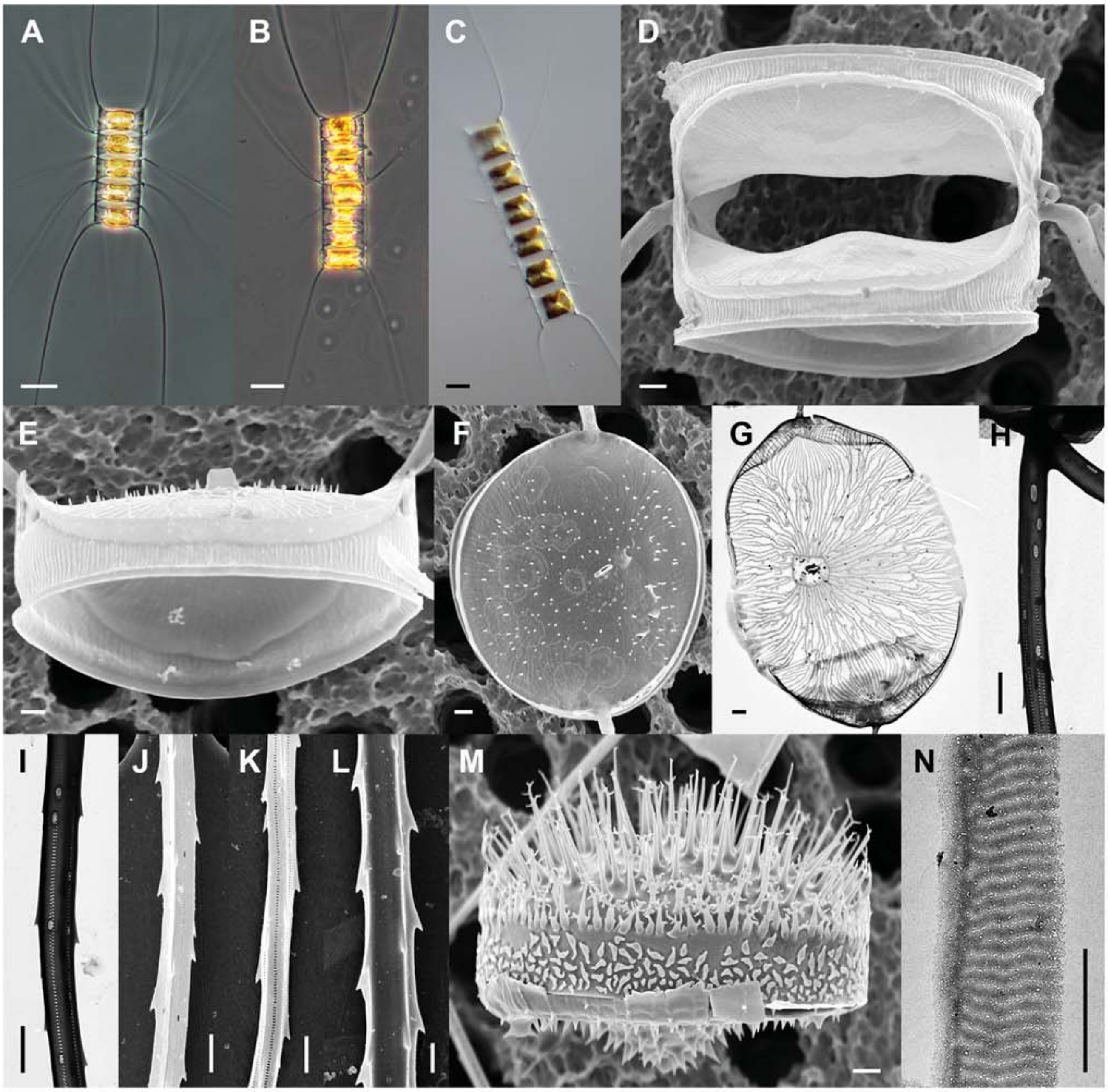

Fig. 32. *Chaetoceros constrictus*, strains Ch3C3 (A, B, E-L,N), Ch12C1 (D, M) and Na42C3 (C). (A-C) Chains in broad girdle view; (D) aperture between intercalary adjacent valves; (E) lateral view of terminal valve with tube-like shaped rimoportula, note the presence of spines on valve face; (F) external view of terminal valve with tube-shaped rimoportula; (G) internal view of terminal valve with slit- shaped rimoportula; (H-I) details of intercalary setae; (J-K) details of terminal setae; (L) detail of a thick terminal seta; (M) spore, lateral view; (N) girdle band. A-C: LM; D-F, J-M: SEM; G-I, N: TEM. Scale bars = 20 µm (A-C), 1 µm (C-N).

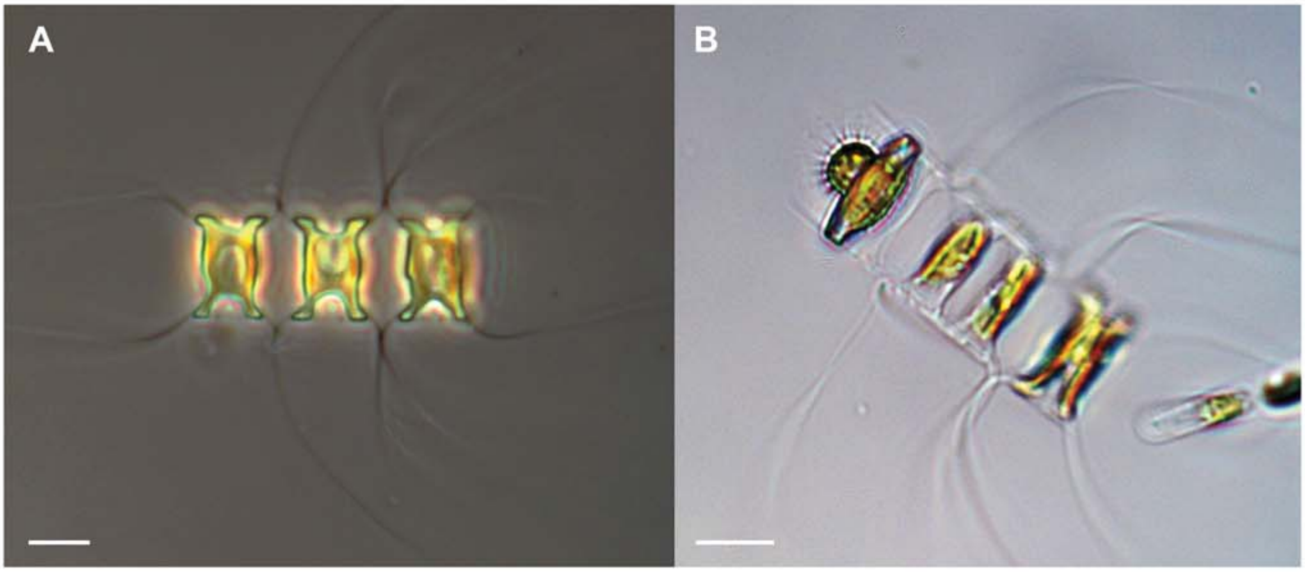

Fig. 33. *Chaetoceros seiracanthus*, strain EB3 small. (A) Chain in broad girdle view; (B) chain in broad girdle view with a spore in the frustule at the top. A, B: LM. Scale bars = 10 µm (A, B).

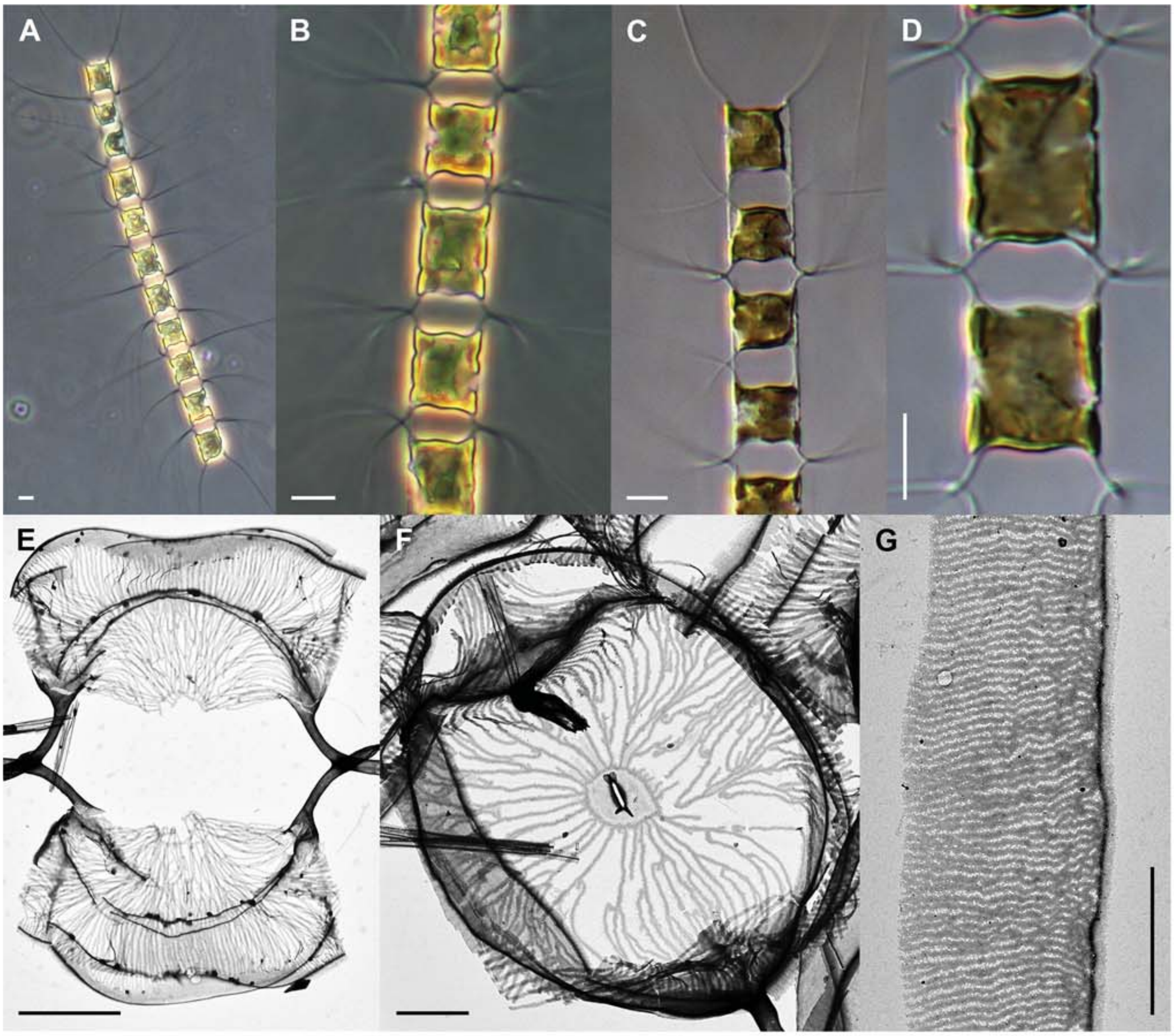

Fig. 34. *Chaetoceros* sp. clade Na13C1. (A-D) Chains in broad girdle view; (E) aperture between two adjacent intercalary valves; (F) internal view of a terminal valve with a slit-shaped rimoportula; (G) girdle band. A-D: LM; E-G: TEM. Scale bars = 20 µm (A-D), 5 µm (E) 2 µm (F, G).

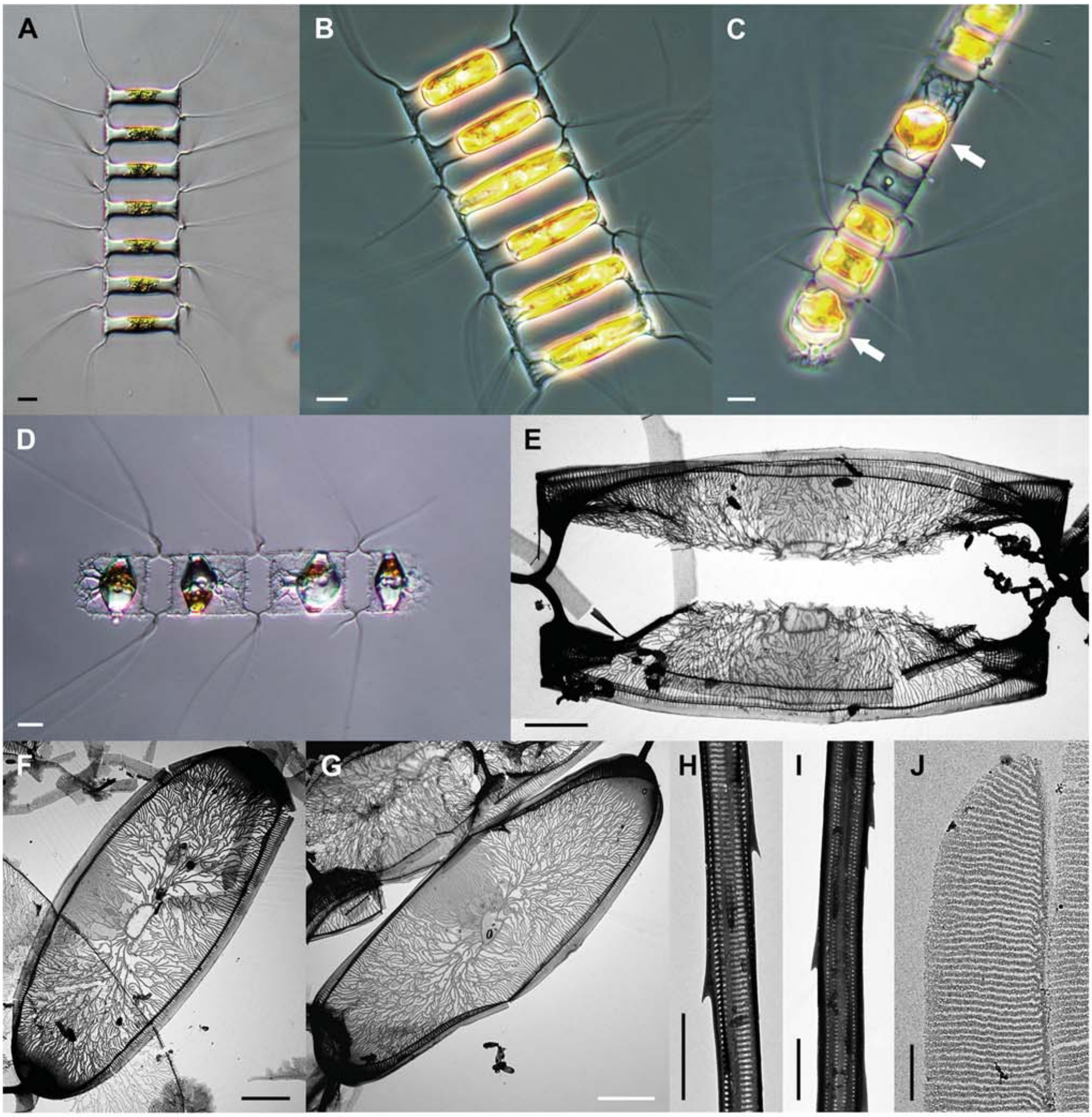

Fig. 35. *Chaetoceros diadema* 2, strain Ch5C1 (A, B, D-J), strain Ch1A3 (C). (A, B) Chains in broadgirdle view; (C) chains with spores (arrowed) and vegetative cells; (D) chain with spores; (E) aperture between two adjacent intercalary valves; (F) intercalary valve; (G) terminal valve with a small slit-shaped rimoportula; (H) detail of a distal portion of a terminal seta; (I) detail of an intercalary seta; (J) girdle band. A-D: LM; E-J: TEM. Scale bars = 10 µm (A-D), 5 µm (F, G), 1 µm (E, H-J).

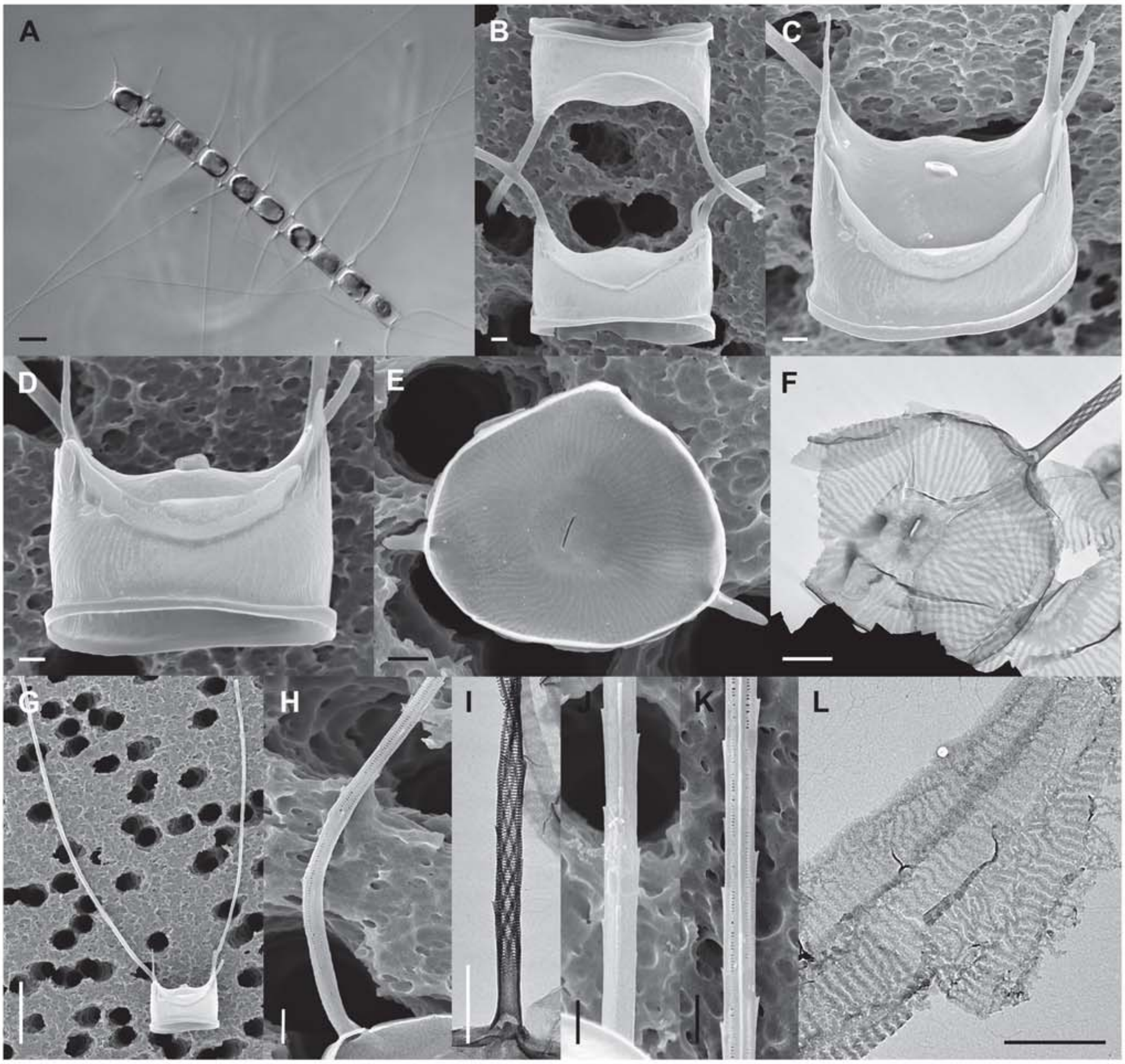

Fig. 36. *Chaetoceros* sp. clade Va7D2, strain Na43A4. (A) Chain in broad girdle view; (B) aperture between two adjacent intercalary valves; (C) terminal valve with tube-shaped rimoportula; (D) lateral view of terminal valve with tube-shaped rimoportula; (E, F) internal view of terminal valves with slit-shaped rimoportula; (G) terminal valve with long terminal setae; (H, I) basal portion of terminal setae; (J) basal part of intercalary seta; (K) middle part of intercalary seta; (L) girdle bands. A: LM; B-E, H, J, K: SEM; E, I, L: TEM. Scale bars = 10 µm (A, G), 1 µm (B-F, H-L).

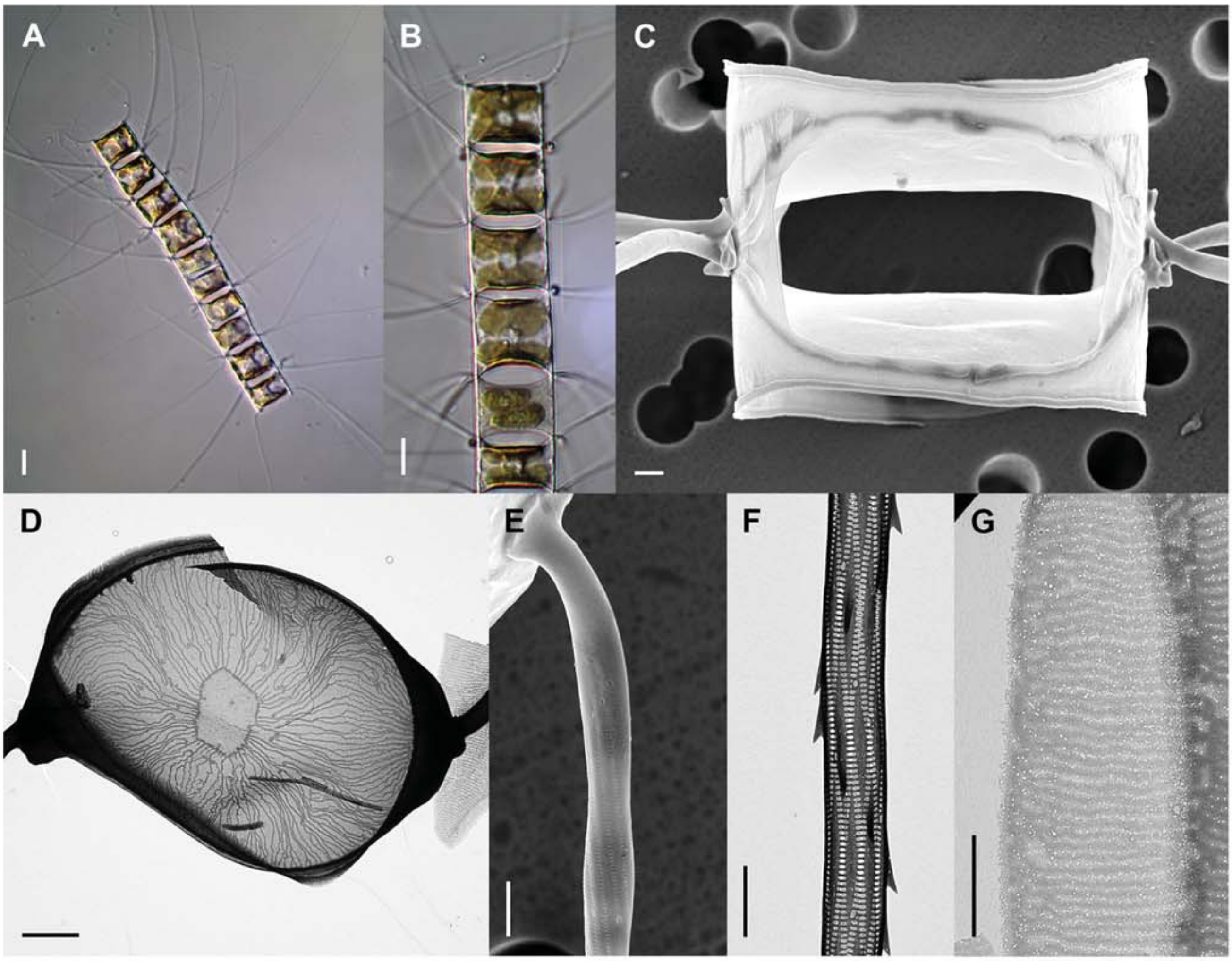

Fig. 37. *Chaetoceros* sp. clade Na28A1, strains Na26C1 (A,B) and Na28A1 (C-G). (A, B) Chains in broad girdle view; (C) aperture between two adjacent intercalary valves; (D) intercalary valve; (e) detail of the basal part of terminal seta; (F) detail of the middle part of terminal seta; (G) girdle band. A, B: LM; C, E: SEM; D, F, G: TEM. Scale bars = 10 µm (A, B), 2 µm (D, G), 1 µm (C, E, F).

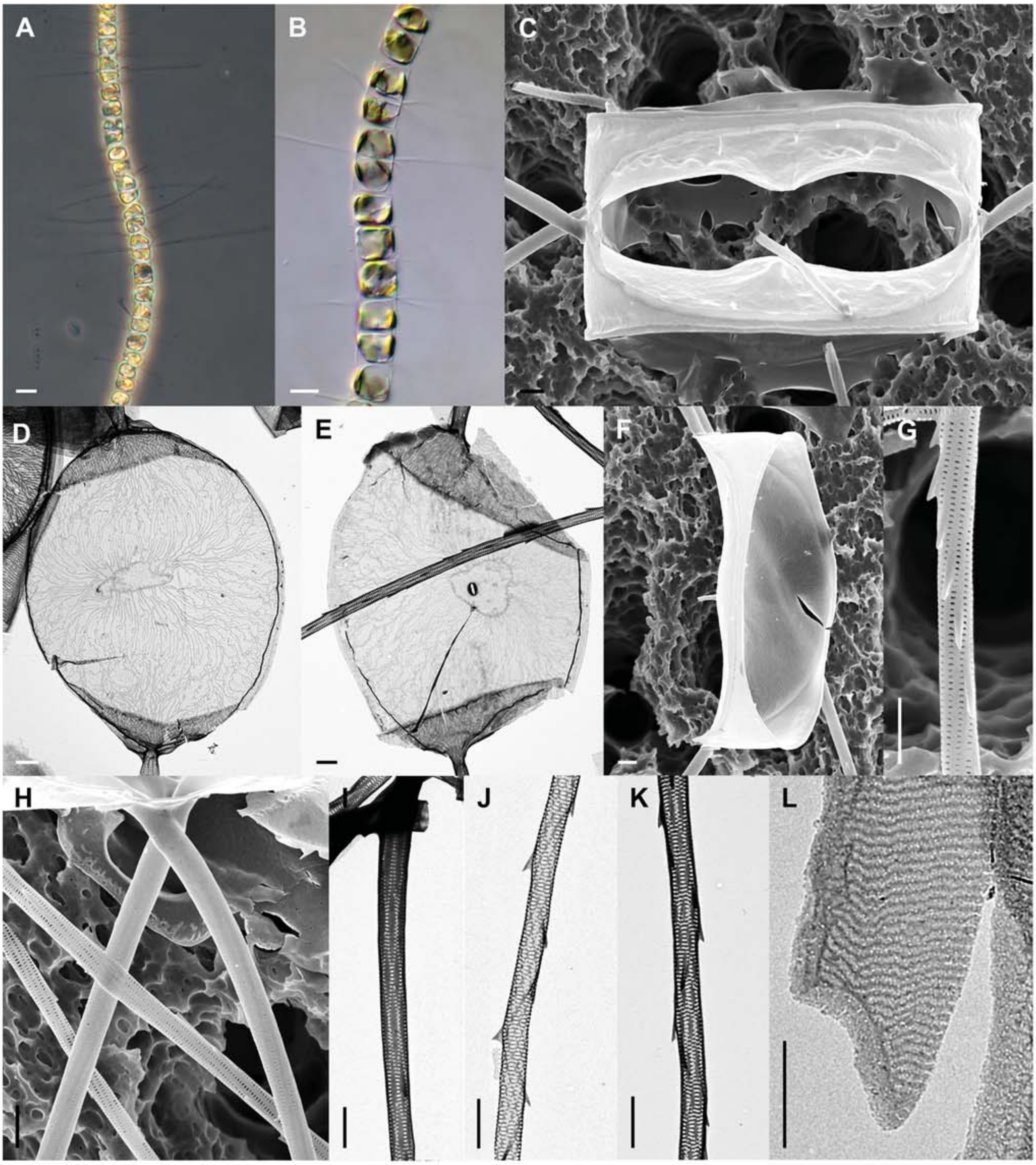

Fig. 38. *Chaetoceros* cf. *vixvisibilis*, strain Na25C3 (A, B), Na18C2 (C-L). (A, B) Chains in broad girdle view; (C) aperture between two **a**djacent intercalary valves; (D) intercalary valve; (E) terminal valve with slit-shaped rimoportula; (F) lateral view of a terminal valve with a tube-shaped rimoportula; (G-K) intercalary setae; (L) girdle band. A, B: LM; C, F-H: SEM; D, E, J-L: TEM. Scale bars = 10 µm (A, B), 1 µm (C-L).

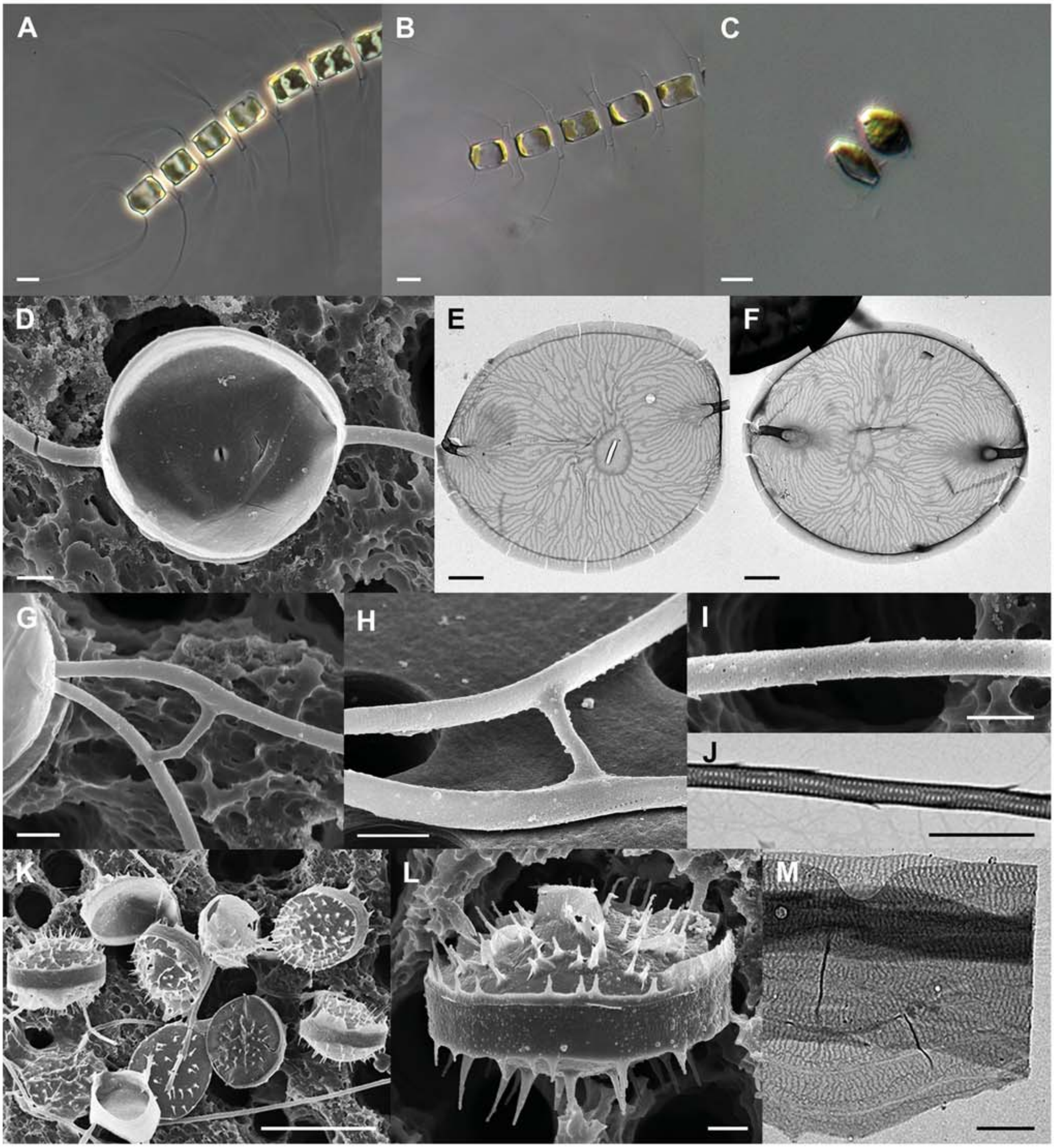

Fig. 39. *Chaetoceros anastomosans*, strain Na14A1 (A, C-G, I-M), Na14C3 (B), Na14C4 (H). (A, B) Chains in broad girdle view; (C) spores; (D) internal view of a terminal valve with slit-shaped rimoportula; (E) terminal valve; (F) intercalary valve; (G) basal part of intercalary setae of adjacent cells joined together by a bridge; (H) detail of the bridge; (I, J) detail of intercalary setae; (K) spores in different view; (L) spore; (M) girdle bands. A-C: LM; D-I, K, L: SEM; J, M: TEM. Scale bars = 10 µm (A, B, K), 5 µm (C), 1 µm (D-J, L, M).

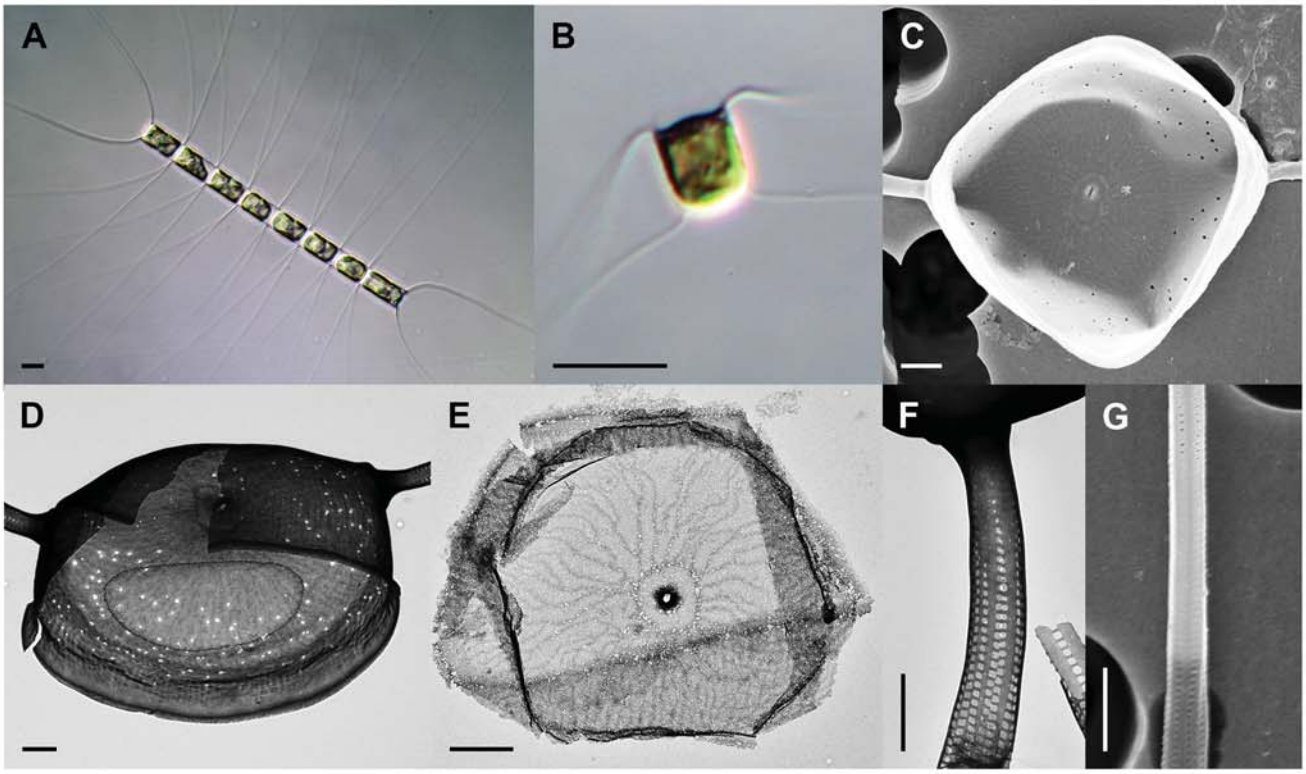

Fig. 40. *Chaetoceros* sp. clade Na26B1, strains Na26C2 (A) and Na26B1 (B-G). (A) Chain in broad girdle view; (B) single cell; (C) terminal valve in valve view, note presence of minute poroids and eccentric rimoportula; (D) terminal valve; (E) terminal valve in valve view; (F) detail of a terminal seta at the base; (G) detail of a terminal seta in the middle. A, B: LM; C, G: SEM; D-F: TEM. Scale bars = 10 µm (A-B), 1 µm (C-G).

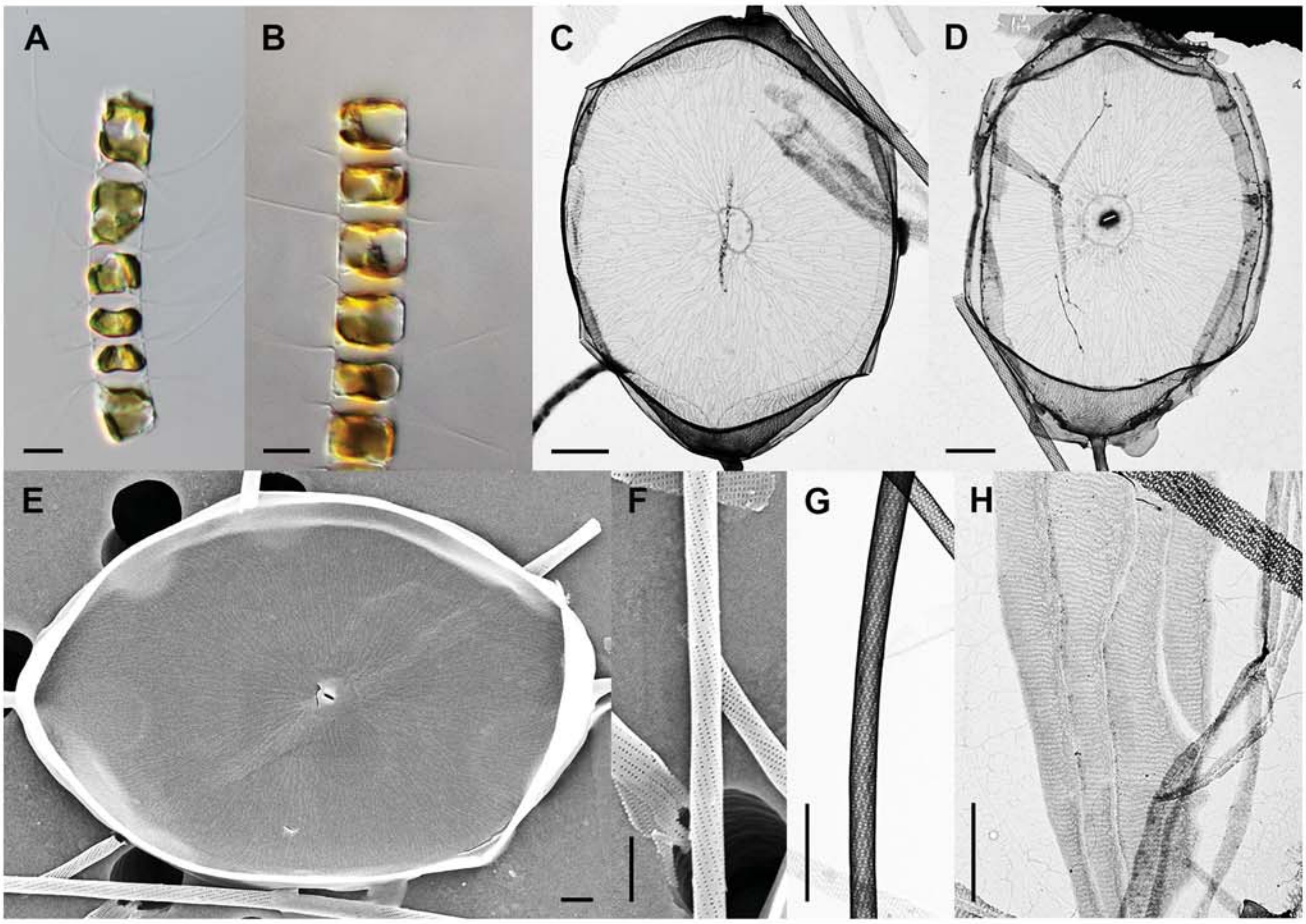

Fig. 41. *Chaetoceros* sp. clade Na11C3, strain Na11C3. (A-B) Chain in broad girdle view; (C) intercalary valve ; (D-E) t erminal valves with slit-shaped rimoportula; (F) details of the middle portion of an intercalary seta; (G) details of terminal seta in the middle; (H) girdle bands. A, B: LM; E, F SEM; C, D, G, H: TEM. Scale bars = 10 µm (A, B), 2 µm (C, D, G, H), 1 µm (E, F).

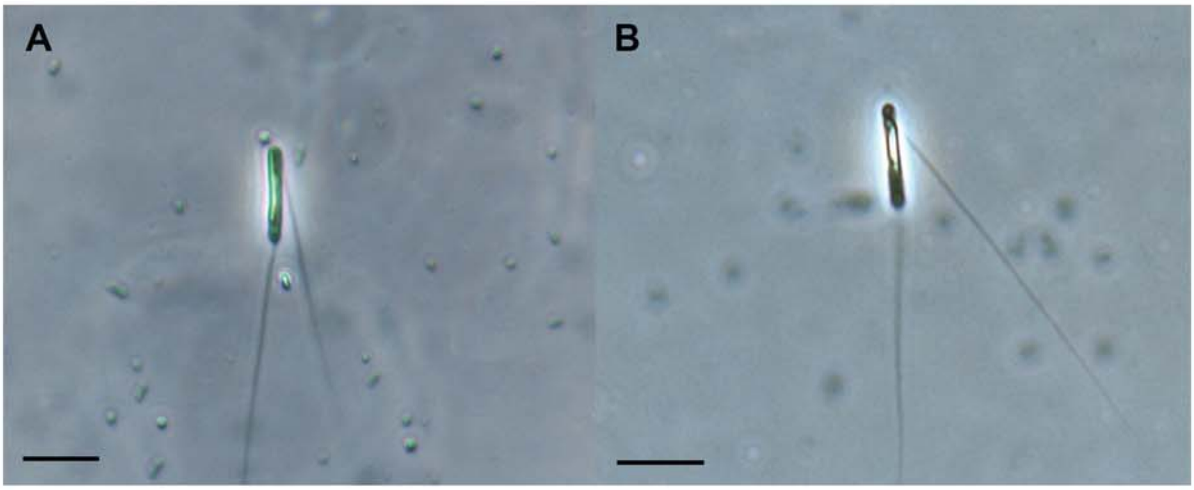

Fig. 42. *Chaetoceros throndsenii*, strain Na44C3. (A, B) LM of single heterovalvate cells with only two setae both directed towards one cell end. Scale bars = 10 µm (A, B).

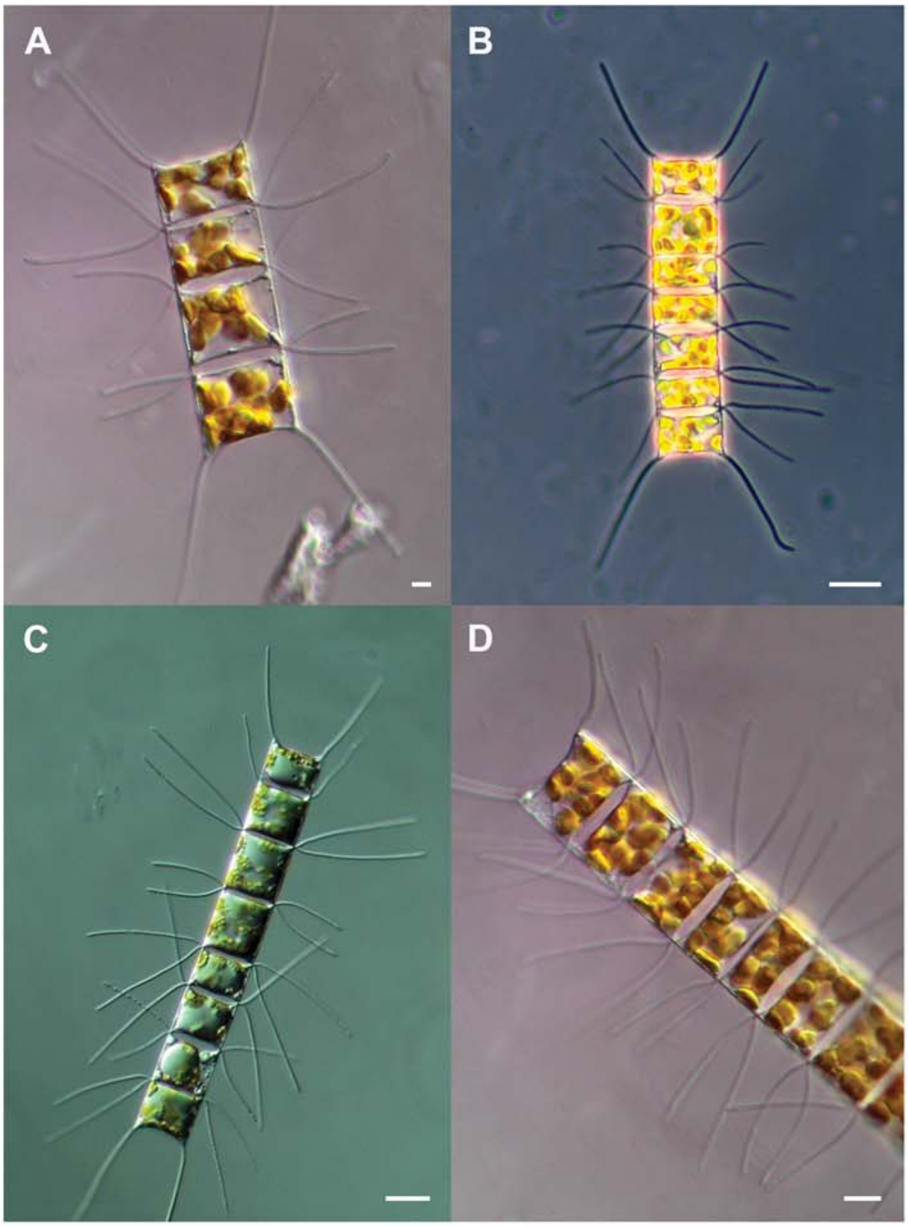

Fig. 43. *Chaetoceros lorenzianus* 1, strain Ch3B4. (A-D) LM of chains in broad girdle view. Scale bars = 20 µm (B, C), 10 µm (D), 5 µm (A).

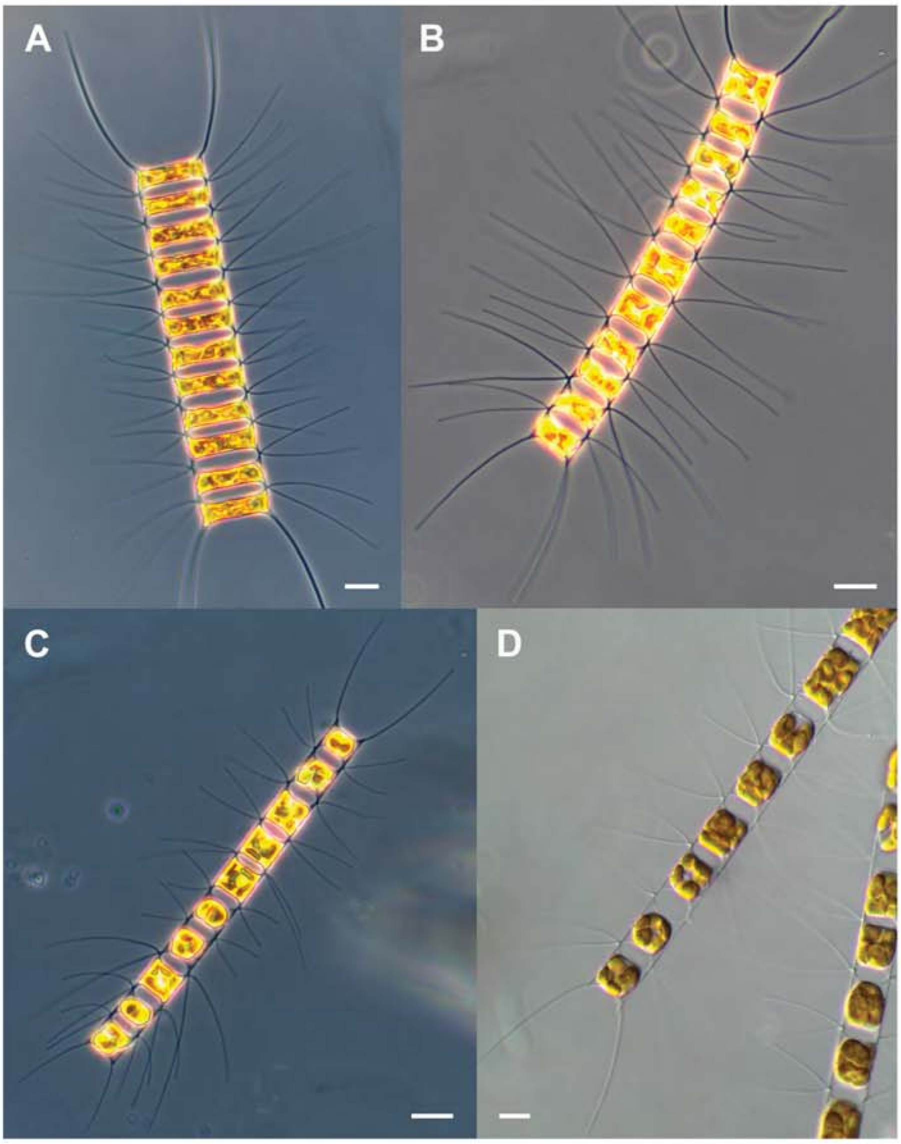

Fig. 44. *Chaetoceros lorenzianus* 2, strains CH7B4 (A), Ch2C1 (B), Ch3B2 (C), Ch13B4 (D). (A-D) LM of chains in broad girdle view. Scale bars = 20 µm (A-C), 10 µm (D).
