# Supplementary material for: Annotated 18S and 28S rDNA reference sequences of taxa in the planktonic diatom family Chaetocerotaceae
Source: PLoS One. 2018 Dec 26;13(12):e0208929. doi: 10.1371/journal.pone.0208929 (PMC6306197; doi:10.1371/journal.pone.0208929)
Supplement: S6 Fig — Figures on the left side of clades are bootstrap values (1000 replicates). (PDF) [file pone.0208929.s006.pdf]

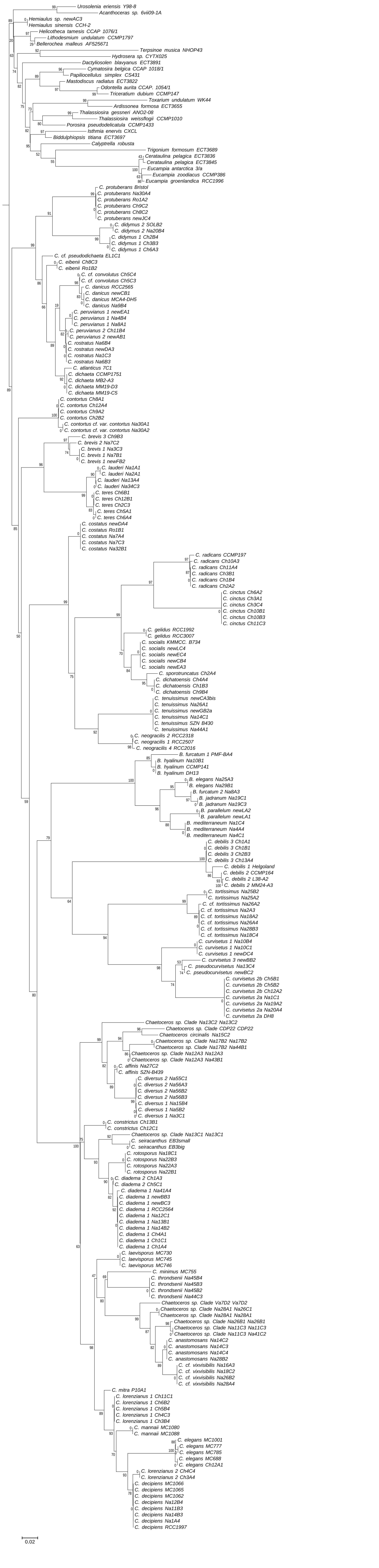
Urosolenia eriensis Y98-8
Acanthoceros sp. 6vii09-1A
Hemiaulus sp. newAC3
Hemiaulus sinensis CCH-2
Helicotheca tamesis CCAP 1076/1
Lithodesmium undulatum CCMP1797
Bellerochea malleus AF525671
Terpsinoe musica NHOP43
Hydrosera sp. CYTX025
Dactyliosolen blavyanus ECT3891
Cymatosira belgica CCAP 1018/1
Papiliocellulus simplex CS431
Mastodiscus radiatus ECT3822
Odontella aurita CCAP. 1054/1
Triceratium dubium CCMP147
Toxarium undulatum WK44
Ardissonea formosa ECT3655
Thalassiosira gessneri ANO2-08
Thalassiosira weissflogii CCMP1010
Porosira pseudodelicatula CCMP1433
Isthmia enervis CXCL
Biddulphiopsis titiana ECT3697
Calyptrella robusta
Trigonium formosum ECT3689
Cerataulina pelagica ECT3836
Cerataulina pelagica ECT3845
Eucampia antarctica 3/a
Eucampia zoodiacus CCMP386
Eucampia groenlandica RCC1996
C. protuberans Bristol
C. protuberans Na30A4
C. protuberans Ro1A2
C. protuberans Ch9C2
C. protuberans Ch8C2
C. protuberans newJC4
C. didymus 2 SOLB2
C. didymus 2 Na20B4
C. didymus 1 Ch2B4
C. didymus 1 Ch3B3
C. didymus 1 Ch6A3
C. cf. pseudodichaeta EL1C1
C. eibenii Ch8C3
C. eibenii Ro1B2
C. cf. convolutus Ch5C4
C. cf. convolutus Ch5C3
C. danicus RCC2565
C. danicus newCB1
C. danicus MCA4-DH5
C. danicus Na9B4
C. peruvianus 1 newEA1
C. peruvianus 1 Na4B4
C. peruvianus 1 Na8A1
C. peruvianus 2 Ch11B4
C. peruvianus 2 newAB1
C. rostratus Na6B4
C. rostratus newDA3
C. rostratus Na1C3
C. rostratus Na6B3
C. atlanticus 7C1
C. dichaeta CCMP1751
C. dichaeta MB2-A3
C. dichaeta MM19-D3
C. dichaeta MM19-C5
C. contortus Ch8A1
C. contortus Ch12A4
C. contortus Ch9A2
C. contortus Ch2B2
C. contortus cf. var. contortus Na30A1
C. contortus cf. var. contortus Na30A2
C. brevis 3 Ch9B3
C. brevis 2 Na7C2
C. brevis 1 Na3C3
C. brevis 1 Na7B1
C. brevis 1 newFB2
C. lauderi Na1A1
C. lauderi Na2A1
C. lauderi Na13A4
C. lauderi Na34C3
C. teres Ch6B1
C. teres Ch12B1
C. teres Ch2C3
C. teres Ch5A1
C. teres Ch6A4
C. costatus newDA4
C. costatus Ro1B1
C. costatus Na7A4
C. costatus Na7C3
C. costatus Na32B1
C. radicans CCMP197
C. radicans Ch10A3
C. radicans Ch11A4
C. radicans Ch3B1
C. radicans Ch1B4
C. radicans Ch2A2
C. cinctus Ch6A2
C. cinctus Ch3A1
C. cinctus Ch3C4
C. cinctus Ch10B1
C. cinctus Ch10B3
C. cinctus Ch11C3
C. gelidus RCC1992
C. gelidus RCC3007
C. socialis KMMCC. B734
C. socialis newLC4
C. socialis newEC4
C. socialis newCB4
C. socialis newEA3
C. sporotruncatus Ch2A4
C. dichatoensis Ch4A4
C. dichatoensis Ch1B3
C. dichatoensis Ch9B4
C. tenuissimus newCA3bis
C. tenuissimus Na26A1
C. tenuissimus newGB2a
C. tenuissimus Na14C1
C. tenuissimus SZN B430
C. tenuissimus Na44A1
C. neogracilis 2 RCC2318
C. neogracilis 1 RCC2507
C. neogracilis 4 RCC2016
B. furcatum 1 PMF-BA4
B. hyalinum Na10B1
B. hyalinum CCMP141
B. hyalinum DH13
B. elegans Na25A3
B. elegans Na29B1
B. furcatum 2 Na8A3
B. jadranum Na19C1
B. jadranum Na19C3
B. parallelum newLA2
B. parallelum newLA1
B. mediterraneum Na1C4
B. mediterraneum Na4A4
B. mediterraneum Na4C1
C. debilis 3 Ch1A1
C. debilis 3 Ch1B1
C. debilis 3 Ch2B3
C. debilis 3 Ch13A4
C. debilis 1 Helgoland
C. debilis 2 CCMP164
C. debilis 2 L38-A2
C. debilis 2 MM24-A3
C. tortissimus Na25B2
C. tortissimus Na25A2
C. cf. tortissimus Na26A2
C. cf. tortissimus Na2A3
C. cf. tortissimus Na18A2
C. cf. tortissimus Na26A4
C. cf. tortissimus Na28B3
C. cf. tortissimus Na18C4
C. curvisetus 1 Na10B4
C. curvisetus 1 Na10C1
C. curvisetus 1 newDC4
C. curvisetus 3 newBB2
C. pseudocurvisetus Na13C4
C. pseudocurvisetus newBC2
C. curvisetus 2b Ch5B1
C. curvisetus 2b Ch5B2
C. curvisetus 2b Ch12A2
C. curvisetus 2a Na1C1
C. curvisetus 2a Na19A2
C. curvisetus 2a Na20A4
C. curvisetus 2a DH8
Chaetoceros sp. Clade Na13C2 Na13C2
Chaetoceros sp. Clade CDP22 CDP22
Chaetoceros circinalis Na15C2
Chaetoceros sp. Clade Na17B2 Na17B2
Chaetoceros sp. Clade Na17B2 Na44B1
Chaetoceros sp. Clade Na12A3 Na12A3
Chaetoceros sp. Clade Na12A3 Na43B1
C. affinis Na27C2
C. affinis SZN-B439
C. diversus 2 Na55C1
C. diversus 2 Na56A3
C. diversus 2 Na56B2
C. diversus 2 Na56B3
C. diversus 1 Na15B4
C. diversus 1 Na5B2
C. diversus 1 Na3C1
C. constrictus Ch13B1
C. constrictus Ch12C1
Chaetoceros sp. Clade Na13C1 Na13C1
C. seiracanthus EB3small
C. seiracanthus EB3big
C. rotosporus Na18C1
C. rotosporus Na22B3
C. rotosporus Na22A3
C. rotosporus Na22B1
C. diadema 2 Ch1A3
C. diadema 2 Ch5C1
C. diadema 1 Na41A4
C. diadema 1 newBB3
C. diadema 1 newBC3
C. diadema 1 RCC2564
C. diadema 1 Na12C1
C. diadema 1 Na13B1
C. diadema 1 Na14B2
C. diadema 1 Ch4A1
C. diadema 1 Ch1C1
C. diadema 1 Ch1A4
C. laevisporus MC730
C. laevisporus MC745
C. laevisporus MC746
C. minimus MC755
C. throndsenii Na45B4
C. throndsenii Na45B3
C. throndsenii Na45B2
C. throndsenii Na44C3
Chaetoceros sp. Clade Va7D2 Va7D2
Chaetoceros sp. Clade Na28A1 Na26C1
Chaetoceros sp. Clade Na28A1 Na28A1
Chaetoceros sp. Clade Na26B1 Na26B1
Chaetoceros sp. Clade Na11C3 Na11C3
Chaetoceros sp. Clade Na11C3 Na41C2
C. anastomosans Na14C2
C. anastomosans Na14C3
C. anastomosans Na14C4
C. anastomosans Na28B2
C. cf. vixvisibilis Na16A3
C. cf. vixvisibilis Na18C2
C. cf. vixvisibilis Na26B2
C. cf. vixvisibilis Na28A4
C. mitra P10A1
C. lorenzianus 1 Ch11C1
C. lorenzianus 1 Ch6B2
C. lorenzianus 1 Ch5B4
C. lorenzianus 1 Ch4C3
C. lorenzianus 1 Ch3B4
C. mannaii MC1080
C. mannaii MC1088
C. elegans MC1001
C. elegans MC777
C. elegans MC785
C. elegans MC688
C. elegans Ch12A1
C. lorenzianus 2 Ch4C4
C. lorenzianus 2 Ch3A4
C. decipiens MC1066
C. decipiens MC1065
C. decipiens MC1062
C. decipiens Na12B4
C. decipiens Na11B3
C. decipiens Na14B3
C. decipiens Na1A4
C. decipiens RCC1997
0.02
